# Functional Formulation of Quantum Theory of a Scalar Field in a Metric with Lorentzian and Euclidean Signatures

**DOI:** 10.3390/e26040329

**Published:** 2024-04-12

**Authors:** Zbigniew Haba

**Affiliations:** Institute of Theoretical Physics, University of Wroclaw, Plac Maxa Borna 9, 50-204 Wroclaw, Poland; zbigniew.haba@uwr.edu.pl

**Keywords:** functional integration, quantum field theory, stochastic processes, expanding universe, quantum gravity, field correlation functions

## Abstract

We study the Schrödinger equation in quantum field theory (QFT) in its functional formulation. In this approach, quantum correlation functions can be expressed as classical expectation values over (complex) stochastic processes. We obtain a stochastic representation of the Schrödinger time evolution on Wentzel–Kramers–Brillouin (WKB) states by means of the Wiener integral. We discuss QFT in a flat expanding metric and in de Sitter space-time. We calculate the evolution kernel in an expanding flat metric in the real-time formulation. We discuss a field interaction in pseudoRiemannian and Riemannian metrics showing that an inversion of the signature leads to some substantial simplifications of the singularity problems in QFT.

## 1. Introduction

We discuss the functional integral approach to quantum field theory (QFT) in a complete analogy to the Schrödinger picture in quantum mechanics [1,2]. In contradistinction to the Heisenberg picture, we insist on states and their time evolution. The functional approach to QFT is a realization of the conventional Schrödinger picture of quantum mechanics in the space of functions of an infinite number of variables. Its efficiency has been demonstrated in refs. [3,4], in applications to quantum fields in de Sitter space. In this paper, we discuss the functional approach to QFT on general hyperbolic manifolds. In the functional formulation, we can develop the path integral methods for a calculation of expectation values earlier exploited at imaginary time as a rigorous tool in quantum mechanics [5]. We can extend these methods to real time by means of complex-valued stochastic processes. The extension to real time requires a complex extension of the configuration (field) space. For fields on a manifold, such an extension means a generalization of the path integral to complex manifolds (for some recent attempts on such generalizations, see [6,7,8,9,10,11,12,13,14]). The standard approach to field quantization on a manifold [15,16] implicitly assumes a non-unique ground state. The field can be expanded in creation–annihilation operators. These operators are defined in the Fock space which, in functional representation, is a Gaussian normalized state. The functional representation of quantum fields does not require the Fock space. We can define the Feynman path integral by means of the Wiener integral, which provides a particular Gaussian functional measure for a field configuration space (this could also be considered as a particular Fock space). Such a formulation does not require that there is a Fock space as a ground state for quantum fields. In this paper, the field is determined by a WKB Gaussian wave function as a solution of the Schrödinger equation for the canonical Hamiltonian defined by canonical quantization. We do not assume that the particular WKB wave function is normalizable. Nevertheless, we can define correlation functions in some other normalizable states with WKB phase factors. These correlation functions determine the quantum fields. The computation of correlations can be reduced to a calculation of expectation values over stochastic processes. For this reason, the functional formulation can be useful for numerical calculations. In earlier papers [17,18], we have derived a representation of the Feynman integral by means of the Brownian motion in quantum mechanics, following an approach of mathematicians Cameron and Doss [19,20]. For quantum field theory, the paths must be transformed into the paths of a quantum oscillator as a quantum field is a collection of oscillators. For a free field in Minkowski space-time, we have discussed this approach in [21], introducing a random field satisfying a stochastic equation defined by the ground state. For a free field on a general Riemannian manifold, a random field determined by a Gaussian state defined on the Riemannian manifold is needed. It can be seen that, in order to obtain an interacting field, a non-linear stochastic equation is required. The presence of noise in semiclasical quantum gravity correlation functions has been discovered in refs. [22,23]. Our approach relies on the construction of a Gaussian random field. Instead of looking for a non-linear stochastic equation of the random field, we introduce an interaction via the Feynman–Kac formula. There is no problem of such a construction on the perturbative level. Perturbation theory reduced to a polynomial expansion in quantum fields will be equivalent to the standard QFT (hence, the standard renormalization of polynomials applies). However, there arises a difficult task to show that the corresponding Feynman–Kac factor has a finite expectation value with respect to the Wiener measure. Then, the functional integral over the solutions defining the correlation functions must be finite. We show that a change in the signature of the spatial metric (the time remains real) can substantially facilitate the proof of integrability. The problem has been studied in constructive quantum field theory in the Euclidean framework. In the Feynman integral formulation (real time), we deal with oscillatory integrals. In such a case, some stability problems can be avoided. We assume that time is well-defined in an evolution of the quantum scalar field on a classical gravitational background. The appearance of time and the Schrödinger evolution in a semiclassical framework for quantum gravity have been discussed in [24,25,26,27,28]. We repeat (in a somewhat modified way) a derivation of the Schrödinger equation from the Wheeler–DeWitt equation [29] in Appendix H. We hope that the study of the Schrödinger equation in an external metric can help to solve the Wheeler–DeWitt constraint in quantum gravity.

The plan of this paper is the following. First, we explain the mathematically rigorous probabilistic method [30,31] of solving the imaginary time Schrödinger equation for a quantum (Euclidean) scalar field as a perturbation of the ground state solution (Section 2). Then, in Section 3, the method is extended by an analytic continuation to apply to solutions for the real-time Schrödinger equation. We discuss the functional Schrödinger equation for a scalar field on a globally hyperbolic manifold [32,33]. We consider general complex metrics which may arise as saddle points in an average over metrics in quantum gravity. In Section 4, we show that a free scalar quantum field has the Schrödinger wave function solution, which is a pure WKB Gaussian phase if the initial state is a pure Gaussian phase. Using this Gaussian solution, we construct a random wave field (as a solution of a stochastic equation), which allows for the construction of a general solution of the Schrödinger equation as an expectation value over the Brownian motion. The solution can be calculated in a simple way if the initial state is of the form of the Gaussian factor times a mild perturbation. It can have an explicit form if the manifold has a large group of symmetry. In Section 5, we discuss the scalar field on de Sitter space-time and on its Euclidean continuation (the sphere $S^{4}$). Stochastic equations for the de Sitter field and fields in an expanding (homogeneous) universe are discussed in Section 6. The large symmetry group allows for an expansion in terms of eigenfunctions of the algebra of this group. In this section, some field correlations are calculated. The explicit Gaussian solutions of the Schrödinger equation are not available for general initial conditions. In Section 6, we derive an asymptotic formula for the solution at a large angular momentum. An analogous formula at large momenta for the homogeneous expanding metric is obtained in Appendix B. The asymptotic formulas allow for an approximate calculation of correlation functions of stochastic fields. In Section 7, the free field time evolution is expressed by an evolution kernel. Then, a computation of multi-time correlation functions can be reduced to Gaussian integrals with the evolution kernels. The method can be extended to interacting fields by means of the Feynman–Kac formula. The correlation functions determine QFT (Wightman’s reconstruction). In Section 8, Section 9 and Section 10, the power-law evolution of massless scalar fields is discussed. The most detailed results concern the free field in a radiation background (Section 10) with the spatial metric $g_{jk}≃\delta_{jk}t$, which changes signature at t=0. In Section 11 and Section 12, we discuss polynomial and trigonometric interactions. We show, in Section 11, that for a finite mode approximation the expectation value of the Feynman–Kac factor can be finite in the Wiener integral formulation of the Feynman integral when we average the potentials over the random fields constructed from the Gaussian WKB wave functions in the earlier sections. In Section 12, it is shown that an inversion of the spatial signature allows to show that the expectation value of the Feynman–Kac factor is finite for an infinite number of modes. The inversion of the signature may be considered either as a technical tool or as a quantum effect of an average of the functional integral over quantum metrics. Some supplementary materials are located in the appendices. Appendix C, Appendix D and Appendix E describe for the convenience of the reader some simplified models (discussed earlier) of the ones studied in the main part. Appendix A, Appendix B, Appendix F and Appendix G contain some additions to the results in the main text, which illustrate the method of stochastic equations (this is just a transformation of sample paths). We have studied the Brownian motion formulation of quantum physics for some time. The motivation was twofold: 1. to formulate the Feynman integral as a rigorous mathematical tool, and 2. to make quantum correlations susceptible to standard simulations by means of random variables. We obtained the stochastic formulation of the Feynman integral first in quantum mechanics [17,18,34]. The stochastic free field is introduced in [21,35,36]. The inversion of the metric in a radiation background is discussed in [37]. In this paper, we present a comprehensive approach to the stochastic representation of QFT (beyond the homogeneous expanding metric), including the previously studied models as special cases. The choice of the stochastic field is not unique. It depends on the selection of the Gaussian WKB state. We discuss, in detail, the form of the field which enables a construction of a well-defined (integrable) Feynman–Kac factor for the field theory with an interaction.

## 2. The Imaginary Time Schrödinger Equation

First, we discuss the standard canonical field theory in a mathematically rigorous imaginary time formulation [30]. The Hamiltonian is defined as

(1) $$H=\frac{1}{2}\int dx\left(\Pi^{2}+{\left(\nu \Phi \right)}^{2}\right)+\int dxV\left(\Phi \right)=H_{0}+V,$$

where

(2) $$\nu =\sqrt{-▵+M^{2}}.$$

The canonical momentum Π satisfies the commutation relations with the field Φ

(3) [Φ(x),Π(y)]=iℏδ(x−y).

Let $\psi_{t}^{g}$ be a solution (usually the ground state) of the imaginary time Schrödinger equation

(4) $$-\hbar \partial_{t}\psi =H\psi .$$

Let us consider the general solution of the Schrödinger Equation (4) with the initial condition $\psi =\psi_{0}^{g}\chi$

(5) $$\psi_{t}=\psi_{t}^{g}\chi_{t}.$$

Then, $\chi_{t}$ satisfies the equation

(6) $$-\hbar \partial_{t}\chi =\int dx\left(\frac{1}{2}\Pi^{2}-\left(\Pi \ln\psi_{t}^{g}\right)\Pi \right)\chi$$

with

(7) $$\Pi \left(x\right)=-i\hbar \frac{\delta }{\delta \Phi (x)}.$$

$\psi_{t}^{g}$ may be an arbitrary solution of the Schrödinger Equation (4) (with an arbitrary initial condition). The efficiency of the representation ((5) and (6)) depends on the choice of $\psi_{t}^{g}$ and the assumption that initial states are under consideration (these will be the WKB states). It can be seen that, in Equation (6), the kinetic term ${\left(\nu \Phi \right)}^{2}$, as well as the potential *V*, are absent. Equation (6) is a diffusion equation in infinite dimensional spaces [31]. The solution of Equation (6) can be expressed as [38]

(8) $$\chi_{t}\left(\Phi \right)=E\left[\chi \left(\Phi_{t}\left(\Phi \right)\right)\right],$$

where $\Phi_{t}\left(\Phi \right)$ is the solution of the stochastic equation (t≥s≥0)

(9) $$d\Phi_{s}\left(x\right)=\hbar \frac{\delta }{\delta \Phi_{s}\left(x\right)}\ln\psi_{t-s}^{g}dt+\sqrt{\hbar }dW_{s}\left(x\right)$$

with the initial condition Φ. E[…] denotes an expectation value with respect to the Wiener process (Brownian motion) with the mean zero and the covariance (t≥0, s≥0)

(10) $$E\left[W_{t}\left(x\right)W_{s}\left(y\right)\right]=min\left(t,s\right)\delta \left(x-y\right).$$

The correlation functions of the quantum Euclidean field in the state $\psi_{0}^{g}$ can be expressed by the correlation functions of the stochastic process $\Phi_{t}$. If we could find a particular solution of the Schrödinger equation and solve the non-linear stochastic differential Equation (9), then the problem of solving QFT and calculating the field correlation functions could be reduced to a calculation of expectation values with respect to the Wiener process. We do not know any solution of the Schrödinger equation for scalar field theory (for a potential V(Φ) which is not quadratic). However, in field theories with large symmetry, this could be possible (let us mention Chern–Simons states in gauge theories [6] and Kodama states in gravity [39]). The imaginary time in this section has a rigorous mathematical formulation for super-renormalizable field theories in two dimensions [30]. In general, for arbitrary states $\psi_{t}^{g}$, higher dimensions and real time, we expect difficulties with a derivation of solutions of Equation (9) and their renormalization. We can manage in this paper linear stochastic equations (corresponding to Gaussian $\psi_{t}^{g}$) for free field theory. Then, the interaction is introduced as usual by means of the Feynman–Kac formula.

Let us consider the simplest example: the free field. Then, the ground state is

(11) $$\psi^{g}=Z^{-1}\exp\left(-\frac{1}{2\hbar }\Phi \nu \Phi \right),$$

where *Z* is the state normalization. The stochastic Equation (9) reads

(12) $$d\Phi_{t}=-\nu \Phi_{t}dt+\sqrt{\hbar }dW_{t}.$$

The solution is (with the initial condition Φ at $t_{0}$)

(13) $$\Phi_{t}=\exp\left(-\nu \left(t-t_{0}\right)\right)\Phi +\sqrt{\hbar }\int_{t_{0}}^{t}\exp\left(-\nu \left(t-s\right)\right)dW_{s}.$$

We calculate

(14) $$\begin{array}{c} \int d\Phi \psi_{g}^{2}E\left[\exp(\int dtdxf_{t}\left(x\right)\Phi_{t}\left(x\right))\right]\\ =\exp\left(\frac{1}{2}\int dtdt^{\prime }\left(f_{t},{\left(2\nu \right)}^{-1}\exp(-\nu |t-t^{\prime }\left|\right)f_{t^{\prime }}\right)\right) \end{array}$$

On the rhs of Equation (14), we have the generating functional for the correlation functions of the quantum Euclidean free field.

As a time-dependent solution of Equation (4) (with V=0), we may consider

(15) $$\psi_{t}^{g}=A\left(t\right)\exp\left(i\frac{1}{2\hbar }\Phi \Gamma_{t}\Phi \right).$$

In Equation (15), Γ is an operator with an integral kernel Γ(x,y). We can derive an equation for this operator, demanding that (15) is the solution of the Schrödinger Equation (4). Then, $\psi_{t}^{g}$ is the solution of the free imaginary time Schrödinger Equation (4) (V=0) if

(16) $$i\partial_{t}\Gamma +\Gamma^{2}+\nu^{2}=0.$$

Equation (16) is equivalent to

(17) $$(\partial_{t}^{2}-\nu^{2})u=0$$

if

(18) $$-i\Gamma =u^{-1}\frac{d}{dt}u.$$

The general solution of Equation (17) is

(19) $$u=C_{1}\sinh\left(\nu t\right)+C_{2}\cosh\left(\nu t\right)$$

If $C_{1}=C_{2}$, then, from Equations (15), (18) and (19), we obtain the ground state solution (11). We discuss the case $C_{1}=0$ in Appendix A. It defines another field $\Phi_{t}$ whose correlation functions are equal to the ones of the standard free Euclidean field, but in another time-dependent state (15). We obtain another realization of the solution (5) of the Schrödinger equation for the free field.

With the potential *V* in Equation (1), the solution of the Schrödinger Equation (4) reads [5,38] (the Feynman–Kac formula requires V(Φ) to be bounded from below)

(20) $$\chi_{t}\left(\Phi \right)=E\left[\exp\left(-\frac{1}{\hbar }\int_{0}^{t}V\left(\Phi_{s}\right)ds\right)\chi \left(\Phi_{t}\left(\Phi \right)\right)\right].$$

At the end of this section, we wish to point out some problems with the definition of the Feynman–Kac integral (20) in Euclidean field theory (even if V(Φ) is bounded from below). We consider in subsequent sections exponential potentials

V(Φ)=λ∫dxexp(αΦ(x)),

where $B\subset R^{d}$ is a bounded region in $R^{d}$. It can be seen that higher orders of the perturbation expansion in λ of the normal ordered exponentials in the Feynman–Kac Formula (20) are divergent if the dimension of the space-time is d≥3, e.g., in the second order (where :-: denotes the normal ordering), we obtain

$$\begin{array}{c} \lambda^{2}\int_{0}^{t}ds\int_{0}^{t}d\tau \int_{B}dx\int_{B}dyE\left[:\exp\left(\alpha \Phi_{s}\left(x\right)\right)::\exp\left(\alpha \Phi_{\tau }\left(y\right)\right):\right]\\ =\lambda^{2}\int_{0}^{t}ds\int_{0}^{t}d\tau \int_{B}dx\int_{B}dy\exp\left(\alpha^{2}E\left[\Phi_{s}\left(x\right)\Phi_{\tau }\left(y\right)\right]\right). \end{array}$$

The two-point function is positive and at short distances

$$E\left[\Phi_{s}\left(x\right)\Phi_{\tau }\left(y\right)\right]≃{\left({\left(s-\tau \right)}^{2}+{\left(x-y\right)}^{2}\right)}^{1-\frac{d}{2}}.$$

The $\lambda^{2}$ term of the perturbation series of Equation (20) is infinite if α is real and d≥3 (this follows from $\exp\left(x\right)\geq 1+\frac{x^{4}}{4!}$ for x≥0).

If α=iβ is purely imaginary, then

$$\begin{array}{c} \lambda^{2}t^{2}{\left|B\right|}^{2}\geq \lambda^{2}\int_{0}^{t}ds\int_{0}^{t}d\tau \int_{B}dx\int_{B}dyE\left[:\exp\left(i\beta \Phi_{s}\left(x\right)\right)::\exp\left(i\beta \Phi_{\tau }\left(y\right)\right):\right]\\ =\lambda^{2}\int_{0}^{t}ds\int_{0}^{t}d\tau \int_{B}dx\int_{B}dy\exp\left(-\beta^{2}E\left[\Phi_{s}\left(x\right)\Phi_{\tau }\left(y\right)\right]\right)\\ \geq \lambda^{2}t^{2}{\left|B\right|}^{2}\exp\left(-\beta^{2}t^{-2}{\left|B\right|}^{-2}\int_{0}^{t}ds\int_{0}^{t}d\tau \int_{B}dx\int_{B}dyE\left[\Phi_{s}\left(x\right)\Phi_{\tau }\left(y\right)\right]\right) \end{array}$$

from Jensen inequality. We obtain an upper bound and a non-zero lower bound if d<6. However, if we form real potentials as, e.g., λcosβΦ, then at the order $\lambda^{2}$, there will be terms without an upper bound because of the multiplication of the terms exp(iβΦ(x)) with exp(−iβΦ(y)).

## 3. Real Time: The Free Field on a Manifold

We consider a globally hyperbolic manifold [33] with a choice of coordinates such that the metric is of the form

(21) $$ds^{2}=g_{00}dx^{0}dx^{0}-g_{jk}dx^{j}dx^{k}.$$

The Lagrangian of the free field is [40]

(22) $$L=\frac{1}{2}\sqrt{-g}g^{\mu \nu }\partial_{\mu }\phi \partial_{\nu }\phi -\frac{M^{2}}{2}\sqrt{-g}\phi^{2},$$

where $g=\det(g_{\mu \nu })$. The canonical momentum is

(23) $$\Pi =g^{00}\sqrt{-g}\partial_{0}\phi .$$

From the Lagrangian (22), we drive the canonical Hamiltonian

(24) $$\begin{array}{c} H\left(t\right)\equiv \int dxH\left(g,x\right)=\int dx\left(\Pi \partial_{0}\phi -L\right)\\ =\int dx\left(\frac{1}{2}g_{00}\frac{1}{\sqrt{-g}}\Pi^{2}+\frac{1}{2}\sqrt{-g}g^{jk}\partial_{j}\phi \partial_{k}\phi +\frac{M^{2}}{2}\sqrt{-g}\phi^{2}\right). \end{array}$$

In subsequent sections, we shall discuss the Lagrangian (22) and the Hamiltonian (24) for the metric tensors $g_{\mu \nu }$, which can arise as saddle points in the Feynman path integral. Such metrics satisfy Einstein equations, but they do not fulfill the requirement −g>0. We must choose the square root $\sqrt{-g}$ in such a way (see [6,7,9,10,11,12]) that the path integral and the Schrödinger equation

(25) $$i\hbar \partial_{t}\psi_{t}=H\left(t\right)\psi_{t}$$

are well-defined. The solution of Equation (25) must define an operator which is a contraction in a Hilbert space (otherwise $\psi_{t}$ may have an infinite norm). We assume (as in [7]) that, for t≥0, the metric is real and Lorentzian. In the past, the stationary points of the Lagrangian with matter satisfying some positive energy conditions [32] will necessarily exhibit a Big Bang singularity (then, e.g., $\frac{1}{\sqrt{-g}}$ is not defined). We assume that the solutions of Einstein equations have a continuation to t<0, but do not satisfy the requirements of the classical general relativity (they may be Euclidean or even complex). So, for positive time, we shall have the Schrödinger equation, whereas for negative time with the Hamiltonian (24), a diffusion-type Equation (a contraction [41]). The necessary condition for a contraction at t<0 is that the infinite dimensional diffusion generator has the imaginary part of the second order differential operator, which is a positive operator. From the Hamiltonian (24), we can see that this will be the case if

$$g_{00}\frac{1}{\sqrt{-g}}=R+iI\;\mathrm{where}\;I\geq 0,$$

where *R* and *I* are the real and imaginary parts of a complex function. In such a case, we write $H=iH_{E}$ and write the Schrödinger Equation (25) as a diffusion equation

(26) $$\hbar \partial_{t}\psi_{t}=H_{E}\left(t\right)\psi_{t}$$

In our models, we choose $\sqrt{-g}=-i\sqrt{\left|g\right|}$. Then, $H_{E}$ will be (on a formal level) a Hermitian positive operator. Hence, Equation (26) defines a contraction (diffusion) for t<0. For the inverted metric, the mass term has an opposite sign to the kinetic term. So, we invert its sign as $M^{2}\to -\mu^{2}$.

In Section 10, we discuss a solution for a homogeneous radiation metric $a^{2}≃t$ which leads to an infinite energy density when t→0, but it is also a solution of Einstein equations at t<0 with $a^{2}<0$. Examples which have a continuation from the real time in de Sitter space to the four-sphere at the imaginary time have been discussed in [7,8,14].

Let us still mention another interpretation of the Hamiltonian (24) for an inverted metric

(27) $$\begin{array}{c} H\left(t\right)=\int dx\left(\Pi \partial_{0}\phi -L\right)\\ =\int dx\left(\frac{1}{2}g_{00}\frac{1}{\sqrt{\left|g\right|}}\Pi^{2}+\frac{1}{2}\sqrt{\left|g\right|}g^{jk}\partial_{j}\phi \partial_{k}\phi +\frac{M^{2}}{2}\sqrt{\left|g\right|}\phi^{2}\right). \end{array}$$

Such a modification of the Hamiltonian (24) transforms the Schrödinger evolution with the Lorentzian metric into the one with an Euclidean metric. It is still unitary. The inversion of the spatial metric in Equation (27) (so that $g^{jk}$ is negatively definite) at t=0 is an analog of a transformation of the oscillator for t≥0 into an upside-down oscillator for t<0 [36,42,43]. The Hamiltonian (27) results from the replacement $\sqrt{-g}\to \sqrt{\left|g\right|}$ in the Lagrangian (22) as a possible candidate for quantum gravity.

Inserting the WKB wave function (15) in Equation (25), we obtain an equation for the operator Γ and the normalization coefficient *A*

(28) $$\partial_{t}\Gamma_{t}+\Gamma_{t}J\Gamma_{t}+M=0,$$

(29) $$\partial_{t}\ln A=-\frac{1}{2}\int dx\frac{g_{00}}{\sqrt{-g}}\Gamma_{t}\left(x,x\right),$$

where

(30) $$J\left(y,y^{\prime }\right)=\frac{g_{00}}{\sqrt{-g}}\delta \left(y,y^{\prime }\right)$$

and M is the differential operator

(31) $$M=J^{-1}K^{2}=M^{2}\sqrt{-g}-\partial_{j}\sqrt{-g}g^{jk}\partial_{k}.$$

where the operator [40]

$$K^{2}=-g_{00}\frac{1}{\sqrt{-g}}\partial_{j}g^{jk}\sqrt{-g}\partial_{k}+g_{00}M^{2}$$

is self-adjoint in $L^{2}\left(d\mu \right)$ with respect to the measure

$$d\mu =g^{00}\sqrt{-g}dx.$$

Let us define the operator

(32) $$G_{t}=\exp\left(\int^{t}J_{s}\Gamma_{s}ds\right).$$

$\Gamma_{t}$ can be expressed by $G_{t}$

(33) $$\Gamma_{t}=J_{t}^{-1}\partial_{t}GG^{-1}=J_{t}^{-1}\partial_{t}\ln G.$$

$G_{t}$ satisfies a linear operator equation

(34) $$\partial_{t}^{2}G=\partial_{t}JJ^{-1}\partial_{t}G-JMG.$$

Let us note that $\sqrt{-g}$ cancels in JM in Equation (34). Hence, this equation does not depend on the interpretation of $\sqrt{-g}$ for an inverted metric. It can be shown that the operator Equation (34) coincides with the wave equation corresponding to the Lagrangian (22). Then, as discussed in Section 2, ΓΦ is the drift in the stochastic Equation (9). For the general metric (21), it is not simple to find a solution Γ. In subsequent sections, we find explicit solutions assuming the invariance either with respect to the de Sitter group or under translations (homogeneous spatially flat manifold). Some information about Γ is needed in order to determine whether $\psi_{t}^{g}$ is square integrable. We need to know Γ for a construction of an interaction in such a way that the Feynman–Kac factor has a finite expectation value.

We express the Feynman path integral solution of the Schrödinger Equation (25) (V=0) with the initial condition

(35) $$\psi_{0}^{g}=\exp\left(\frac{i}{\hbar }S_{0}\right)$$

in the form

(36) $$\begin{array}{c} \psi_{t}^{g}\left(\Phi \right)=\int d\Phi \left(.\right)\exp(\frac{i}{2\hbar }\int d\tau dx\left(\sqrt{-g}g^{\mu \nu }\partial_{\mu }\Phi \partial_{\nu }\Phi -M^{2}\sqrt{-g}\Phi^{2}\right)\exp(\frac{i}{\hbar }S_{0}\left(\Phi_{t}\left(\Phi \right)\right). \end{array}$$

Formula (36) is well-established with real values of $\sqrt{-g}$ as a solution of Equation (25) [38]. However, a formal derivation of Equation (36) does not use any assumption on the signature of the metric, as long as the exponential (36) is bounded as a function of Φ. As in the Hamiltonian Equation (25), we assume that, for t≥0, the metric is real and Lorentzian. For t<0, we admit complex $g_{\mu \nu }$ and complex $\sqrt{-g}$. If we require that the quadratic factor ${\left(\partial_{0}\Phi \right)}^{2}$ in the Feynman integral (36) does not grow for t<0, then the conditions upon the metric will be

$$\sqrt{-g}g^{00}=\tilde{R}+i\tilde{I},\;\mathrm{where}\;\tilde{I}\leq 0.$$

If the spatial part in the action in the exponential (36) is to be positive definite for t<0, then

$$\sqrt{-g}g^{jl}=R^{jl}+iI^{jl},$$

where $I^{jl}$ is a positive definite matrix. If $\sqrt{-g}=-i\sqrt{\left|g\right|}$, then the real part of the integral of the quadratic term ${\left(\partial_{0}\Phi \right)}^{2}$ in Equation (36) is negative, and if $g^{jl}$ is negatively definite (inverted metric), then the real part of the spatial quadratic term in (36) is also negative. The mass term has an opposite sign; hence, if it is to be negative, we must change $M^{2}\to -\mu^{2}$ for t<0. The conditions for the path integral coincide with the ones derived from the Hamiltonian (below Equation (25)). We shall still discuss the Schrödinger equations and path integrals in detail in specific models in subsequent sections. The requirements for the complex metric have been discussed in [6,12,13]. They look different then the ones required for the Hamiltonian at the beginning of this section. We shall discuss these conditions in Section 9 and Section 10, when we discuss a change in signature.

We assume that $S_{0}$ is a quadratic form in Φ

(37) $$S_{0}\left(\Phi \right)=\left(\Phi ,\Gamma_{0}\Phi \right),$$

where (,) denotes the scalar product in $L^{2}\left(dx\right)$. We solve the Schrödinger Equation (25) by means of the stationary phase method. We expand the Feynman integral (36) around the stationary point $\phi_{s}^{c}\left(\Phi \right)$. The stationary point is obtained as a solution $\phi_{s}^{c}\left(\Phi \right)$ of the Cauchy problem with the initial field value Φ, and the final boundary condition on the time derivative [44]

(38) $$\frac{d\phi_{t}^{c}}{dt}=-\frac{\delta S_{0}\left(\phi \right)}{\delta \phi }\left(\phi_{t}^{c}\right).$$

The solution $\phi_{t}^{c}\left(\Phi \right)$ is linear in Φ. We write

(39) $$\Phi_{s}=\phi_{s}^{c}\left(\Phi \right)+\sqrt{\hbar }\phi_{s}^{q}.$$

Then,

(40) $$\psi_{t}^{g}\left(\Phi \right)=A_{t}\exp\left(\frac{i}{\hbar }S_{t}\left(\phi_{c}\left(\Phi \right)\right)\equiv A_{t}\exp\left(\frac{i}{2\hbar }\Phi ,\Gamma_{t}\Phi \right)\right).$$

The classical action $S_{t}$ is a bilinear form in Φ defined by a real kernel $\Gamma_{t}\left(x,y\right)$ (if $S_{0}$ is a real function), $A_{t}$ is expressed by the determinant of an operator defined by the quadratic form in $\phi^{q}$. The determinant depends only on time and the metric. It follows that if $S_{0}$ is real, then $S_{t}$ is a real bilinear form (if $S_{0}$ is complex, then $S_{t}$ is also complex). Hence, $\psi_{t}^{g}$, as a function of Φ, is a pure phase factor.

## 4. Gaussian Solution of the Schrödinger Equation and Linear Stochastic Equations

We approach the quantum field theory (QFT) in Minkowski space-time by means of stochastic equations [21,34,35]. The stochastic equations determine the solution of the Schrödinger equation as in Equation (8). We continue the imaginary time in Equations (8) and (9) to the real time [21]

(41) $$d\Phi_{s}\left(x\right)=i\hbar \frac{\delta }{\delta \Phi_{s}\left(x\right)}\ln\psi_{t-s}^{g}dt+\sqrt{i\hbar }dW_{s}\left(x\right),$$

where $\sqrt{i}=\exp\left(i\frac{\pi }{4}\right)=\frac{1}{\sqrt{2}}\left(1+i\right)$. Let $\hat{\Phi }$ be the relativistic quantum free field. Then, the generating functional of the time-ordered correlation functions of $\hat{\Phi }$ in the vacuum $\psi^{g}$ (11) can be expressed [21] by the solution $\Phi_{t}\left(\Phi \right)$ of the stochastic Equation (41)

(42) $$\begin{array}{c} \left(\psi_{g},T\left(\exp\int dtdx{\hat{\Phi }}_{t}\left(x\right)f_{t}\left(x\right)\right)\psi_{g}\right)=\int d\Phi \psi_{g}^{2}\left(\Phi \right)E\left[\exp(\int dtdxf_{t}\left(x\right)\Phi_{t}\left(\Phi ,x\right))\right]\\ =\exp\left(\frac{1}{2}\int dtdt^{\prime }\left(f_{t},{\left(2\omega \right)}^{-1}\exp(-i\omega |t-t^{\prime }\left|\right)f_{t^{\prime }}\right)\right), \end{array}$$

where T(…) is the time-ordered product and (f,g) denotes the scalar product in $L^{2}\left(dx\right)$.

When the initial condition is $\psi =\psi_{0}^{g}\chi$, and $\psi_{t}^{g}$ is the solution of the Schrödinger Equation (25), then χ solves the equation

(43) $$i\hbar \partial_{t}\chi_{t}=\int dx\left(\frac{1}{2}\Pi^{2}+\left(\Pi \ln\psi_{t}^{g}\right)\Pi \right)\chi_{t}.$$

If the solution $\psi_{t}^{g}$ of the Schrödinger equation for quantum fields defined on the Minkowski space-time is of the form (40) then Equation (41) reads [38]

(44) $$d\Phi_{s}=-\Gamma_{t-s}\Phi_{s}ds+\sqrt{i\hbar }dW_{s}.$$

Let $\Phi_{s}\left(\Phi \right)$ be the solution of Equation (44) with the initial condition Φ. If χ is a holomorphic function, then the solution of the Schrödinger Equation (25) is

(45) $$\psi_{t}=\psi_{t}^{g}E\left[\chi \left(\Phi_{t}\left(\Phi \right)\right)\right].$$

With the interaction $V_{t}$, the Feynman formula reads

(46) $$\psi_{t}=\psi_{t}^{g}E\left[\exp\left(-\frac{i}{\hbar }\int_{0}^{t}V_{t-s}\left(\Phi_{s}\right)ds\right)\chi \left(\Phi_{t}\left(\Phi \right)\right)\right].$$

The solution of the stochastic equation determines the free field correlation functions (V=0)

(47) $$\begin{array}{c} \left(\psi_{0}^{g},F_{1}\left(\Phi_{t}\right)F_{2}\left(\Phi \right)\psi_{0}^{g}\right)=\int d\Phi {\left|\psi_{t}^{g}\left(\Phi \right)\right|}^{2}F_{1}\left(\Phi \right)E\left[F_{2}\left(\Phi_{t}\left(\Phi \right)\right)\right]. \end{array}$$

For more general states $\psi^{g}\chi$ and V≠0, we have

(48) $$\begin{array}{c} \left(\psi_{0}^{g}\chi ,F_{1}\left(\Phi_{t}\right)F_{2}\left(\Phi \right)\psi_{0}^{g}\chi \right)=\int d\Phi {\left|\psi_{t}^{g}\left(\Phi \right)\right|}^{2}F_{1}\left(\Phi \right)\\ E{\left[\chi \left(\Phi_{t}\left(\Phi \right)\right)\exp\left(-\frac{i}{\hbar }\int_{0}^{t}V_{t-s}\left(\Phi_{s}\right)ds\right)\right]}^{*}\\ \times E\left[F_{2}\left(\Phi_{t}\left(\Phi \right)\right)\chi \left(\Phi_{t}\left(\Phi \right)\right)\exp\left(-\frac{i}{\hbar }\int_{0}^{t}V_{t-s}\left(\Phi_{s}\right)ds\right)\right]. \end{array}$$

We generalize these correlation functions in Section 7 to multitime correlation functions after a derivation of a formula for the evolution propagator. In principle, QFT (with the Hilbert space and quantum fields) can be determined by the correlation functions (Wightman construction).

## 5. De Sitter Space

In this and subsequent sections, we obtain quantum field evolution on the Lorentzian and Euclidean backgrounds. First, we discuss particular solutions (de Sitter and the sphere $S^{4}$) of Einstein equations with a cosmological constant (obtained in [7,8]). De Sitter space-time can describe an early inflationary stage of the universe, as well as the final stage of an acceleration driven by dark energy. The authors [7,8] glue together the Lorentzian solution for positive time with the Euclidean solution ($S^{4}$) for an imaginary time. We consider several coordinate systems on de Sitter space [7,45] which can be considered as a submanifold of the complex quadric

(49) $$z_{1}^{2}+z_{2}^{2}+z_{3}^{3}+z_{4}^{2}+z_{5}^{2}=\frac{1}{H^{2}},$$

where *H* has the meaning of the Hubble constant. We first consider a real form of the quadric (49), defining the de Sitter space

(50) $$x_{1}^{2}+x_{2}^{2}+x_{3}^{3}+x_{4}^{2}-x_{5}^{2}=\frac{1}{H^{2}}.$$

In the coordinates (t,ω), where $\omega \in S^{3}$, the metric on the hyper-sphere (50) is

(51) $$ds^{2}=dt^{2}-\frac{1}{H^{2}}\cosh^{2}\left(Ht\right)d\omega^{2},$$

where $d\omega^{2}$ is the metric on $S^{3}$.

In conformal coordinates

(52) $$\cos\left(\tau \right)=\frac{1}{\cosh(Ht)},$$

where $0<\tau <\frac{\pi }{2}$, we obtain the metric

(53) $$ds^{2}=\frac{1}{H^{2}\cos^{2}\left(\tau \right)}\left(d\tau^{2}-d\omega^{2}\right).$$

The Euclidean version of the manifold (53) describes the four-dimensional sphere of radius $\frac{1}{H}$

$$x_{1}^{2}+x_{2}^{2}+x_{3}^{3}+x_{4}^{2}+x_{5}^{2}=\frac{1}{H^{2}}$$

Then, the metric is

(54) $$ds^{2}=dt^{2}+\frac{1}{H^{2}}\cos^{2}\left(Ht\right)d\omega^{2}.$$

The introduction of conformal coordinates

(55) $$\cosh\left(\tau \right)=\frac{1}{\cos(Ht)},$$

where $-\frac{\pi }{2H}<t\leq 0$ (we choose negative time in the Euclidean domain) gives the metric

(56) $$ds^{2}=\frac{1}{H^{2}\cosh^{2}\left(\tau \right)}\left(d\tau^{2}+d\omega^{2}\right).$$

We can also introduce spatially flat coordinates describing the expanding universe (which will be discussed in detail in subsequent sections)

$$ds^{2}=dt^{2}-a^{2}dx^{2}.$$

The expanding flat metric in coordinates which cover the half of de Sitter space-time (visible by an observer at the origin) is

(57) $$ds^{2}=dt^{2}-\exp\left(2Ht\right)dx^{2}.$$

The “Euclidean” version of the metric (57) (x→ix)

(58) $$ds^{2}=dt^{2}+\exp\left(2Ht\right)dx^{2}$$

does not represent a metric on the sphere $S^{4}$. This is the metric on the hyperbolic space which is the Euclidean version of the anti-de Sitter space-time. In fact, the metric (58) can be expressed in a form familiar from the realization of the anti-de Sitter space as a generalized Poincare upper half-plane

$$ds^{2}=y^{-2}\left(H^{-2}dy^{2}+dx^{2}\right)$$

with y=exp(−Ht). The sphere $S^{4}$, Euclidean anti-de Sitter space and Euclidean continuations of de Sitter space are closely related [46]. We are allowed to treat the coordinates *t* and τ in Equations (51)–(58) in Lorentzian and Euclidean metrics as time in the Lagrangian formalism (in the Euclidean version, the time evolution will be a rotation of the sphere).

There remains to study the Schrödinger Equations (25)–(27) resulting from the definition of H in Equations (24) and (27). First, we look for a Gaussian solution (40) of these equations. In the coordinates (51)–(56), we can use the O(4) symmetry in order to diagonalize the equation for Γ. First, we expand the fields in the spherical harmonics [47]

$$\Phi \left(\tau ,\omega \right)=\sum_{lm}Y_{lm}\left(\omega \right)\Phi_{lm}\left(\tau \right),$$

where

$${▵}_{S}Y_{lm}=-l\left(l+2\right)Y_{lm}.$$

${▵}_{S}$ is the Laplace–Beltrami operator on $S^{3}$, *l* is a natural number, and m=(j,σ) where j=0,1,…,l and −j≤σ≤j, is the indexing solution of the Laplace–Beltrami operator with the eigenvalue −l(l+2) [48].

Then, we expand Γ as

(59) $$\Gamma_{\tau }\left(\omega ,\omega^{\prime }\right)=\sum_{lm}Y_{lm}\left(\omega^{\prime }\right)Y_{lm}^{*}\left(\omega \right)\Gamma_{lm}\left(\tau \right).$$

Using Formula (24), we can define the Hamiltonian in each of the metrics (51)–(58). Subsequently, solving Equation (25), we find a Gaussian solution (40) of the Schrödinger equation in each of these coordinates. We obtain for the metric (53) the Hamiltonian (24)

(60) $$\begin{array}{c} H=\sum_{lm}\left({\left(H\cos\left(\tau \right)\right)}^{2}\Pi_{lm}^{2}+{\left(H\cos\left(\tau \right)\right)}^{-2}l\left(l+2\right)\Phi_{lm}^{2}+M^{2}{\left(H\cos\left(\tau \right)\right)}^{-4}\Phi_{lm}^{2}\right) \end{array}$$

defining for t≥0 the Schrödinger Equation (25).

The Hamiltonian for the Euclidean metric (56) in the interpretation (27) is analogous to the upside-down oscillator [36,42,43] (we change $M^{2}\to -\mu^{2}$; the Schrödinger equation is still $i\hbar \partial_{\tau }\psi =H\psi$)

(61) $$\begin{array}{c} H=\sum_{lm}\left({\left(H\cosh\left(\tau \right)\right)}^{2}\Pi_{lm}^{2}-{\left(H\cosh\left(\tau \right)\right)}^{-2}l\left(l+2\right)\Phi_{lm}^{2}-\mu^{2}{\left(H\cosh\left(\tau \right)\right)}^{-4}\Phi_{lm}^{2}\right) \end{array}$$

We still consider the Schrödinger Equation (26). In the Euclidean metric (56) with the Hamiltonian (24) and the choice $\sqrt{-g}=-i\sqrt{\left|g\right|}$, we obtain the diffusion Equation (26) for τ<0 with

(62) $$\begin{array}{c} H_{E}=\sum_{lm}\left({\left(H\cosh\left(\tau \right)\right)}^{2}\Pi_{lm}^{2}+{\left(H\cosh\left(\tau \right)\right)}^{-2}l\left(l+2\right)\Phi_{lm}^{2}+\mu^{2}{\left(H\cosh\left(\tau \right)\right)}^{-4}\Phi_{lm}^{2}\right) \end{array}$$

so that the Schrödinger equation takes the form

$$\hbar \partial_{t}\psi =H_{E}\psi .$$

The operators Γ (28) and G (32) are diagonalized by an expansion is spherical functions. We denote a function satisfying the wave Equation (34) for G by $u_{\tau }\left(\omega \right)$. Expanding $u_{\tau }\left(\omega \right)$

$$u\left(\tau ,\omega \right)=\sum_{lm}Y_{lm}\left(\omega \right)u_{lm}\left(\tau \right)$$

we obtain that in the metric (53) the coefficients $u_{lm}$ satisfy the equation

(63) $$\partial_{\tau }^{2}u_{lm}+2\tan\left(\tau \right)\partial_{\tau }u_{lm}+\left(l\left(l+2\right)+M^{2}{\left(H\cos\left(\tau \right)\right)}^{-2}\right)u_{lm}=0.$$

Γ is related to *u*, as follows from Equation (32)

(64) $$u=\exp\left(\int^{\tau }ds{\left(H\cos\left(s\right)\right)}^{2}\Gamma \left(s\right)ds\right).$$

For the Euclidean metric with $\sqrt{-g}\to \sqrt{\left|g\right|}$, the corresponding formulas read (this equation also follows from the Hamiltonian (61))

(65) $$\begin{array}{c} \partial_{\tau }^{2}u_{lm}^{E}-2\tanh\left(\tau \right)\partial_{\tau }u_{lm}^{E}-l\left(l+2\right)u_{lm}^{E}-\mu^{2}{(H\cosh\left(\tau \right)}^{-2}u_{lm}^{E}=0 \end{array}$$

Note that the definition $\sqrt{-g}\to -i\sqrt{\left|g\right|}$ does not change the “wave equation” (65) for the inverted signature. With the inverted signature, the “wave equation” becomes an elliptic equation, hence it does not describe a wave propagation anymore.

$\Gamma^{E}$ is related to $u^{E}$ by

(66) $$u^{E}=\exp\left(i\int^{\tau }ds{\left(H\cosh\left(s\right)\right)}^{2}\Gamma^{E}\left(s\right)ds\right).$$

Concerning the flat expanding metric of the general form

(67) $$ds^{2}=g_{\mu \nu }dx^{\mu }dx^{\nu }\equiv dt^{2}-a^{2}\left(t\right)dx^{2}$$

introduced first for de Sitter in Equation (57) (and further discussed in models of subsequent sections) we note that Friedmann equations governing the evolution of a(t)

$${\left(a^{-1}\frac{d}{dt}a\right)}^{2}-\frac{1}{3}\Lambda =\frac{8\pi G}{3}\rho ,$$

$$2a^{-1}\frac{d^{2}a}{dt^{2}}+{\left(a^{-1}\frac{d}{dt}a\right)}^{2}-\Lambda =-8\pi Gp$$

are invariant under $a^{2}\to -a^{2}$. Here, Λ is the cosmological constant ($H=\sqrt{\frac{\Lambda }{3}}$), ρ is the energy density and *p* is the pressure. This transformation can equivalently be treated as a→ia. The physical interpretation forces us to choose as a solution the metric with $a^{2}>0$ (such a requirement may be not applicable in quantum gravity).

For the homogeneous metric (67), owing to the translation invariance, we can decompose Γ in Fourier components

$$\Gamma \left(x-y\right)={\left(2\pi \right)}^{-3}\int dk\Gamma \left(k\right)\exp\left(ik\left(x-y\right)\right).$$

We consider solutions satisfying the condition Γ(k)=Γ(−k)=Γ(k), where k=|k|. Then, in Fourier transform Equation (28) in an expanding metric is

$$\partial_{t}\Gamma +a^{-3}\Gamma^{2}+ak^{2}+M^{2}a^{3}=0.$$

We Fourier transform the wave function

$$u\left(x\right)={\left(2\pi \right)}^{-\frac{3}{2}}\int dk\exp\left(-i\mathrm{kx}\right)u_{k}.$$

Then, the wave Equation (34) is

(68) $$\partial_{t}^{2}u_{k}+3a^{-1}\partial_{t}a\partial_{t}u_{k}+a^{-2}k^{2}u_{k}+M^{2}u_{k}=0.$$

The relation between *u* and Γ is determined by Equation (32)

(69) $$u_{k}=\exp\left(\int^{t}dsa^{-3}\Gamma_{s}\left(k\right)\right)$$

When $a^{-2}<0$ for t<0, we chose $\sqrt{-g}=-i\sqrt{\left|g\right|}=-i{\left|a^{2}\right|}^{\frac{3}{2}}$. The Schrödinger equation for t>0 is

(70) $$\begin{array}{c} i\hbar \partial_{t}\psi_{t}=\frac{1}{2}\int dx\left(-\hbar^{2}a^{-3}\frac{\delta^{2}}{\delta \Phi {\left(x\right)}^{2}}+a{\left(\nabla \Phi \right)}^{2}+M^{2}a^{3}\Phi^{2}\right)\psi_{t} \end{array}$$

whereas for negative time, when $a^{2}<0$ and $\sqrt{-g}$ is imaginary, we have the diffusion Equation (26) (as discussed in Section 10)

(71) $$\begin{array}{c} \hbar \partial_{t}\psi_{t}=\frac{1}{2}\int dx\left(-\hbar^{2}|a^{2}{|}^{-\frac{3}{2}}\frac{\delta^{2}}{\delta \Phi {\left(x\right)}^{2}}+\sqrt{|a^{2}|}{\left(\nabla \Phi \right)}^{2}+\mu^{2}{\left|a^{2}\right|}^{\frac{3}{2}}\Phi^{2}\right)\psi_{t} \end{array}$$

where, for negative time, we changed the notation $M^{2}=-\mu^{2}$, suggesting that $M^{2}$ should be chosen to be negative.

If in the Lagrangian (22) $\sqrt{-g}\to \sqrt{\left|g\right|}$, then the Schrödinger equation reads

(72) $$\begin{array}{c} i\hbar \partial_{t}\psi_{t}=\frac{1}{2}\int dx\left(-\hbar^{2}\left(\right|a^{2}{\left|\right)}^{-\frac{3}{2}}\frac{\delta^{2}}{\delta \Phi {\left(x\right)}^{2}}+a^{-2}\left(\right|a^{2}{\left|\right)}^{\frac{3}{2}}{\left(\nabla \Phi \right)}^{2}-\mu^{2}\left(\right|a^{2}{\left|\right)}^{\frac{3}{2}}\Phi^{2}\right)\psi_{t} \end{array}$$

Equation (72) is an analog of the one for an inverted oscillator (see [36], so that the evolution is still unitary).

We note that from Equation (68) that it follows that the Wronskian is a constant as

$$\partial_{t}\left(a^{3}\left(u\partial_{t}u^{*}-u^{*}\partial_{t}u\right)\right)=0$$

For complex solutions, we choose the normalization

(73) $$u\partial_{t}u^{*}-u^{*}\partial_{t}u=-ia^{-3}.$$

which is fixing the constant in canonical commutation relations. If a=exp(Ht), as in Equation (57) then the (complex) solution of Equation (68) is the cylinder function $Z_{\nu }$ [49,50,51,52]

(74) $$u=a^{-\frac{3}{2}}Z_{\nu }\left(\frac{k}{H}\exp\left(-Ht\right)\right),$$

where

$$\nu =\frac{3}{2}\sqrt{1-\frac{4M^{2}}{9H^{2}}}.$$

In general, as solutions of the wave equation we can take superpositions of solutions with different *k*. The canonical quantization is realized with the requirement [50] that, in the remote past $t_{0}\to -\infty$, the solution tends to the plane wave (in conformal coordinates). Then, $Z_{\nu }=H_{\nu }^{\left(2\right)}$, where $H_{\nu }^{\left(2\right)}$ is the Hankel function of the second kind [49]. If M=0, then we can obtain real solutions of Equation (68) $J_{\frac{3}{2}}\left(\frac{k}{H}\exp\left(-Ht\right)\right)$ and $Y_{\frac{3}{2}}\left(\frac{k}{H}\exp\left(-Ht\right)\right)$, important for the construction of interactions in Section 11 and Section 12. These real solutions will give oscillatory evolution kernels with caustic singularities.

The solution of Equation (68) with an inverted metric (k→ik and M→iμ in Equation (68)) is

$$u=a^{-\frac{3}{2}}J_{\nu }\left(i\frac{k}{H}\exp\left(-Ht\right)\right)=Ca^{-\frac{3}{2}}K_{\nu }\left(\frac{k}{H}\exp\left(-Ht\right)\right)$$

with a certain constant *C*, and

$$\nu =\frac{3}{2}\sqrt{1+\frac{4\mu^{2}}{9H^{2}}}$$

It can be seen that this is a solution of the Euclidean “wave equation”, corresponding to the field theory on the Euclidean version of the anti-de Sitter space [46,53].

Summarizing, the aim of this section was to reduce the general Equations (28)–(34) to a manageable form using the symmetry of the manifold. In the homogeneous expanding coordinates, we can use the Fourier transform to represent the operator Γ as a multiplication operator in the Fourier space. In angular coordinates (covering the whole of de Sitter space), we can expand the solution in terms of spherical harmonics. A change in the spatial signature in angular coordinates transforms de Sitter space into a sphere. The Hamiltonian and the solution of the Schrödinger equation are expressed in terms of a discrete set of variables. The quantization of these variables (as outlined in Section 2, Section 3 and Section 4) is achieved by stochastic equations in the next section.

## 6. Stochastic Equations for de Sitter Field and Fields in an Expanding Flat Metric

In this and in subsequent sections, we discuss the stochastic time evolution for positive, negative and imaginary time. Until now, only positive time was considered in the stochastic representation (45), because the Brownian motion is defined for a positive time. We can obtain a stochastic representation for a negative time, taking the complex conjugation of Equation (25)

(75) $$i\hbar \partial_{-t}\psi_{t}^{*}=H^{*}\psi_{t}^{*}\equiv \tilde{H}\left(-t\right)\psi_{t}^{*}.$$

If the Schrödinger Equation (25) is to be defined for positive and negative time, then the expressions for the Hamiltonian as a function of the metric tensor must have a meaning in this range of time. This may be not possible if Hawking–Penrose positivity conditions of the energy-momentum are to be satisfied [32] (then the metric tensor may become degenerate and $\frac{1}{\sqrt{-g}}$ infinite). The Einstein equations are invariant under the time reflection. However, the reflected metric can violate the requirement of −g>0, as will be discussed in Section 10. In such a case, $\tilde{H}\left(-t\right)$ for t>0 in the interpretation (24) does not define a Hermitian operator. In fact, in Section 10, H(−t) will be anti-Hermitian. In such a case, the Schrödinger equation for a positive time is transformed into a diffusion equation for a negative time.

In general, on a globally hyperbolic manifold, if we find $\Gamma_{t}$ from Equation (28), then the stochastic equation generated by the Hamiltonian (24) reads (where J is defined in Equation (30))

(76) $$d\Phi_{s}=-J\left(t-s\right)\Gamma_{t-s}\Phi_{s}ds+\sqrt{i\hbar }\sqrt{g_{00}}{\left(-g\right)}^{-\frac{1}{4}}\left(t-s\right)dW_{s}.$$

For a negative time, Equation (76) reads

(77) $$d\Phi_{s}=J\left(t-s\right)\Gamma_{t-s}\Phi_{s}d\left(-s\right)+\sqrt{-i\hbar }\sqrt{g_{00}}{\left(-g\right)}^{-\frac{1}{4}}\left(t-s\right)dW_{-s}.$$

With $\sqrt{-g}=-i\sqrt{\left|g\right|}$ and ${\left(-g\right)}^{\frac{1}{4}}=\sqrt{-i}{\left|g\right|}^{\frac{1}{4}}$ in Equation (77) for −g<0. Such a choice of square roots will give the real noise ($\sqrt{-i}$ cancels) and a positive operator $H^{E}$ in the diffusion Equation (26).

Equation (77) can be rewritten as

(78) $$d\Phi_{s}=G^{-1}\partial_{t}G\left(t-s\right)\Phi_{s}d\left(-s\right)+\sqrt{\hbar }\sqrt{g_{00}}{\left|g\right|}^{-\frac{1}{4}}\left(t-s\right)dW_{-s}.$$

where G is the solution of the wave Equation (34) (with an inverted metric). Equation (76) is a generalization of Equation (44) (considered in the Minkowski space-time).

In the expanding metric (67), the Hamiltonian (24) for t>0 is

(79) $$H=\frac{1}{2}\int dx\left(-\hbar^{2}a^{-3}\frac{\delta^{2}}{\delta \Phi {\left(x\right)}^{2}}+a{\left(\nabla \Phi \right)}^{2}+M^{2}a^{3}\Phi^{2}\right).$$

The stochastic Equation (76) for t≥0 reads

(80) $$d\Phi_{s}=-a{\left(t-s\right)}^{-3}\Gamma \left(t-s\right)\Phi_{s}ds+a{\left(t-s\right)}^{-\frac{3}{2}}\sqrt{i\hbar }dW_{s}.$$

By a differentiation of Equation (80), we obtain a random wave equation

(81) $$\begin{array}{c} \left(\partial_{s}^{2}-a^{-2}\left(t-s\right)▵+M^{2}\right)\Phi_{s}+3a^{-1}\left(t-s\right)\partial_{t}a\left(t-s\right)\partial_{s}\Phi_{s}\\ =\left(\frac{9}{2}a^{-1}\left(t-s\right)\partial_{t}a\left(t-s\right)-a^{-3}\left(t-s\right)\Gamma \left(t-s\right)\right)\sqrt{i\hbar }\partial_{s}W\\ +\sqrt{i\hbar }a^{-\frac{3}{2}}\left(t-s\right)\partial_{s}^{2}W. \end{array}$$

Equation (80) has the solution (with the initial condition Φ at s=0)

(82) $$\Phi_{s}=u_{t-s}u_{t}^{-1}\Phi +\sqrt{i\hbar }u_{t-s}\int_{0}^{s}u_{t-\tau }^{-1}a_{t-\tau }^{-\frac{3}{2}}dW_{\tau }.$$

Equation (80) can be interpreted in the semi-classical approximation if in the solution (3.20) Γ is real (so *u* is real). Then, in the limit ℏ→0, Equation (80) reads

(83) $$\frac{d\Phi_{s}}{ds}=-a^{-3}\left(t-s\right)\Gamma \left(t-s\right)\Phi_{s}.$$

Equation (83) relates the Hamilton–Jacobi limit of the Schrödinger equation with its classical solution $\Phi_{s}$. In the standard formulation of the Hamilton–Jacobi theory, if *S* is the classical action, then the classical trajectory is defined by [44]

$$a^{-3}\frac{d\Phi_{s}}{ds}=\frac{\delta S}{\delta \Phi_{s}}.$$

From Equation (82), the classical solution with the initial condition Φ is

$$\Phi_{s}=u_{t-s}u_{t}^{-1}\Phi ,$$

where $u_{t-s}$ is the classical solution of the wave equation resulting from the Lagrangian (22).

For the negative time, the stochastic evolution is a simple reflection if a(t)=a(−t), as appears in the effective field theories resulting from the string theory [54,55]. Then, we have $\tilde{H}\left(-t\right)=H\left(t\right)$, and *a* is contracting to zero as |t|→0 and expanding to infinity when t→∞. In such a case, the stochastic Equation (77) takes the form

$$d\Phi_{s}=\Gamma \left(s-t\right)a^{-3}\left(t-s\right)\Phi_{s}d\left(-s\right)+\sqrt{-i\hbar }a^{-\frac{3}{2}}\left(t-s\right)dW_{-s},$$

where $a^{-3}\Gamma =u^{-1}\partial_{t}u$. It has the solution

(84) $$\Phi_{s}\left(\Phi \right)=u_{t-s}^{\left(-\right)}{\left(u_{t}^{\left(-\right)}\right)}^{-1}-\sqrt{-i\hbar }u_{t-s}^{\left(-\right)}\int_{0}^{-s}{\left(u_{t+\tau }^{\left(-\right)}\right)}^{-1}a_{t+\tau }^{-\frac{3}{2}}dW_{\tau },$$

where $u_{t}^{\left(-\right)}$ is the solution of the wave Equation (68) for a negative time. In this case, the field $\Phi_{s}$ can be considered as a simple reflection of the one for s>0. We discuss an example of a(t)≃|t| in Section 10.

We return to de Sitter expanding metric (57). From Equation (69),

$$\Gamma =a^{3}\frac{d}{dt}\ln\left(a^{-\frac{3}{2}}Z_{\nu }\left(\frac{k}{H}\exp\left(-Ht\right)\right)\right).$$

Hence,

$$\begin{array}{c} \Phi_{s}=\exp\left(-\int_{t_{0}}^{s}\left(a^{-3}\Gamma \right)\left(t-\tau \right)d\tau \right)\Phi +\sqrt{i\hbar }\int_{t_{0}}^{s}\exp\left(-\int_{\tau }^{s}\left(a^{-3}\Gamma \right)\left(t-\tau^{\prime }\right)d\tau^{\prime }\right)a{\left(t-\tau \right)}^{-\frac{3}{2}}dW_{\tau } \end{array}$$

or

(85) $$\begin{array}{c} \Phi_{s}=a{\left(t-s\right)}^{-\frac{3}{2}}Z_{\nu }\left(\frac{k}{H}\exp\left(-Ht+Hs\right)\right)a{\left(t-t_{0}\right)}^{\frac{3}{2}}\\ {\left(Z_{\nu }\left(\frac{k}{H}\exp\left(-Ht+Ht_{0}\right)\right)\right)}^{-1}\Phi +\sqrt{i\hbar }a{\left(t-s\right)}^{-\frac{3}{2}}Z_{\nu }\left(\frac{k}{H}\exp\left(-Ht+Hs\right)\right)\\ \int_{t_{0}}^{s}{\left(Z_{\nu }\left(\frac{k}{H}\exp\left(-Ht+H\tau \right)\right)\right)}^{-1}dW_{\tau }. \end{array}$$

The solution of the stochastic equation determines the field correlation functions (from Equations (47) and (85), $\hat{\Phi }$ denotes the quantum field, we set $t_{0}=0$)

(86) $$\begin{array}{c} \left(\psi_{0}^{g},{\hat{\Phi }}_{t}\left(k\right)\hat{\Phi }\left(k^{\prime }\right)\psi_{0}^{g}\right)=\int d\Phi {\left|\psi_{t}^{g}\left(\Phi \right)\right|}^{2}\Phi \left(k\right)E\left[\Phi_{t}\left(\Phi ,k^{\prime }\right)\right]\\ =i{\left(\Gamma \left(t\right)-\Gamma {\left(t\right)}^{*}\right)}^{-1}Z_{\nu }\left(\frac{k}{H}\right)a{\left(t\right)}^{\frac{3}{2}}{\left(Z_{\nu }\left(\frac{k}{H}\exp\left(-Ht\right)\right)\right)}^{-1}\delta \left(k+k^{\prime }\right), \end{array}$$

where we have used the covariance (from Equation (40))

(87) $$\int d\Phi |\psi_{t}^{g}{\left(\Phi \right)|}^{2}\Phi \left(k\right)\Phi \left(k^{\prime }\right)=i\hbar {\left(\Gamma -\Gamma^{*}\right)}^{-1}\delta \left(k+k^{\prime }\right).$$

In general,

$$Z_{\nu }=\beta H_{\nu }^{\left(1\right)}+\alpha H_{\nu }^{\left(2\right)},$$

where $H_{\nu }$ are the Hankel functions (in order to satisfy canonical commutation relations we must have ${\left|\alpha \right|}^{2}-{\left|\beta \right|}^{2}=1$).

In order to calculate the rhs of Equation (87) we apply (with $z=\frac{k}{H}\exp\left(-Ht\right)$)

(88) $$\begin{array}{c} \Gamma \left(t\right)-\Gamma {\left(t\right)}^{*}=a^{3}Hz{\left(Z_{\nu }^{*}Z_{\nu }\right)}^{-1}\left(\frac{d}{dz}Z_{\nu }^{*}Z_{\nu }-\frac{d}{dz}Z_{\nu }Z_{\nu }^{*}\right). \end{array}$$

The rhs of the expression (87) may have any sign. For β=0 and α=1 (the Bunch–Davies vacuum [52]), we have

(89) $$\Gamma -\Gamma^{*}=i{\left(H_{\nu }^{(2)*}H_{\nu }^{\left(2\right)}\right)}^{-1}$$

Then, inserting Equation (89) in Equation (86), we obtain

(90) $$\begin{array}{c} \left(\psi_{0}^{g},{\hat{\Phi }}_{t}\left(k\right)\hat{\Phi }\left(k^{\prime }\right)\psi_{0}^{g}\right)=\hbar H_{\nu }^{\left(2\right)}\left(\frac{k}{H}\right)a{\left(t\right)}^{\frac{3}{2}}{\left(H_{\nu }^{\left(2\right)}(\frac{k}{H}\exp\left(-Ht\right)\right)}^{*}\delta \left(k+k^{\prime }\right). \end{array}$$

From Equation (47), using Equation (90), we can also calculate (for the massless field)

$$\begin{array}{c} \left(\psi_{0}^{g},{\hat{\Phi }}_{t}\left(k\right){\hat{\Phi }}_{t}\left(k^{\prime }\right)\psi_{0}^{g}\right)=\left(\psi_{t}^{g},\Phi \left(k\right)\Phi \left(k^{\prime }\right)\psi_{t}^{g}\right)\\ =\hbar \delta \left(k+k^{\prime }\right)H_{\frac{3}{2}}^{(2)*}H_{\frac{3}{2}}^{\left(2\right)}\left(\frac{k}{H}\exp\left(-Ht\right)\right) \end{array}$$

and

$$\begin{array}{c} \left(\psi_{0}^{g},\Phi \left(k\right)\Phi \left(k^{\prime }\right)\psi_{0}^{g}\right)-\left(\psi_{0}^{g},{\hat{\Phi }}_{t}\left(k\right){\hat{\Phi }}_{t}\left(k^{\prime }\right)\psi_{0}^{g}\right)\\ =\hbar \delta \left(k+k^{\prime }\right)\frac{1}{2k}\left(1-\exp\left(-2Ht\right)\right) \end{array}$$

a result obtained in [56,57,58,59].

In a similar way, using Equation (47), we calculate higher order correlation functions

$$\begin{array}{c} \left(\psi_{0}^{g},{\hat{\Phi }}_{t}\left(k_{1}\right){\hat{\Phi }}_{t}\left(k_{2}\right)\hat{\Phi }\left(k_{3}^{\prime }\right)\hat{\Phi }\left(k_{4}^{\prime }\right)\psi_{0}^{g}\right)\\ =\int d\Phi |\psi_{t}^{g}{\left(\Phi \right)|}^{2}\Phi \left(k_{1}\right)\Phi \left(k_{2}\right)E\left[\Phi_{t}\left(\Phi ,k_{3}^{\prime }\right)\Phi_{t}\left(\Phi ,k_{4}^{\prime }\right)\right] \end{array}$$

In this equation, we insert the solution (82). The integral over Φ is the Gaussian integral with the covariance (87). The expectation value in the formula for the correlations is

(91) $$\begin{array}{c} E\left[\Phi_{t}\left(\Phi ,k_{3}^{\prime }\right)\Phi_{t}\left(\Phi ,k_{4}^{\prime }\right)\right]\\ =\left(u_{0}\Phi \right)\left(k_{3}^{\prime }\right){\left(\left(u_{t}\Phi \right)\left(k_{3}^{\prime }\right)\right)}^{-1}\left(u_{0}\Phi \right)\left(k_{4}^{\prime }\right){\left(\left(u_{t}\Phi \right)\left(k_{4}^{\prime }\right)\right)}^{-1}\\ +i\hbar u_{0}^{2}\delta \left(k_{3}^{\prime }+k_{4}^{\prime }\right)\int_{0}^{t}u_{\tau }^{-2}\left(k_{3}^{\prime }\right)a_{\tau }^{-3}d\tau \equiv E_{cl}+G_{t}, \end{array}$$

where the $G_{t}$ term (it still will be discussed in subsequent sections) being the quantum fluctuation of $\Phi_{t}$ is proportional to *ℏ*. The integral (91) can explicitly be calculated for M=0. Then, inserting $H_{\frac{3}{2}}^{\left(2\right)}$

$$a_{\tau }^{\frac{3}{2}}u_{\tau }\left(k\right)=Cz^{-\frac{3}{2}}\left(z-i\right)\exp\left(-iz\right)$$

with a certain constant *C* and $z\left(\tau \right)=\frac{k}{H}\exp\left(-H\tau \right)$, we obtain the integral in Equation (91). The integral reads

$$\begin{array}{c} G_{t}=-i\hbar {\left(\frac{k}{H}-i\right)}^{2}\frac{H^{2}}{k^{3}}\exp\left(-2i\frac{k}{H}\right)\\ \int_{\frac{k}{H}}^{\frac{k}{H}\exp\left(-Ht\right)}y^{2}{\left(y-i\right)}^{-2}\exp\left(2iy\right)dy. \end{array}$$

For a small *k*, the leading infrared behavior of the real part of $G_{t}$ is $\frac{Ht}{k}$ confirming the diffusive behavior of $\Phi_{t}$ discovered in [56,57,58,59].

In the case of an inverted metric (58) describing the Euclidean anti-de Sitter space the correlation function is an analytic continuation of Equation (90)

(92) $$\begin{array}{c} H_{\nu }^{\left(2\right)}\left(i\frac{k}{H}\right)a{\left(t\right)}^{-\frac{3}{2}}{\left(H_{\nu }^{\left(2\right)}(i\frac{k}{H}\exp\left(-Ht\right)\right)}^{*}\delta \left(k+k^{\prime }\right)\\ ≃K_{\nu }\left(\frac{k}{H}\right)a{\left(t\right)}^{-\frac{3}{2}}K_{\nu }(\frac{k}{H}\exp\left(-Ht\right))\delta \left(k+k^{\prime }\right) \end{array}$$

with $\nu =\frac{3}{2}\sqrt{1+\frac{4\mu^{2}}{9H^{2}}}$. The correlation function (90) coincides with the one derived in [50,51,52] describing the quantum free field in de Sitter space, with the ground state invariant under the de Sitter group. The two-point function (92) describes the Euclidean version of the quantum field on the anti-de Sitter space [6,46].

With the solution (85)–(90), we shall have the same problem with a perurbative construction of an interaction (ultraviolet divergencies and renormalization) as in the QFT in the Minkowski space-time. In Section 11 and Section 12, we discuss a method to construct interactions when the solution of the wave equation u(t) is a real function, and we do not insist on the existence of the ground state. In the derivation of Equation (90), we have chosen as *u* the Hankel function $H_{\nu }^{\left(2\right)}\left(\frac{k}{H}\exp\left(-Ht\right)\right)$. In such a case, Γ is complex. Hence, $\psi_{t}^{g}$ is not a pure phase WKB solution. If M=0, then $\nu =\frac{3}{2}$. We could chose as *u* the Bessel functions $J_{\frac{3}{2}}$ or $Y_{\frac{3}{2}}$, which are expressed by trigonometric functions. In such a case, Γ is real and $\psi_{t}^{g}$ is a pure phase. We obtain another representation of de Sitter field with caustic singularities, which will be discussed in another model in Section 10. The Bessel functions of an imaginary argument $K_{\nu }$ in Equation (92) give a solution in Euclidean AdS. In such a case, $u_{\nu }$ is a real function without caustic poles. We can construct interaction without an ultraviolet cutoff by means of the Feynman–Kac formula, as discussed in Section 12.

Let us still determine the stochastic field (76) resulting from the Hamiltonian (60). It is the solution of the stochastic equation

(93) $$d\Phi_{lm}\left(s\right)=-\partial_{\tau }\ln\left(u_{lm}\left(\tau -s\right)\right)\Phi_{lm}\left(s\right)ds+\sqrt{i\hbar }H^{\frac{3}{2}}\cos\left(\tau -s\right)dw_{lm}\left(s\right),$$

where $u_{lm}$ is the solution of Equation (63) and $w_{lm}$ are Gaussian processes with mean zero and the covariance

(94) $$E\left[w_{lm}\left(s\right)w_{l^{\prime }m^{\prime }}\left(s^{\prime }\right)\right]=\delta_{ll^{\prime }}\delta \left(m+m^{\prime }\right)min\left(s,s^{\prime }\right),$$

where m=(j,σ) (as explained at Equation (59)) and $\delta \left(m+m^{\prime }\right)=\delta \left(j-j^{\prime }\right)\delta \left(\sigma +\sigma^{\prime }\right)$.

The Euclidean Hamiltonian (62) generates the stochastic equation

(95) $$\begin{array}{c} d\Phi_{lm}{\left(s\right)}^{E}=-\partial_{\tau }\ln\left(u_{lm}^{E}\left(\tau -s\right)\right)\Phi_{lm}^{E}\left(s\right)d\left(-s\right)+\sqrt{\hbar }H^{\frac{3}{2}}\cosh\left(\tau -s\right)dw_{lm}\left(-s\right), \end{array}$$

where $u_{lm}^{E}$ is the solution of Equation (65).

The solution defines a real diffusion process solving the diffusion Equation (26) for τ≤0.

The solution of Equation (93) is

(96) $$\begin{array}{c} \Phi_{lm}\left(s\right)=u_{lm}\left(\tau -s\right)u_{lm}{\left(\tau \right)}^{-1}\Phi_{lm}+\sqrt{i\hbar }H^{\frac{3}{2}}u_{lm}\left(\tau -s\right)\int_{0}^{s}{\left(u_{lm}\left(\tau -t\right)\right)}^{-1}\cos\left(\tau -t\right)dw_{lm}\left(t\right). \end{array}$$

Equation (95) has the solution

(97) $$\begin{array}{c} \Phi_{lm}^{E}\left(s\right)=u_{lm}^{E}\left(\tau -s\right)u_{lm}^{E}{\left(\tau \right)}^{-1}\Phi_{lm}+\sqrt{\hbar }u_{lm}^{E}\left(\tau -s\right)H^{\frac{3}{2}}\int_{0}^{s}{\left(u_{lm}^{E}\left(\tau -t\right)\right)}^{-1}\cosh\left(\tau -t\right)dw_{lm}\left(t\right), \end{array}$$

where $\Phi_{lm}$ is the initial condition at s=0. We need to calculate

$$\begin{array}{c} E\left[\left(\Phi_{lm}\left(s\right)-E\left[\Phi_{lm}\left(s\right)\right]\right)\left(\Phi_{lm}\left(s^{\prime }\right)-E\left[\Phi_{l^{\prime }m^{\prime }}\left(s^{\prime }\right)\right]\right)\right]=G_{lm}\left(s,s^{\prime }\right)\delta_{ll^{\prime }}\delta \left(m+m^{\prime }\right). \end{array}$$

We have

(98) $$\begin{array}{c} G_{lm}\left(s,s^{\prime }\right)=i\hbar u_{lm}\left(\tau -s\right)u_{lm}\left(\tau -s^{\prime }\right)H^{3}\int_{0}^{m(s,s^{\prime })}dt{\left(u_{lm}\left(\tau -t\right)\right)}^{-2}\cos^{2}\left(\tau -t\right) \end{array}$$

for the field (96) and

(99) $$\begin{array}{c} G_{lm}^{E}\left(s,s^{\prime }\right)=\hbar u_{lm}^{E}\left(\tau -s\right)u_{lm}^{E}\left(\tau -s^{\prime }\right)H^{3}\int_{0}^{m(s,s^{\prime })}dt{\left(u_{lm}^{E}\left(\tau +t\right)\right)}^{-2}\cosh^{2}\left(\tau -t\right) \end{array}$$

for the inverted metric of Equation (56).

For a general *l* and *M*, the solution of the wave equation is defined by the Legendre functions [50]. We are unable to calculate the integrals in $G_{lm}\left(s,s^{\prime }\right)$ (98) and (99) exactly. For l=0 and M=0, we have

(100) $$u_{00}\left(\tau \right)=\tau +\frac{1}{4}\sin\left(2\tau \right)$$

and

$$u_{00}^{E}\left(\tau \right)=\tau +\frac{1}{4}\sinh\left(2\tau \right).$$

For a large *l*, we can obtain the WKB solution (the odd solution) of the wave equation

(101) $$u_{lm}\left(\tau \right)={\left(\frac{dS}{d\tau }\right)}^{-\frac{1}{2}}\sin\left(S\left(\tau \right)\right)\cos\left(\tau \right),$$

where

$$S\left(s\right)=\int_{0}^{s}d\tau \sqrt{l\left(l+2\right)+1+\left(M^{2}-2H^{2}\right)H^{-2}\cos^{-2}\left(\tau \right)}.$$

In the even solution, we replace sin in Equation (101) by cos. For the Euclidean Equation (65), the WKB solution is of the form (101), but the trigonometric functions are replaced by hyperbolic functions. So, the odd solution reads

(102) $$u_{lm}^{E}\left(\tau \right)={\left(\frac{dS^{E}}{d\tau }\right)}^{-\frac{1}{2}}\sinh\left(S^{E}\left(\tau \right)\right)\cosh\left(\tau \right)$$

with

(103) $$S^{E}\left(s\right)=\int_{0}^{s}d\tau \sqrt{l\left(l+2\right)+1+\left(\mu^{2}-2H^{2}\right)H^{-2}\cosh^{-2}\left(\tau \right)}.$$

For the Euclidean version, an exponential solution of Equation (65) will be needed (the sum of even and odd solutions)

(104) $$u_{lm}\left(\tau \right)={\left(\frac{dS^{E}}{d\tau }\right)}^{-\frac{1}{2}}\exp\left(S^{E}\left(\tau \right)\right)\cosh\left(\tau \right).$$

We can calculate $G_{lm}$ using the WKB approximation with the result

(105) $$\begin{array}{c} G_{lm}\left(s,s^{\prime }\right)=i\hbar H^{3}u_{lm}\left(\tau -s\right)u_{lm}\left(\tau -s^{\prime }\right)(\cot\left(S\left(\tau -m\left(s,s^{\prime }\right)\right)-\cot S\left(\tau \right)\right) \end{array}$$

for $u_{lm}$ defined by Equation (101) and

(106) $$\begin{array}{c} G_{lm}\left(s,s^{\prime }\right)=\hbar H^{3}u_{lm}^{E}\left(\tau -s\right)u_{lm}^{E}\left(\tau -s^{\prime }\right)(\coth\left(S^{E}\left(\tau -m\left(s,s^{\prime }\right)\right)-\coth S^{E}\left(\tau \right)\right) \end{array}$$

for the inverted metric in Equation (102).

In the case of the exponential solution (104), we obtain

(107) $$\begin{array}{c} G_{lm}\left(s,s^{\prime }\right)=\hbar H^{3}u_{lm}^{E}\left(\tau -s\right)u_{lm}^{E}\left(\tau -s^{\prime }\right)(\exp\left(-2S^{E}\left(\tau -m\left(s,s^{\prime }\right)\right)-\exp\left(-2S^{E}\left(\tau \right)\right)\right). \end{array}$$

For the even solutions of the WKB form defined by cos and cosh in Equations (101) and (102), the cotangent functions in Equations (105) and (106) are replaced by the tangents. The approximate expression for $G_{lm}\left(s,s^{\prime }\right)$ can be applied for calculations of the propagators and field correlations in subsequent sections. In Appendix F, we express fields in de Sitter space and their correlations in the cosmic time. There, we also discuss de Sitter fields in two dimensions (see earlier papers [60,61]), where we express the correlation functions by elementary functions.

With the trigonometric functions entering $G_{lm}\left(s,s^{\prime }\right)$, we have the difficulty with the integrability over time in the definition of the evolution kernel in Section 7 of quantum field theory in the Schrödinger formulation (because of the poles of the trigonometric functions in Equation (105)). We note that the replacement of the trigonometric functions by hyperbolic functions (signature inversion) in the approximate WKB solutions (106) avoids this difficulty. This analytic continuation allows a definition of the Feynman–Kac formula for a real time (but with an inverted spatial signature), as will be discussed in Section 11 and Section 12.

## 7. The Propagator for the Schrödinger Evolution of the Scalar Field

We can express the time evolution either by the solution of the stochastic equation as in Equation (45) or by an evolution kernel defined by

$$\left(U_{t}\psi \right)\left(\Phi \right)=\int d\Phi^{\prime }{\tilde{K}}_{t}\left(\Phi ,\Phi^{\prime }\right)\psi \left(\Phi^{\prime }\right).$$

We write ψ in the form (5). Then, the definition of the kernel is rewritten as

(108) $$\left(U_{t}\psi_{0}^{g}\chi \right)\left(\Phi \right)=\psi_{t}^{g}\int d\Phi^{\prime }K_{t}\left(\Phi ,\Phi^{\prime }\right)\chi \left(\Phi^{\prime }\right).$$

We represent χ as a Fourier transform

$$\chi \left(\Phi \right)=\int d\Lambda \tilde{\chi }\left(\Lambda \right)\exp\left(i\left(\Lambda ,\Phi \right)\right).$$

Then, from Equation (45), we obtain

(109) $$\begin{array}{c} K_{t}\left(\Phi ,\Phi^{\prime }\right)=\int d\Lambda E\left[\exp\left(i(\Lambda ,\Phi_{t}\left(\Phi \right)-\Phi^{\prime })\right)\right]. \end{array}$$

We calculate the expectation value of $\Phi_{t}$ with the result

(110) $$\begin{array}{c} E\left[\exp\left(i(\Lambda ,\Phi_{t}\left(\Phi \right)-\Phi^{\prime })\right)\right]=\exp\left(i\left(\Lambda ,u_{0}u_{t}^{-1}\Phi -\Phi^{\prime }\right)-\frac{1}{2}\left(\Lambda ,G_{t}\Lambda \right)\right). \end{array}$$

Then, calculating the Λ integral we obtain (up to a normalization constant)

(111) $$\begin{array}{c} K_{t}\left(\Phi ,\Phi^{\prime }\right)=\det G_{t}^{-\frac{1}{2}}\exp\left(-\frac{1}{2}\left(\left(u_{0}u_{t}^{-1}\Phi -\Phi^{\prime }\right),G_{t}^{-1}\left(u_{0}u_{t}^{-1}\Phi -\Phi^{\prime }\right)\right)\right], \end{array}$$

where

$$\begin{array}{c} G_{t}\left(k,k^{\prime }\right)=E\left[\left(\Phi_{t}\left(k\right)-<\Phi_{t}>\left(k\right)\right)\left(\Phi_{t}\left(k^{\prime }\right)-<\Phi_{t}>\left(k^{\prime }\right)\right)\right]\\ \equiv G_{t}\left(k\right)\delta \left(k+k^{\prime }\right) \end{array}$$

with <Φ>=E[Φ].

In a homogeneous expanding space-time for positive time (to be concise, from now on we omit the $\delta (k+k^{\prime })$ term in $G_{t}\left(k,k^{\prime }\right)$)

(112) $$G_{t}=i\hbar u_{0}^{2}\int_{0}^{t}u_{t-\tau }^{-2}a_{t-\tau }^{-3}d\tau .$$

Equation (112), (where $m\left(s,s^{\prime }\right)\equiv min\left(s,s^{\prime }\right)$, is a consequence of

$$E\left[\int_{0}^{s}f_{\tau }dW_{\tau }\left(k\right)\int_{0}^{s^{\prime }}f_{\tau^{\prime }}dW_{\tau^{\prime }}\left(k^{\prime }\right)\right]=\int_{0}^{m(s,s^{\prime })}f_{\tau }^{2}d\tau \delta \left(k+k^{\prime }\right)$$

following from the definition of the stochastic integral [38].

In the presence of an interaction from Equation (46), we obtain a generalization of the Formula (111) for the evolution kernel as

$$\begin{array}{c} K_{t}\left(\Phi ,\Phi^{\prime }\right)=\int d\Lambda E\left[\exp\left(i(\Lambda ,\Phi_{t}\left(\Phi \right)-\Phi^{\prime })\right)\exp\left(-\frac{i}{\hbar }\int_{0}^{t}V\left(\Phi_{s}\left(\Phi \right)\right)ds\right)\right]. \end{array}$$

Clearly, we cannot explicitly calculate this expectation value and the Λ integral as we did in Equations (110) and (111), but we can perform such calculations in a perturbation expansion in *V*, as will be discussed in Section 11 and Section 12.

For negative time in Equation (78), the correlation function is

(113) $$G_{t}=i\hbar u_{0}^{\left(-\right)}u_{0}^{\left(-\right)}\int_{0}^{-t}{\left(u_{t+\tau }^{\left(-\right)}\right)}^{-2}a_{t+\tau }^{-3}d\tau$$

where $u^{\left(-\right)}$ is the solution of the wave equation for a negative time. Let us note that, for positive time, we obtain an oscillating propagator $K_{t}$, which is a pure phase, whereas for an inverted metric (for the negative time), we obtain a real function describing a transition function for a diffusion.

The result of (111) and (112) gives an explicit formula for the evolution kernel. Another way to calculate this propagator involves a solution of the Cauchy problem for the wave equation, as discussed briefly at the end of Appendix D (such calculations are performed in more detail in [62]). Note that a real *u* leads to a purely imaginary $G_{t}$. Then, we obtain the propagator (111), which is a pure phase in agreement with the Feynman Formula (36) and Appendix D. If *u* is complex, then Γ is complex. Hence, $G_{t}$ is complex. Such a modification of the kernel results from its definition (108) involving $\psi_{t}^{g}$, which is not a pure phase.

Using the expectation value (110), we can also calculate the field correlation functions in a state $\psi_{0}^{g}\chi$ with a Gaussian state χ of the form

(114) $$\chi =\det B^{\frac{1}{2}}\exp\left(-\frac{1}{2}\left(\Phi ,B\Phi \right)\right).$$

Then, from Equation (110) (up to a constant multiplier)

(115) $$\begin{array}{c} \chi_{t}\left(\Phi \right)=\det B\int d\Lambda \exp\left(-\frac{1}{2}\left(\Lambda ,B^{-1}\Lambda \right)\right)\\ \exp\left(i\left(\Lambda ,u_{0}u_{t}^{-1}\Phi \right)-\frac{1}{2}\left(\Lambda ,G_{t}\Lambda \right)\right)=\det{\left(1+BG_{t}\right)}^{-\frac{1}{2}}\\ \det B^{\frac{1}{2}}\exp\left(-\frac{1}{2}\left(u_{0}u_{t}^{-1}\Phi ,{\left(B^{-1}+G_{t}\right)}^{-1}u_{0}u_{t}^{-1}\Phi \right)\right). \end{array}$$

Next,

(116) $$\begin{array}{c} {\left(\Phi \chi \right)}_{t}\left(\Phi \right)=u_{0}u_{t}^{-1}B^{-1}{\left(B^{-1}+G_{t}\right)}^{-1}\Phi \det B^{\frac{1}{2}}\det{\left(1+BG_{t}\right)}^{-\frac{1}{2}}\\ \exp\left(-\frac{1}{2}\left(u_{0}u_{t}^{-1}\Phi ,{\left(B^{-1}+G_{t}\right)}^{-1}u_{0}u_{t}^{-1}\Phi \right)\right). \end{array}$$

Then, the field correlation function according to Equation (48) can be calculated (for a general solution $\psi_{t}^{g}$ of the Schrödinger Equation (25)) from the formula

(117) $$\begin{array}{c} \left(\psi_{0}^{g}\chi ,{\hat{\Phi }}_{t}\left(k\right)\hat{\Phi }\left(k^{\prime }\right)\psi_{0}^{g}\chi \right)=\delta \left(k+k^{\prime }\right)\int d\Phi {\left|\psi_{t}^{g}\right|}^{2}\chi_{t}^{*}\left(\Phi \right)\Phi \left(k\right){\left(\Phi \chi \right)}_{t}\left(\Phi ,k^{\prime }\right), \end{array}$$

where $\chi_{t}$ and ${\left(\Phi \chi \right)}_{t}$ have been evaluated in Equations (115) and (116).

The integral (117) is Gaussian. Hence, we derive an explicit (although quite complicated) formula for the correlation function. The formula simplifies if, e.g., $|\psi_{t}^{g}{|}^{2}$ is integrable (when $i(\Gamma -\Gamma^{*})<0$) and χ=1 (as in Equation (91)). Then, ${\left(\Phi \chi \right)}_{t}\left(\Phi \right)=E\left[\Phi_{t}\left(\Phi \right)\right]=u_{0}u_{t}^{-1}\Phi$.

We can calculate the field correlations in various states $\left(\psi_{0}^{g}\chi ,\hat{\Phi }\left(t,x\right)\hat{\Phi }\left(x^{\prime }\right)\psi_{0}^{g}\chi \right)$ using the propagator (111) as

(118) $$\begin{array}{c} \int d\Phi d\Phi^{\prime }d\Phi^{\prime \prime }{\left(U_{t}\left(\Phi ,\Phi^{\prime }\right)\left(\psi_{0}^{g}\chi \right)\left(\Phi^{\prime }\right)\right)}^{*}\Phi \left(x\right)U_{t}\left(\Phi ,\Phi^{\prime \prime }\right)\Phi^{\prime \prime }\left(x^{\prime }\right)\left(\psi_{0}^{g}\chi \right)\left(\Phi^{\prime \prime }\right)\\ =\int d\Phi d\Phi^{\prime }d\Phi^{\prime \prime }{\left|\psi_{t}^{g}\left(\Phi \right)\right|}^{2}K_{t}{\left(\Phi ,\Phi^{\prime }\right)}^{*}K_{t}\left(\Phi ,\Phi^{\prime \prime }\right)\chi {\left(\Phi^{\prime }\right)}^{*}\chi \left(\Phi^{\prime \prime }\right)\Phi \left(x\right)\Phi^{\prime \prime }\left(x^{\prime }\right) \end{array}$$

with

(119) $$U_{t}\left(\Phi ,\Phi^{\prime }\right)=\psi_{t}^{g}\left(\Phi \right)K_{t}\left(\Phi ,\Phi^{\prime }\right){\left(\psi_{0}^{g}\left(\Phi^{\prime }\right)\right)}^{-1}.$$

We can express all multi-time correlation functions by the evolution kernel. As an example,

(120) $$\begin{array}{c} \left(\psi_{0}^{g}\chi ,{\hat{\Phi }}_{t}\left(x\right){\hat{\Phi }}_{t^{\prime }}\left(x^{\prime }\right)\psi_{0}^{g}\chi \right)=\left(U_{t}\psi_{0}^{g}\chi ,\Phi \left(x\right)U_{t,t^{\prime }}\Phi \left(x^{\prime }\right)\psi_{0}^{g}\chi \right) \end{array}$$

where

(121) $$U_{t,t^{\prime }}\left(\Phi ,\Phi^{\prime }\right)=\psi_{t}^{g}\left(\Phi \right)K_{\left(t,t^{\prime }\right)}\left(\Phi ,\Phi^{\prime }\right){\left(\psi_{t^{\prime }}^{g}\left(\Phi^{\prime }\right)\right)}^{-1}.$$

Hence, in terms of the kernels Equation (120) is expressed as

(122) $$\begin{array}{c} \int d\Phi d\Phi^{\prime }d\Phi^{\prime \prime }{\left(U_{t}\left(\Phi ,\Phi^{\prime }\right)\left(\psi_{0}^{g}\chi \right)\left(\Phi^{\prime }\right)\right)}^{*}\Phi \left(x\right)U_{t,t^{\prime }}\left(\Phi ,\Phi^{\prime \prime }\right)\Phi^{\prime \prime }\left(x^{\prime }\right)\left(\psi_{0}^{g}\chi \right)\left(\Phi^{\prime \prime }\right)=\\ \int d\Phi d\Phi^{\prime }d\Phi^{\prime \prime }{\left|\psi_{t}^{g}\left(\Phi \right)\right|}^{2}K_{t}{\left(\Phi ,\Phi^{\prime }\right)}^{*}K_{\left(t,t^{\prime }\right)}\left(\Phi ,\Phi^{\prime \prime }\right)\chi {\left(\Phi^{\prime }\right)}^{*}\chi \left(\Phi^{\prime \prime }\right)\Phi \left(x\right)\Phi^{\prime \prime }\left(x^{\prime }\right) \end{array}$$

The kernel $K_{\left(t,t^{\prime }\right)}$ is obtained as in Equation (109)

(123) $$K_{\left(t,t^{\prime }\right)}\left(\Phi ,\Phi^{\prime }\right)={\left(\det G\left(t,t^{\prime }\right)\right)}^{-\frac{1}{2}}\exp\left(-\frac{1}{2}\left(u_{0}{\left(u_{t-t^{\prime }}\right)}^{-1}\Phi -\Phi^{\prime }\right)G{\left(t,t^{\prime }\right)}^{-1}\left(u_{0}{\left(u_{t-t^{\prime }}\right)}^{-1}\Phi -\Phi^{\prime }\right)\right),$$

where now the process $\Phi_{s}$ is the solution of the stochastic equation with an initial value at $t^{\prime }$ (instead of zero)

(124) $$\Phi_{s}=u_{t-s}{\left(u_{t-t^{\prime }}\right)}^{-1}\Phi +\sqrt{i\hbar }u_{t-s}\int_{t^{\prime }}^{s}u_{t-\tau }^{-1}a_{t-\tau }^{-\frac{3}{2}}dW_{\tau }$$

Hence, from Equations (109) and (110),

(125) $$G\left(t,t^{\prime }\right)=i\hbar u_{0}^{2}\int_{t^{\prime }}^{t}u_{t-\tau }^{-2}a_{t-\tau }^{-3}d\tau$$

Similarly to Equation (122) in terms of the kernel $U_{t,t^{\prime }}\left(\Phi ,\Phi^{\prime }\right)$, we can express the *n*-point functions

(126) $$\left(\psi_{0}^{g}\chi ,{\hat{\Phi }}_{t_{1}}\left(x_{1}\right)\ldots \ldots {\hat{\Phi }}_{t_{n}}\left(x_{n}\right)\psi_{0}^{g}\chi \right)=\left(U_{t_{1}}\psi_{0}^{g}\chi ,\Phi \left(x_{1}\right)U_{t_{1},t_{2}}\Phi \left(x_{2}\right)\ldots \ldots U_{t_{n}-t_{n-1}}\Phi \left(x_{n}\right)\psi_{0}^{g}\chi \right)$$

In the presence of interaction, there are additional Feynman–Kac factors in $K_{\left(t,t^{\prime }\right)}$ as in Equation (48)

$$\exp\left(-\frac{i}{\hbar }\int_{t^{\prime }}^{t}V\left(\Phi_{s}\right)ds\right)$$

At the end of this section, we would like to discuss different notions of propagators in the literature [63,64] (the one for the metric a≃t has been discussed in [65,66]). In these papers, the propagator has been defined as the inverse of the operator A appearing in the action (22) when written in the form ∫dxL=∫dxΦAΦ. We could also represent this propagator by Schwinger’s proper time

$$A^{-1}=i\int_{0}^{\infty }d\tau \exp\left(-i\tau A\right).$$

By formal functional integration, the functional integral average $<\phi \left(x\right)\phi \left(x^{\prime }\right)>$ is equal to $A^{-1}\left(x,x^{\prime }\right)$(the kernel satisfying $AA^{-1}=1$). In general, by formal differentiation of the lhs of Equation (117) using Equation (23) in the metric (67), we obtain for t>0 under the assumption that the quantum field satisfies the wave equation

$$\begin{array}{c} \partial_{t}^{2}\left(\psi_{0}^{g}\chi ,{\hat{\Phi }}_{t}\left(k\right)\hat{\Phi }\left(k^{\prime }\right)\psi_{0}^{g}\chi \right)=\partial_{t}\left(\psi_{0}^{g}\chi ,a^{-3}\Pi_{t}\left(k\right)\hat{\Phi }\left(k^{\prime }\right)\psi_{0}^{g}\chi \right)\\ =\left(-3H\partial_{t}-a^{-2}k^{2}-M^{2}\right)\left(\psi_{0}^{g}\chi ,\Phi_{t}\left(k\right)\Phi \left(k^{\prime }\right)\psi_{0}^{g}\chi \right)\delta \left(k+k^{\prime }\right). \end{array}$$

Hence, the correlation function also satisfies the wave equation. If χ=1 and $|\psi_{t}^{g}{|}^{2}$ is integrable, then

$$\begin{array}{c} \left(\psi_{0}^{g},{\hat{\Phi }}_{t}\left(k\right)\hat{\Phi }\left(k^{\prime }\right)\psi_{0}^{g}\right)=\int d\Phi {\left|\psi_{t}^{g}\right|}^{2}\Phi \left(k\right)E\left[\Phi_{t}\left(k^{\prime }\right)\right]\\ =u_{0}\left(k\right)u_{t}^{*}\left(k\right)\delta \left(k+k^{\prime }\right) \end{array}$$

because $E\left[\Phi_{t}\left(k^{\prime }\right)\right]=\left(u_{0}u_{t}^{-1}\Phi \right)\left(k\right)$ (from Equation (82)) and $i\left(\Gamma -\Gamma^{*}\right)≃{\left(u_{t}u_{t}^{*}\right)}^{-1}$ from the Wronskian (as has been exploited in the particular case of the de Sitter metric (57) in Equation (90)). We can conclude that, although the correlation function in any state is a solution of the wave equation, then the solution of the equation for the correlation functions is not unique, because it depends on the state under consideration. If there is a unique ground state (as in de Sitter space) invariant under a symmetry group, then we can distinguish the solution having this invariance [51]. In other states, the two-point correlation function must be determined through calculations, e.g., from Equation (117) by means of the propagator (111) (as will be discussed at the end of Section 9).

In QFT in the Minkowski space-time in the ground state (11), we obtain $A^{-1}$ as the Lorentz invariant Green function of $\partial_{t}^{2}-▵+M^{2}$ equal to $\frac{1}{2}\nu^{-1}\exp(-i\nu |t-t^{\prime }\left|\right)$, where $\nu =\sqrt{-▵+M^{2}}$. In the case of the de Sitter space-time, the calculation with χ=1 and $\psi^{g}$ defined by Γ in Equation (90) coincides (as discussed after Equation (117)) with the result of an expectation value computed either by a formal functional integration or derived by an expansion of the field in creation and annihilation operators defined by de Sitter invariant vacuum [47,50,51]. In the time-dependent metric of this section (as well as in Section 8), there is no candidate for a vacuum. Hence, it remains unclear in which state $\psi_{0}^{g}\chi$ the two-point correlation function could be equal to $A^{-1}$. If there is no unique vacuum for the quantum field in an expanding metric, then the physical meaning of $A^{-1}$ is obscure, whereas the propagator defined in this section (and the correlation functions defined by the propagator) has a clear meaning for the canonically quantized field theory of Section 3. A relation of the propagator $K_{t}$ to $A^{-1}$, defined as the causal propagator, has been discussed in ref. [62]. In this paper, the definition of the propagator $K_{t}$ is related to the solution of the Cauchy problem, as expressed by the causal propagator. We briefly discuss the method of the calculation of $K_{t}$ using the solution of the Cauchy problem for A at the end of Appendix D.

## 8. Power-Law Expansion

In this and subsequent sections, we discuss some soluble wave equations. We are unable to derive an explicit solution of the wave Equation (68) in a homogeneous space-time for an arbitrary expansion a(t). In Appendix B, we solve this equation for a large *k* by means of the WKB method (it can be made exact as in [63]). Then, in this approximation, we can calculate the propagator (111) and the field correlation functions (Appendix B).

In this and subsequent sections, we discuss soluble models. First, we consider the model with the power-law expansion $a^{2}=b_{0}^{-2}{\left(t+\gamma \right)}^{2\alpha }$ with a general α∈R and t+γ>0 (we shift the initial time by γ in order to pose the initial condition at t=0, even if γ=0 corresponds to a degenerate metric). If t+γ<0, then *a* is a complex function in general (but even powers of *t* lead to admissible models with a topology change between positive and negative time [67]). We discuss the case $\alpha =\frac{1}{2}$ for t+γ<0 in Section 10. The expansion law $a=b_{0}^{-1}{\left|t+\gamma \right|}^{\alpha }$ for both positive t+γ and negative t+γ appears in cosmological models resulting from string theory [54,55]. Such a metric cannot be a solution of general relativity, because it is not differentiable at t+γ=0.

The wave equation for t+γ>0 has the form

(127) $$\partial_{t}^{2}u+3\alpha {\left(t+\gamma \right)}^{-1}\partial_{t}u+b_{0}^{2}{\left(t+\gamma \right)}^{-2\alpha }k^{2}u+M^{2}u=0.$$

The corresponding stochastic equation is

$$d\Phi_{s}=-u^{-1}\left(t-s\right)\partial_{t}u\left(t-s\right)ds+\sqrt{i\hbar }a^{-\frac{3}{2}}dW_{s}.$$

For t+γ<0 and $a=b_{0}^{-1}{\left|t+\gamma \right|}^{\alpha }$ (in string inspired models [54,55]), the wave equation is

$$\partial_{t}^{2}u+3\alpha {\left(t+\gamma \right)}^{-1}\partial_{t}u+b_{0}^{2}{\left|t+\gamma \right|}^{-2\alpha }k^{2}u+M^{2}u=0.$$

Equation (127) is explicitly soluble if M=0 ([68], 2.162 Equation (1a)). The solution is (for t+γ>0, we may choose here either complex-valued or real-valued Bessel functions)

(128) $$u={\left(t+\gamma \right)}^{\frac{1-3\alpha }{2}}Z_{\nu }\left(\frac{b_{0}}{1-\alpha }k{\left(t+\gamma \right)}^{1-\alpha }\right),$$

where

$$\nu =\frac{1-3\alpha }{2(1-\alpha )}.$$

α is related to *w* in the equation of state (p=wρ)

$$\alpha =\frac{2}{3(1+w)}.$$

Note that $\nu =-\frac{1}{2}$ if $\alpha =\frac{1}{2}$ (radiation, $w=\frac{1}{3}$) and $\nu =-\frac{3}{2}$ if $\alpha =\frac{2}{3}$ (dust, w=0). We discuss $\alpha =\frac{1}{2}$ in detail in Section 10.

Let us briefly consider the interesting case of $\alpha =\frac{2}{3}$. Then, a real solution valid for both t+γ>0, as well as t+γ<0, which is continuous at t+γ=0, is expressed by elementary functions ($T=3b_{0}{\left(t+\gamma \right)}^{\frac{1}{3}}$ is the conformal time)

$$\begin{array}{c} u={\left(t+\gamma \right)}^{-\frac{2}{3}}\left(\cos\left(3b_{0}k{\left(t+\gamma \right)}^{\frac{1}{3}}\right)-{\left(3b_{0}k{\left(t+\gamma \right)}^{\frac{1}{3}}\right)}^{-1}\sin\left(3b_{0}k{\left(t+\gamma \right)}^{\frac{1}{3}}\right)\right). \end{array}$$

The real solutions *u* are distinguished in our approach as they lead to real Γ and purely imaginary $G_{t}$ in Equation (112). This property is relevant for a construction of interactions via the Feynman–Kac formula (see Section 11). We also consider a complex solution, which gives a square integrable $\psi_{t}^{g}$

$$u={\left(t+\gamma \right)}^{-\frac{1}{2}}H_{\frac{3}{2}}^{\left(2\right)}\left(3b_{0}k{\left(t+\gamma \right)}^{\frac{1}{3}}\right)$$

The two-point function in this state is discussed at the end of this section.

For a general ν from Equation (69), we obtain

$$\Gamma ={\left(t+\gamma \right)}^{3\alpha }\frac{d}{dt}\ln\left({\left(t+\gamma \right)}^{\frac{1}{2}-\frac{3\alpha }{2}}Z_{\nu }\left(\frac{b_{0}}{1-\alpha }k{\left(t+\gamma \right)}^{1-\alpha }\right)\right).$$

The stochastic Equation (80) reads

(129) $$\begin{array}{c} d\Phi_{s}=-\frac{d}{dt}\ln\left({\left(t+\gamma -s\right)}^{\frac{1}{2}-\frac{3\alpha }{2}}Z_{\nu }\left(\frac{b_{0}}{1-\alpha }k{\left(t+\gamma -s\right)}^{1-\alpha }\right)\right)\Phi_{s}ds+\sqrt{i\hbar }{\left(t+\gamma -s\right)}^{-\frac{3\alpha }{2}}dW_{s}. \end{array}$$

The solution is

(130) $$\begin{array}{c} \Phi_{s}=\left({\left(t+\gamma -s\right)}^{\frac{1}{2}-\frac{3\alpha }{2}}Z_{\nu }\left(\frac{b_{0}}{1-\alpha }k{\left(t+\gamma -s\right)}^{1-\alpha }\right)\right)\\ {\left({\left(t+\gamma -t_{0}\right)}^{\frac{1}{2}-\frac{3\alpha }{2}}Z_{\nu }\left(\frac{1}{1-\alpha }k{\left(t+\gamma -t_{0}\right)}^{1-\alpha }\right)\right)}^{-1}\Phi \\ +\sqrt{i\hbar }\left({\left(t+\gamma -s\right)}^{\frac{1}{2}-\frac{3\alpha }{2}}Z_{\nu }\left(\frac{b_{0}}{1-\alpha }k{\left(t+\gamma -s\right)}^{1-\alpha }\right)\right)\\ \times \int_{t_{0}}^{s}d\tau {\left({\left(t+\gamma -\tau \right)}^{\frac{1}{2}-\frac{3\alpha }{2}}Z_{\nu }\left(\frac{b_{0}}{1-\alpha }k{\left(t+\gamma -\tau \right)}^{1-\alpha }\right)\right)}^{-1}{\left(t+\gamma -\tau \right)}^{-\frac{3}{2}\alpha }dW_{\tau }. \end{array}$$

Let

$$z\left(t+\gamma \right)=\frac{b_{0}k}{1-\alpha }{\left(t+\gamma \right)}^{1-\alpha }.$$

The general complex solution which can give a normalizable $\psi_{t}^{g}$ is a superposition of Hankel functions. The Wronskian for the Hankel functions is (here $H^{\left(2\right)}=H^{(1)*}$)

$$\left(\frac{d}{dz}H^{\left(2\right)}\right)H^{\left(1\right)}-\left(\frac{d}{dz}H^{\left(1\right)}\right)H^{\left(2\right)}=-\frac{4i}{\pi z}$$

Hence, for $Z_{\nu }=H_{\nu }^{\left(1\right)}$, we obtain

(131) $$\Gamma -\Gamma^{*}=\frac{4i}{\pi }{\left(H_{\nu }^{\left(2\right)}H_{\nu }^{(2)*}\right)}^{-1}{\left(t+\gamma \right)}^{3\alpha }H$$

Now, $\Gamma -\Gamma^{*}$ of Equation (131) leads to a normalizable Gaussian state (allowing a computation of correlation functions, an analog of Equation (90))

$$\begin{array}{c} \left(\psi_{0}^{g},{\hat{\Phi }}_{t}\left(k\right)\hat{\Phi }\left(k^{\prime }\right)\psi_{0}^{g}\right)=\int d\Phi {\left|\psi_{t}^{g}\left(\Phi \right)\right|}^{2}\Phi E\left[\Phi_{t}\left(\Phi \right)\right]\\ =\frac{\pi \hbar }{4H}H_{\nu }^{\left(2\right)}\left(z\left(\gamma \right)\right)H_{\nu }^{(2)*}\left(z\left(t+\gamma \right)\right){\left(t+\gamma \right)}^{1-\frac{3}{2}\alpha }\delta \left(k+k^{\prime }\right). \end{array}$$

We can express the correlations in the case of dust $\alpha =\frac{2}{3}$, $\nu =-\frac{3}{2}$ by elementary functions. The two-point function at small *k* behaves as

$$k^{-3}\exp\left(iz\left(t+\gamma \right)-iz\left(\gamma \right)\right)$$

It has the infrared singularity. $G_{t}$ is complex but the small *k* expansion contains a real part

$$ℜG_{t}≃\frac{t}{k}\delta \left(k+k^{\prime }\right)$$

similar to the one in de Sitter space.

The random fields defined by the Hankel function $H_{\nu }^{\left(2\right)}$ do not allow definition of the interaction by means of the Feynman–Kac integral in Section 11 and Section 12. We can establish the Feynman integral with the choice $J_{\nu }$ or $Y_{\nu }$ as $Z_{\nu }$. In such a case, after solving the Schrödinger equation with an interaction in Section 11, we must look for states which are square integrable and define the correlation functions by means of Equation (48) (these could be the Gaussian states of Equation (116)).

## 9. The Expansion $a^{2}\left(t\right)=\frac{\epsilon }{b_{0}^{2}}{\left(t+\gamma \right)}^{2}$

This is the limiting case (α→1) of Equation (127). It describes the $w=-\frac{1}{3}$ fluid corresponding to the coasting cosmology [69] or Dirac–Milne cosmology [70]. The scale a(t) is invariant under t+γ→−t−γ) (it is contracting for t+γ<0 and expanding for t+γ>0; we choose γ>0 for t>0 and γ<0 for t<0). The scalar field theory of these models has been also discussed in [65,66]. As noticed in Section 5, the Einstein equations which can appear in the path integral of quantum gravity together with $a^{2}≃t^{2}$ give also the solution with $a^{2}≃-t^{2}$ (with the same $w=-\frac{1}{3}$). The model with $a^{2}<0$ is interesting for the construction of an interaction via the Feynman–Kac formula, as will be discussed in Section 12.

The wave equation reads (with M=0)

(132) $$\frac{d^{2}u}{dt^{2}}+3{\left(t+\gamma \right)}^{-1}\frac{du}{dt}+\epsilon {\left(t+\gamma \right)}^{-2}b_{0}^{2}k^{2}u=0.$$

In Equation (132), we add a parameter ϵ=±1 in order to describe a model with an inverted spatial metric resulting from the solution of Friedmann equations $a^{2}=-\frac{1}{b_{0}^{2}}{\left(t+\gamma \right)}^{2}$ with $w=-\frac{1}{3}$. Choosing $\sqrt{-g}=i\sqrt{\left|g\right|}$, we obtain a diffusion equation for positive time instead of the Schrödinger equation. The diffusion equation makes sense only in one direction of time (either positive or negative). Our choice of the square root of $\sqrt{-g}$ in this section gives the diffusion equation for a positive time. The diffusion Equation (26) reads

(133) $$\begin{array}{c} \hbar \partial_{t}\psi_{t}=\frac{1}{2}\int dx\left(\hbar^{2}b_{0}^{3}{\left|t+\gamma \right|}^{-3}\frac{\delta^{2}}{\delta \Phi {\left(x\right)}^{2}}-\frac{\left|t+\gamma \right|}{b_{0}}{\left(\nabla \Phi \right)}^{2}-\mu^{2}b_{0}^{-3}{\left|t+\gamma \right|}^{3}\Phi^{2}\right)\psi_{t} \end{array}$$

The equation for χ is

(134) $$\partial_{t}\chi_{t}=\int dx\left(\hbar \frac{1}{2}b_{0}^{-3}{\left|t+\gamma \right|}^{-3}\frac{\delta^{2}}{\delta \Phi {\left(x\right)}^{2}}-u^{-1}\partial_{t}u\Phi \left(x\right)\frac{\delta }{\delta \Phi (x)}\right)\chi_{t},$$

where *u* is the solution of Equation (132) with ϵ=−1. We could consider the model of Section 3, where there is $dx\sqrt{\left|g\right|}$ as the volume element. In such a case, we still have the Schrödinger Equation (instead of the diffusion equation). Then, the equation for χ is

$$\partial_{t}\chi_{t}=\int dx\left(\hbar \frac{i}{2}b_{0}^{3}{\left|t+\gamma \right|}^{-3}\frac{\delta^{2}}{\delta \Phi {\left(x\right)}^{2}}-u^{-1}\partial_{t}u\Phi \left(x\right)\frac{\delta }{\delta \Phi (x)}\right)\chi_{t},$$

where *u* satisfies Equation (132) with ϵ=−1. We could also treat the introduction of ϵ in Equation (132) as a technical step for a derivation of solutions, which subsequently are to be continued analytically to ϵ=1. There is no continuous transition between ϵ=−1 and ϵ=1.

The solution of Equation (132) is [68] (true for t+γ>0 as well as for t+γ<0)

(135) $$u=C_{1}{\left|t+\gamma \right|}^{-1-\mu }+C_{2}{\left|t+\gamma \right|}^{-1+\mu },$$

where

(136) $$\mu =\sqrt{1-\epsilon b_{0}^{2}k^{2}}.$$

The conformal time is

$$T=\int dta^{-1}=b_{0}\ln\left(t+\gamma \right).$$

Hence, the classical solution in conformal time

$$u=C_{1}\exp\left(-b_{0}^{-1}T\left(\mu +1\right)\right)+C_{2}\exp\left(-b_{0}^{-1}T\left(-\mu +1\right)\right)$$

for large momenta (with ϵ=1) $\mu ≃ikb_{0}$ looks like a free wave (with a decaying amplitude).

We choose $C_{1}=0$. Then,

$$i\Gamma =-{\left|t\right|}^{3}u^{-1}\partial_{t}u=-t^{2}\left(\mu -1\right)<0.$$

Hence, $\psi_{t}^{g}$ (40) is square integrable.

The solution of the stochastic Equation (80) for the model (134) is

(137) $$\Phi_{s}=u_{t-s}u_{t}^{-1}\Phi +\sqrt{\hbar }u_{t-s}b_{0}^{\frac{3}{2}}\int_{0}^{s}u_{t-\tau }^{-1}{\left(t+\gamma -\tau \right)}^{-\frac{3}{2}}dW_{\tau }.$$

We are interested in an explicit calculation of the evolution kernel and correlation functions. In such calculations, we need to evaluate

$$\begin{array}{c} G_{s,s^{\prime }}\left(k,k^{\prime }\right)=E\left[\left(\Phi_{s^{\prime }}\left(k\right)-<\Phi_{s^{\prime }}>\left(k\right)\right)\left(\Phi_{s}\left(k^{\prime }\right)-<\Phi_{s}>\left(k^{\prime }\right)\right)\right]\equiv G_{s,s^{\prime }}\left(k\right)\delta \left(k+k^{\prime }\right), \end{array}$$

where <Φ>≡E[Φ].

The operator $\mu =\sqrt{1+\epsilon b_{0}^{2}▵}$ with ϵ=1 cannot be defined in the infinite dimensional setting. For this reason, we consider the model with the inverted signature (ϵ=−1) ▵→−▵ when μ is a self-adjoint positive operator in $L^{2}\left(dx\right)$. When $C_{1}=0$ then, with the inverted signature, the operator $u_{t-s}u_{t}^{-1}=\frac{t+\gamma }{t+\gamma -s}{\left(\frac{t+\gamma -s}{t+\gamma }\right)}^{\mu }$ is well-defined as a contractive semigroup acting upon Φ in Equation (137).

We have (with $C_{1}=0$ and t+γ>0) the following for Model (134):

(138) $$\begin{array}{c} G_{s,s^{\prime }}\left(k\right)=\hbar u_{t-s}u_{t-s^{\prime }}\int_{0}^{m(s,s^{\prime })}d\tau u_{t-\tau }^{-2}{\left(t+\gamma -\tau \right)}^{-3}\\ =\frac{\hbar }{2\mu }{\left(t-s+\gamma \right)}^{-1+\mu }{\left(t-s^{\prime }+\gamma \right)}^{-1+\mu }\\ \left({\left(t+\gamma -m\left(s,s^{\prime }\right)\right)}^{-2\mu }-{\left(t+\gamma \right)}^{-2\mu }\right), \end{array}$$

where $m\left(s,s^{\prime }\right)=min\left(s,s^{\prime }\right)$.

It can be checked that the rhs of Equation (138) is expressed by a well-defined operator exp(−rμ) (ϵ=−1), where r≥0.

For equal time in Model (138), we have

(139) $$\begin{array}{c} G_{t}=\frac{\hbar }{2\mu }\gamma^{-2}\left(1-{\left(1+\frac{t}{\gamma }\right)}^{-2\mu }\right) \end{array}$$

The limit γ=0 is infinite expressing the degeneracy of the metric at t+γ=0. The evolution kernel is defined by $G_{t}$ in Equation (111). We can express the correlation function of Equation (138) by the two-point function $G_{M}^{E}$ of the scalar free field with a mass M using the formula ($\nu =\sqrt{k^{2}+M^{2}}$)

(140) $$\begin{array}{c} {\left(2\pi \right)}^{-3}\int dk\exp\left(i\mathrm{kx}\right){\left(2\nu \right)}^{-1}\exp\left(-s\nu \right)\\ ={\left(2\pi \right)}^{-4}\int dk_{0}dk\exp\left(i\mathrm{kx}+ik_{0}s\right){\left(k_{0}^{2}+k^{2}+M^{2}\right)}^{-1}=G_{M}^{E}\left(s,x\right) \end{array}$$

It can be seen from Equations (138) and (140) that the random field $\Phi_{s}\left(k\right)$ has correlation function with the same large *k* behavior (the same short distance behavior) as the quantum Euclidean free field (after the signature inversion). This holds true for all stochastic fields defined in this paper. In the construction of polynomial interactions (Section 11 and Section 12), and in the expansion of non-polynomial interactions in powers of the fields [71], we shall have the same ultraviolet singularity and the same renormalization problem as in the conventional Fock space approach or in the constructive Euclidean framework. However, in the standard approach to QFT in four dimensions, we cannot go beyond the perturbative framework because after a renormalization the Feynman–Kac factor becomes unbounded. In non-polynomial interactions the superpropagator [71] becomes extremely singluar. This can change in the stochastic approach (as discussed in Section 11 and Section 12), because we can work with (bounded) Feynman–Kac oscillatory factors (in particular, the superpropagator becomes an oscillatory hence Lebesgue integrable function).

## 10. The Radiation Background $a^{2}\left(t\right)=c_{0}^{-1}\left(t+\gamma \right)$

In this section, we consider the massless scalar field with $\alpha =\frac{1}{2}$ in Equation (132). We insert γ≥0 for t≥0 and γ≤0 for t≤0, so that when γ≠0, the metric is not degenerate. The metric (for t+γ>0) is the solution of the Friedmann equation for radiation with the energy density $\rho =\rho_{0}a^{-4}$ and the pressure $p=\frac{1}{3}\rho$. It is usually rejected at t+γ<0 because the inverted signature has no classical meaning ([72], Section 112), as it violates the local special relativity principles. The inverted metric can appear as a stationary point in quantum gravity, defined as an average over the metric tensor. The causal structure in quantum gravity can disagree with the classical one at the Planck scale.

We are interested in the behavior of the quantum scalar field evolution for the metric $a^{2}\left(t\right)=c_{0}^{-1}\left(t+\gamma \right)$ for positive as well as negative time in the limit γ=0. The Gaussian solution (40) is determined by a solution of the equation

(141) $$\frac{d^{2}u}{dt^{2}}+\frac{3}{2}{\left(t+\gamma \right)}^{-1}\frac{du}{dt}+{\left(t+\gamma \right)}^{-1}c_{0}k^{2}u=0$$

true for t+γ>0, as well as for t+γ<0.

For t+γ<0 we choose $\sqrt{-g}=-i\sqrt{\left|g\right|}$. With such a choice of the square root, the diffusion Equation (26) reads

(142) $$\begin{array}{c} \hbar \partial_{t}\psi_{t}=\frac{1}{2}\int dx(-\hbar^{2}c_{0}^{\frac{3}{2}}{\left|t+\gamma \right|}^{-\frac{3}{2}}\frac{\delta^{2}}{\delta \Phi {\left(x\right)}^{2}}\\ +c_{0}^{-\frac{1}{2}}{\left|t+\gamma \right|}^{\frac{1}{2}}{\left(\nabla \Phi \right)}^{2}-M^{2}c_{0}^{-\frac{3}{2}}{\left|t+\gamma \right|}^{\frac{3}{2}}\Phi^{2})\psi_{t} \end{array}$$

The equation for χ is

(143) $$\partial_{t}\chi_{t}=\int dx\left(-\hbar \frac{1}{2}c_{0}^{\frac{3}{2}}{\left|t+\gamma \right|}^{-\frac{3}{2}}\frac{\delta^{2}}{\delta \Phi {\left(x\right)}^{2}}-u^{-1}\partial_{t}u\Phi \left(x\right)\frac{\delta }{\delta \Phi (x)}\right)\chi_{t}.$$

Equations (142) and (143) are well-defined for t+γ<0, because the generator of the diffusion has a positively definite second order differential operator.

We could consider the metric $g_{lk}=\delta_{lk}c_{0}^{-1}\left|t+\gamma \right|$, which does not have the second derivative at t+γ=0. For this reason, it cannot be a solution of Einstein equations, but appears as a solution in the effective field theory resulting from the string theory [54]. For this metric, the Schrödinger Equation (25) reads

$$\partial_{t}\chi_{t}=\frac{1}{2}\int dx\left(i\hbar \frac{1}{2}c_{0}^{\frac{3}{2}}{\left|t+\gamma \right|}^{-\frac{3}{2}}\frac{\delta^{2}}{\delta \Phi {\left(x\right)}^{2}}-u^{-1}\partial_{t}u\Phi \left(x\right)\frac{\delta }{\delta \Phi (x)}\right)\chi_{t},$$

where *u* is the solution of the equation

(144) $$\frac{d^{2}u}{dt^{2}}+\frac{3}{2}{\left(t+\gamma \right)}^{-1}\frac{du}{dt}+{\left|t+\gamma \right|}^{-1}c_{0}k^{2}u=0.$$

In order to obtain a solution of Equation (144), it is useful to change the cosmic time *t* into the conformal time *T* as $T=2c_{0}^{\frac{1}{2}}\sqrt{t}$ for t>0 and $T=-2c_{0}^{\frac{1}{2}}\sqrt{-t}$ for t<0 (then $a^{2}\left(T\right)≃T^{2}$ as in [73]). Inserting $\tilde{u}=Tu$ into Equation (144), we can see that $\tilde{u}$ satisfies the oscillator equation. Hence, the solution of Equation (144) for t+γ>0 is a superposition of plane waves

$$u=A_{1}T^{-1}\exp\left(ikT\right)+A_{2}T^{-1}\exp\left(-ikT\right).$$

It follows that the solution of Equation (144) for positive as well as negative time is

(145) $$\begin{array}{c} u_{t}=C_{1}{\left|t+\gamma \right|}^{-\frac{1}{2}}\cos\left(2k\sqrt{c_{0}}\sqrt{|t+\gamma }|\right)+C_{2}{\left|t+\gamma \right|}^{-\frac{1}{2}}\sin\left(2k\sqrt{c_{0}}\sqrt{\left|t+\gamma \right|}\right) \end{array}$$

We obtain a different solution of Equation (141). We express it by real functions (for t+γ>0)

(146) $$\begin{array}{c} u_{t}=C_{1}{\left(t+\gamma \right)}^{-\frac{1}{2}}\cos\left(2k\sqrt{c_{0}}\sqrt{t+\gamma }\right)+C_{2}{\left(t+\gamma \right)}^{-\frac{1}{2}}\sin\left(2k\sqrt{c_{0}}\sqrt{t+\gamma }\right) \end{array}$$

If t+γ<0, then the solution of Equation (141) is

(147) $$\begin{array}{c} u_{t}^{\left(-\right)}=C_{1}{\left(-t-\gamma \right)}^{-\frac{1}{2}}\cosh\left(2k\sqrt{c_{0}}\sqrt{-t-\gamma }\right)+C_{2}{\left(-t-\gamma \right)}^{-\frac{1}{2}}\sinh\left(2k\sqrt{c_{0}}\sqrt{-t-\gamma }\right) \end{array}$$

For t+γ<0 Equation (141) (with $k^{2}\to -▵$) is an elliptic equation. It does not describe waves.

The limit γ→0 of $u_{t}$ exists for all |t|≥0 (see a discussion of continuity in [67,74,75,76]) only if $C_{1}=0$. Then, the limit t+γ→0 for positive time, as well as for the negative time, is equal to $u_{0}=2C_{2}\sqrt{c_{0}}k$. The limit t+γ→0 of $\partial_{t}u_{t}$ also exists from both sides

$${\left(\partial_{t}u_{t}\right)}_{|t=0}=-\frac{4}{3}C_{2}\sqrt{c_{0}}c_{0}k^{3}.$$

The solution of the stochastic Equation (80) for t≥s≥0 is

(148) $$\begin{array}{c} \Phi_{s}\left(\Phi \right)=u_{t-s}u_{t}^{-1}\Phi +\sqrt{i\hbar }u_{t-s}c_{0}^{\frac{3}{4}}\int_{0}^{s}u_{t-\tau }^{-1}{\left|t+\gamma -\tau \right|}^{-\frac{3}{4}}dW_{\tau }. \end{array}$$

For −t≥−s≥0 of the (string) metric, $a\left(t\right)≃{\left|t\right|}^{\frac{1}{2}}$ the field $\Phi_{s}$ determined by Equations (77) and (84) is

(149) $$\begin{array}{c} \Phi_{s}\left(\Phi \right)=u_{t-s}u_{t}^{-1}\Phi +\sqrt{-i\hbar }u_{t-s}c_{0}^{\frac{3}{4}}\int_{0}^{-s}u_{t-\tau }^{-1}{\left|t+\gamma +\tau \right|}^{-\frac{3}{4}}dW_{\tau }. \end{array}$$

The quantum field theory depends (for a positive time) on the correlation function

(150) $$\begin{array}{c} G_{ss^{\prime }}=E\left[\left(\Phi_{s}\left(k\right)-E\left[\Phi_{s}\right]\left(k\right)\right)\left(\Phi_{s^{\prime }}\left(k^{\prime }\right)-E\left[\Phi_{s^{\prime }}\right]\left(k^{\prime }\right)\right)\right]=\delta \left(k+k^{\prime }\right)i\hbar c_{0}^{\frac{3}{2}}u_{t-s}u_{t-s^{\prime }}\\ \times \int_{0}^{m(s,s^{\prime })}d\tau u_{t-\tau }^{-2}{\left|t+\gamma -\tau \right|}^{-\frac{3}{2}}\equiv \delta \left(k+k^{\prime }\right)G_{ss^{\prime }}\left(k\right). \end{array}$$

where we denote $m\left(s,s^{\prime }\right)\equiv min\left(s,s^{\prime }\right)$ if t≥s≥0 and $t\geq s^{\prime }\geq 0$. The $\delta (k+k^{\prime })$ term in Equation (150) will be omitted in the formulas below.

For a negative time of $a^{2}≃t$ in the model (141)–(143), there is no $\sqrt{i}$ in Equation (82), which is canceled by the $\sqrt{i}$ factor in $a^{-\frac{3}{2}}$. Hence, the counterpart of Equation (149) for the negative time leads to the diffusion process $\Phi_{s}$ solving the diffusion Equation (143)

$$\begin{array}{c} \Phi_{s}\left(\Phi \right)=u_{t-s}^{\left(-\right)}{\left(u_{t}^{\left(-\right)}\right)}^{-1}\Phi +\sqrt{\hbar }u_{t-s}^{\left(-\right)}c_{0}^{\frac{3}{4}}\int_{0}^{-s}{\left(u_{t-\tau }^{\left(-\right)}\right)}^{-1}{\left|t+\gamma +\tau \right|}^{-\frac{3}{4}}dW_{\tau }, \end{array}$$

where, by $u_{t}^{\left(-\right)}$, we denote the solution (147) of the “wave equation” (141) for the negative time. The correlation functions of the fields for a negative time are determined by the formula

(151) $$\begin{array}{c} G_{ss^{\prime }}=E\left[\left(\Phi_{s}\left(k\right)-E\left[\Phi_{s}\right]\left(k\right)\right)\left(\Phi_{s^{\prime }}\left(k^{\prime }\right)-E\left[\Phi_{s^{\prime }}\right]\left(k^{\prime }\right)\right)\right]\\ =\delta \left(k+k^{\prime }\right)\hbar c_{0}^{\frac{3}{2}}u_{t-s}^{\left(-\right)}u_{t-s^{\prime }}^{\left(-\right)}\\ \times \int_{0}^{m(s,s^{\prime })}d\tau {\left(u_{t-\tau }^{\left(-\right)}\right)}^{-2}{\left|t+\gamma +\tau \right|}^{-\frac{3}{2}}\equiv \delta \left(k+k^{\prime }\right)G_{ss^{\prime }}\left(k\right), \end{array}$$

where, for the negative time $m\left(s,s^{\prime }\right)\equiv min\left(-s,-s^{\prime }\right)$, if −t≥−s≥0 and $-t\geq -s^{\prime }\geq 0$.

As can be seen from Equation (143), for a negative time, $\Phi_{s}$ becomes a real diffusion process and $K_{t}$ in Equation (111) is a real transition function. The Schrödinger evolution equation (together with the Feynman–Kac formula) takes the form of an evolution of the diffusion process. The reason for this is the purely imaginary value of $\sqrt{-g}$. If $a\left(t\right)≃{\left|t+\gamma \right|}^{\frac{1}{2}}$ then the expression for $\Phi_{s}$ and $G_{ss^{\prime }}$ for negative time resembles the ones for the positive time (for a negative time it is a reflection of the one for a positive time).

We can express the integrals (150) and (151) by elementary functions if $C_{2}=0$, $C_{1}=0$, $C_{1}=\pm C_{2}$ and $C_{1}=\pm iC_{2}$.

If $C_{2}=0$, then we have, for t+γ>0,

(152) $$u_{t}={\left(t+\gamma \right)}^{-\frac{1}{2}}\cos\left(2\sqrt{c_{0}}k\sqrt{t+\gamma }\right).$$

If $C_{1}=0$, then

(153) $$u_{t}={\left(t+\gamma \right)}^{-\frac{1}{2}}\sin\left(2\sqrt{c_{0}}k\sqrt{t+\gamma }\right).$$

If −t−γ>0, then

(154) $$u_{t}^{\left(-\right)}={\left(-t-\gamma \right)}^{-\frac{1}{2}}\cosh\left(2\sqrt{c_{0}}k\sqrt{-t-\gamma }\right)$$

and

(155) $$u_{t}^{\left(-\right)}={\left(-t-\gamma \right)}^{-\frac{1}{2}}\sinh\left(2\sqrt{c_{0}}k\sqrt{-t-\gamma }\right).$$

When $C_{1}=\pm C_{2}$, then

(156) $$u_{t}^{\left(-\right)}={\left(-t-\gamma \right)}^{-\frac{1}{2}}\exp\left(\pm 2\sqrt{c_{0}}k\sqrt{-t-\gamma }\right).$$

For the solution (152), we obtain

(157) $$\begin{array}{c} G_{ss^{\prime }}=i\hbar u_{t-s}u_{t-s^{\prime }}c_{0}^{\frac{3}{2}}\int_{0}^{m(s,s^{\prime })}d\tau {\left|t+\gamma -\tau \right|}^{-\frac{1}{2}}{\left(\cos\left(2\sqrt{c_{0}}k\sqrt{t+\gamma -\tau }\right)\right)}^{-2}\\ =i\hbar u_{t-s}u_{t-s^{\prime }}c_{0}\frac{1}{k}\left(\tan\left(2\sqrt{c_{0}}k\sqrt{t-m(s,s^{\prime })+\gamma }\right)-\tan\left(2\sqrt{c_{0}}k\sqrt{t+\gamma }\right)\right) \end{array}$$

where

(158) $$u_{0}=\frac{1}{\sqrt{\gamma }}\cos\left(2\sqrt{c_{0}}k\sqrt{\gamma }\right).$$

The limit γ→0 of $u_{t}$ does not exist at t=0. Then, the evolution kernel (111) is not defined. For the solution (153),

(159) $$\begin{array}{c} G_{ss^{\prime }}=i\hbar u_{t-s}u_{t-s^{\prime }}c_{0}^{\frac{3}{2}}\int_{0}^{m(s,s^{\prime })}d\tau {\left|t+\gamma -\tau \right|}^{-\frac{1}{2}}{\left(\sin\left(2\sqrt{c_{0}}k\sqrt{t+\gamma -\tau }\right)\right)}^{-2}\\ =i\hbar u_{t-s}u_{t-s^{\prime }}c_{0}\frac{1}{k}\left(\cot\left(2\sqrt{c_{0}}k\sqrt{t+\gamma }\right)-\cot\left(2\sqrt{c_{0}}k\sqrt{t-m(s,s^{\prime })+\gamma }\right)\right) \end{array}$$

The limit γ→0 of $u_{0}$ in Equation (153) is $2\sqrt{c_{0}}k$. When t→0 and γ→0 then $m(s,s^{\prime })\to 0$ and $G_{ss^{\prime }}\to 0$ in Equation (159).

For the solution (154), we obtain at t+γ<0 in Equation (151)

(160) $$\begin{array}{c} G_{ss^{\prime }}=\hbar u_{t-s}^{\left(-\right)}u_{t-s^{\prime }}^{\left(-\right)}c_{0}^{\frac{3}{2}}\int_{0}^{m(s,s^{\prime })}d\tau {\left|t+\gamma +\tau \right|}^{-\frac{1}{2}}{\left(\cosh\left(2\sqrt{c_{0}}k\sqrt{-t-\gamma -\tau }\right)\right)}^{-2}\\ =\hbar u_{t-s}^{\left(-\right)}u_{t-s^{\prime }}^{\left(-\right)}c_{0}\frac{1}{k}(\tanh\left(2\sqrt{c_{0}}k\sqrt{-t-m(s,s^{\prime })-\gamma }\right)\\ -\tanh(2\sqrt{c_{0}}k\sqrt{-t-\gamma })). \end{array}$$

$u_{0}$ has no limit when γ→0. Hence, the propagator (111) cannot be defined in this limit.

For $u_{t}^{\left(-\right)}$ of Equation (155), we have

(161) $$\begin{array}{c} G_{ss^{\prime }}=\hbar u_{t-s}^{\left(-\right)}u_{t-s^{\prime }}^{\left(-\right)}c_{0}^{\frac{3}{2}}\int_{0}^{m(s,s^{\prime })}d\tau {\left|t+\gamma +\tau \right|}^{-\frac{1}{2}}{\left(\sinh\left(2\sqrt{c_{0}}k\sqrt{-t-\gamma -\tau }\right)\right)}^{-2}\\ =\hbar u_{t-s}^{\left(-\right)}u_{t-s^{\prime }}^{\left(-\right)}c_{0}\frac{1}{k}(\coth\left(2\sqrt{c_{0}}k\sqrt{-t-\gamma }\right)\\ -\coth(2\sqrt{c_{0}}k\sqrt{-t-m(s,s^{\prime })-\gamma })) \end{array}$$

The limit γ→0 of $u_{0}$ in Equation (161) together with Equation (111) also defines the evolution kernel in the limit γ→0. There is an apparent singularity as k→0 in the correlation $G_{ss^{\prime }}\left(k\right)$, but this singularity is canceled by the volume element dk in the definition of the evolution kernel (111).

$G_{ss^{\prime }}$ (161) is decaying for a large *k* as

$$\begin{array}{c} G_{ss^{\prime }}≃\hbar \frac{1}{2}c_{0}{\left(-t+s-\gamma \right)}^{-\frac{1}{2}}{\left(-t+s^{\prime }-\gamma \right)}^{-\frac{1}{2}}k^{-1}\\ (\exp(-4\sqrt{c_{0}}k\sqrt{-t-m(s,s^{\prime })-\gamma }+2\sqrt{c_{0}}k\sqrt{-t-\gamma +s}\\ +2\sqrt{c_{0}}k\sqrt{-t-\gamma +s^{\prime }})\\ -\exp(-4\sqrt{c_{0}}k\sqrt{-t-\gamma }+2\sqrt{c_{0}}k\sqrt{-t-\gamma +s}\\ +2\sqrt{c_{0}}k\sqrt{-t-\gamma +s^{\prime }})) \end{array}$$

The correlation functions of the field $\Phi_{s}\left(x\right)$ can be expressed (according to Equation (150) as the Fourier transform of $G_{ss^{\prime }}$. From the large *k* behavior of (161) and Equation (140), we can conclude that the short distance behavior of the correlations of $\Phi_{s}\left(x\right)$ is the same as in the Euclidean free field theory of the scalar field.

The exponential solutions define the correlation functions

(162) $$\begin{array}{c} G_{ss^{\prime }}=\hbar u_{t-s}^{\left(-\right)}u_{t-s^{\prime }}^{\left(-\right)}c_{0}^{\frac{3}{2}}\int_{0}^{m(s,s^{\prime })}d\tau {\left|t+\gamma +\tau \right|}^{-\frac{1}{2}}{\left(\exp\left(-2\sqrt{c_{0}}k\sqrt{-t-\gamma -\tau }\right)\right)}^{-2}\\ =\hbar u_{t-s}^{\left(-\right)}u_{t-s^{\prime }}^{\left(-\right)}c_{0}\frac{1}{2k}(\exp\left(4\sqrt{c_{0}}k\sqrt{-t-m(s,s^{\prime })-\gamma }\right)\\ -\exp(4\sqrt{c_{0}}k\sqrt{-t-\gamma })) \end{array}$$

for the minus sign in (156) and

(163) $$\begin{array}{c} G_{ss^{\prime }}=i\hbar u_{t-s}^{\left(-\right)}u_{t-s^{\prime }}^{\left(-\right)}c_{0}^{\frac{3}{2}}\\ \times \int_{0}^{m(s,s^{\prime })}d\tau {\left|t+\gamma +\tau \right|}^{-\frac{1}{2}}{\left(\exp\left(2k\sqrt{-t-\gamma -\tau }\right)\right)}^{-2}\\ =i\hbar u_{t-s}^{\left(-\right)}u_{t-s^{\prime }}^{\left(-\right)}c_{0}\frac{1}{2k}(\exp\left(-4\sqrt{c_{0}}k\sqrt{-t-\gamma }\right)\\ -\exp(-4\sqrt{c_{0}}k\sqrt{-t-m(s,s^{\prime })-\gamma })) \end{array}$$

for the plus sign. There is no limit γ→0 of $u_{0}$.

At the end of this section, let us consider some superpositions of the solutions (152) and (153) with complex coefficients. So for positive time, let us consider (for a negative time the solution of the “wave equation” (141) is given by Equation (147))

(164) $$u_{t}={\left(t+\gamma \right)}^{-\frac{1}{2}}\exp\left(2i\sqrt{c_{0}}k\sqrt{t+\gamma }\right).$$

Calculating the field correlations, we obtain

(165) $$\begin{array}{c} G_{ss^{\prime }}=\frac{\hbar }{2}\frac{1}{\sqrt{c_{0}}k}\frac{1}{\sqrt{t+\gamma -s}}\frac{1}{\sqrt{t+\gamma -s^{\prime }}}\exp\left(2i\sqrt{c_{0}}k\left(\sqrt{t+\gamma -s}+\sqrt{t+\gamma -s^{\prime }}\right)\right)\\ \left(\exp\left(-4i\sqrt{c_{0}}k\sqrt{t+\gamma -m(s,s^{\prime }}\right)-\exp\left(-4i\sqrt{c_{0}}k\sqrt{t+\gamma }\right)\right) \end{array}$$

For $G_{t}$ in the propagator (111), we have

(166) $$\begin{array}{c} G_{t}=-\frac{1}{2\gamma \sqrt{c_{0}}k}\left(\exp\left(-4i\sqrt{c_{0}}k\sqrt{t+\gamma }\right)+4i\sqrt{c_{0}}k\sqrt{\gamma })-1\right)\\ =-\frac{1}{2\gamma \sqrt{c_{0}}k}(\cos\left(-4\sqrt{c_{0}}k\sqrt{t+\gamma }\right)+4\sqrt{c_{0}}k\sqrt{\gamma })-1\\ -i\sin\left(-4\sqrt{c_{0}}k\sqrt{t+\gamma }\right)+4\sqrt{c_{0}}k\sqrt{\gamma })) \end{array}$$

Note that $ℜG_{t}>0$. Hence, the function defining $K_{t}\left(\Phi ,\Phi^{\prime }\right)$ in Equation (111) is integrable (this would not be so if, instead of $u_{t}$ in Equation (164), we have considered $u_{t}^{*}$).

With a complex $u_{t}$, the solution $\psi_{t}^{g}$ (40) of the Schrödinger equation is not a phase factor, and it may grow to infinity for a large Φ (this would be so for $u_{t}^{*}$). Let us calculate from Equation (69) for $u_{t}$ of Equation (164)

(167) $$i\Gamma =a^{3}u^{-1}\partial_{t}u=-\sqrt{c_{0}}k\left(t+\gamma \right)-\frac{i}{2}\sqrt{t+\gamma }.$$

Hence, ℜ(iΓ)<0, showing that $\psi_{t}^{g}$ is square integrable for the complex solution (164) for positive time. The correlation function is

$$\begin{array}{c} \left(\psi_{0}^{g}{\hat{\Phi }}_{t}\left(k\right)\hat{\Phi }\left(k^{\prime }\right)\psi_{0}^{g}\right)=\gamma^{-\frac{1}{2}}\exp\left(2i\sqrt{c_{0}}k\sqrt{\gamma }\right){\left(t+\gamma \right)}^{\frac{1}{2}}\exp\left(-2i\sqrt{c_{0}}k\sqrt{t+\gamma }\right)\\ \times {\left(2\sqrt{c_{0}}k\left(t+\gamma \right)\right)}^{-1}\delta \left(k+k^{\prime }\right) \end{array}$$

Then, for a small time,

$$\begin{array}{c} \left(\psi_{0}^{g},\Phi \left(k\right)\Phi \left(k^{\prime }\right)\psi_{0}^{g}\right)-\left(\psi_{0}^{g}{\hat{\Phi }}_{t}\left(k\right)\hat{\Phi }\left(k^{\prime }\right)\psi_{0}^{g}\right)≃\left(t{\left(4\sqrt{c_{0}}k\gamma^{2}\right)}^{-1}+it\gamma^{-\frac{3}{2}}\right)\delta \left(k+k^{\prime }\right) \end{array}$$

This real and imaginary parts of the diffusive (linear in *t*) behavior of $\Phi^{2}$ can also be seen from Equation (165).

For the negative time with the solution

(168) $$u_{t}^{\left(-\right)}={\left(-t-\gamma \right)}^{-\frac{1}{2}}\exp\left(2\sqrt{c_{0}}k\sqrt{-t-\gamma }\right)$$

$\psi_{t}^{g}$ is also square integrable, as $i\Gamma ={\left(a^{2}\right)}^{\frac{3}{2}}u^{\left(-\right)}\partial_{t}u^{\left(-\right)}<0$. For the solutions (164) and (168) in the free field theory in the radiation background, we can calculate correlation functions using the Formula (47), as we did in the case of the de Sitter background in Equations (90) and (91). However, with these solutions, $u_{t}$ it is difficult to define the interaction via the Feynman–Kac formula, because we are unable to prove that the Feynman–Kac factor is a bounded function (as discussed in [36] and in Section 11 and Section 12).

We may consider more general superpositions of solutions of wave Equation (141) by an addition of a piece with negative frequency to Equation (164) for a positive time

(169) $$u_{t}={\left(t+\gamma \right)}^{-\frac{1}{2}}(\exp\left(2i\sqrt{c_{0}}k\sqrt{t+\gamma }\right)+\left(\alpha +i\beta \right)\exp\left(-2i\sqrt{c_{0}}k\sqrt{t+\gamma }\right)$$

and

(170) $$u_{t}^{\left(-\right)}={\left(-t-\gamma \right)}^{-\frac{1}{2}}(\exp\left(2\sqrt{c_{0}}k\sqrt{-t-\gamma }\right)+\left(\alpha +i\beta \right)\exp\left(-2\sqrt{c_{0}}k\sqrt{-t-\gamma }\right).$$

for a negative time. We can calculate $\Gamma =a^{3}u^{-1}\partial_{t}u$ for a positive time and $\Gamma =-i(|a^{2}{\left|\right)}^{\frac{3}{2}}{\left(u^{\left(-\right)}\right)}^{-1}\partial_{t}u^{\left(-\right)}$ for a negative time. We did not find square integrable solutions (ℜ(iΓ)<0), except for the cases (164) and (168), leading to a square integrable wave function for a quantum scalar field in a radiation background. The limit γ→0 of the degenerate metric does not exist from both sides of time except of the solutions (153) and (155).

Let us summarize the results of this section. When we choose real solutions *u*, then, for a positive time (when −g>0), Γ is real. Hence, $\psi_{t}^{g}$ is a pure phase. $G_{t}$ (112) (as well as $G_{ss^{\prime }}$) is purely imaginary. For negative time, *a* is purely imaginary $a=ic_{0}^{-\frac{1}{2}}\sqrt{\left|t+\gamma \right|}$. Then,

(171) $$i\Gamma =c_{0}^{-\frac{3}{2}}{\left|t+\gamma \right|}^{\frac{3}{2}}u_{t}^{\left(-\right)}\partial_{t}u_{t}^{\left(-\right)}$$

is a real function. We have checked, using Equation (155), that iΓ is negative (hence $\psi_{t}^{g}$ is square integrable) for the solution (155) when we obtain

$$ic_{0}^{\frac{3}{2}}{\left|t+\gamma \right|}^{-\frac{3}{2}}\Gamma =\frac{1}{2}{\left|t+\gamma \right|}^{-1}-\sqrt{c_{0}}k{\left|t+\gamma \right|}^{-\frac{1}{2}}\coth\left(2\sqrt{c_{0}}k\sqrt{-t-\gamma }\right)<0$$

For small |t+γ|, we have $i\Gamma ≃-\frac{5}{6}{\left|t+\gamma \right|}^{\frac{3}{2}}c_{0}^{-\frac{1}{2}}k^{2}$. There can be a smooth limit γ→0 of quantum scalar field theory when the metric passes from positive to negative signature. This happens if Γ in the WKB state (40) is determined by the classical wave function solution (153) (for t+γ>0) and (155) (for t+γ<0). $G_{t}$ is purely imaginary for a positive time, $G_{0}=0$ and $G_{t}$ becomes a real positively definite function for a negative time. For the exponential solutions, ℜiΓ is also negative, as discussed at Equation (168). We have obtained the dynamics of the fields $\Phi_{s}\left(x\right)$ in all cases (152)–(157). However, the limit γ→0 of the generate metric exists only for solutions (153) and (155). The solution (153) (for positive time) and its continuation to (155) (for negative time) are distinguished from all solutions of the wave equation as the corresponding Gaussian wave function being the WKB solution for positive time becomes a Gaussian normalizable wave function for the negative time. This behavior resembles the one in the elementary WKB approach when the wave function $\exp(\frac{i}{\hbar }\int dx\sqrt{2(E-V)}$, before a potential barrier takes the form $\exp(-\frac{1}{\hbar }\int dx\sqrt{2(V-E})$ inside the barrier, suggesting a tunneling process for the scalar field after crossing the classical barrier of an inverted signature.

If $a\left(t\right)≃{\left|t\right|}^{\frac{1}{2}}$, then the quantum scalar field evolution is unitary, and remains oscillatory, whereas for $a^{2}\left(t\right)≃t$, the oscillatory behavior for positive time becomes a diffusion at negative time as if the system encountered a barrier. In such a case, the evolution fails to be unitary. If we stopped the evolution at certain space-like surface according to the Hawking–Penrose singularity theorem [32], then unitarity would be violated as well. If, in the Lagrangian (22) and in the Hamiltonian (24), we replace $\sqrt{-g}$ by $\sqrt{\left|g\right|}$, then the resulting time evolution would resemble the one of an upside-down oscillator (it would be still unitary after an inversion of the signature).

Finally, we note that the correlation functions (157) are infinite if $2k\sqrt{c_{0}}\sqrt{t+\gamma }=\left(n+\frac{1}{2}\right)\pi$. In Equation (159), we obtain a pole at $2\sqrt{c_{0}}\sqrt{t+\gamma }k=n\pi$, where *n* is a natural number. So the fields $\Phi_{s}\left(\Phi \right)$ are well-defined for small $k\sqrt{t+\gamma }$. This difficulty already appears for a quantum mechanical oscillator of frequency *k* (see Appendix A). It means that the time evolution on the WKB states should be carefully extended from small values of time. The problem is connected with caustic singularities in semiclassical expansion [77].

## 11. Interaction with an Ultraviolet Cutoff

In this section, we discuss the Feynman–Kac formula for states of the WKB form $\exp(\frac{i}{\hbar }\Phi \Gamma \Phi )\chi$, where Γ is a real bilinear form defined by a real solution of the wave equation. In models of Section 6, the real solution *u* (leading to a real Γ) is a real Bessel function or the mode function in the expansion in spherical functions in Section 5. In [36], we have shown in quantum mechanics and in QFT in the Minkowski space-time that if the first (WKB) term on the rhs of Equation (44) is real, then $G_{ss^{\prime }}$ is purely imaginary (as it is for the inverted oscillator). In such a case for trigonometric or exponential interactions, the perturbation series is absolutely convergent. In this section, we consider the free fields of Section 3, Section 4, Section 5, Section 6, Section 7, Section 8, Section 9 and Section 10 and potentials of the form

(172) $$V\left(\Phi \right)=\lambda \int_{B}dx\sqrt{\left|g\right|}d\mu \left(\alpha \right):\exp\left(i\alpha \Phi_{s}\left(x\right)\right):,$$

where μ is a complex measure, $B\subset R^{3}$ is a bounded domain, :−: denotes the normal ordering, α can be real or purely imaginary, and χ is of the form

(173) $$\chi \left(\Phi \right)=\int d\nu \left(\alpha_{0}\right)\exp\left(i\alpha_{0}\left(f,\Phi \right)\right)$$

with $f\in L^{2}\left(dx\right)$. The exponential model (α imaginary) appears as Starobinski model for inflation [78] and the trigonometric interaction as the model of natural inflation [79].

We discuss also polynomial interactions of the form

(174) $$V\left(\Phi \right)=\lambda \int_{B}dx\sqrt{\left|g\right|}:\Phi_{s}^{N}:\left(x\right),$$

where N=4n and *n* is a natural number. In the latter case, we consider the Feynman–Kac solution for the holomorphically extended initial wave function $\psi (\sqrt{i}\Phi )$ (such states in the Feynman integral have been discussed first in [34,80]). Extensions of the wave functions (a complex scaling) are studied in the theory of resonances [81,82]. If in the expanding flat metric (67), we put the scalar field in a spatial box of length *L*, then k is discrete $k=\frac{2\pi n}{L}$, with $n=(n_{1},n_{2},n_{3})$, where $n_{j}$ are integers. For a small time, the Feynman formula will be well-defined if we restrict the range of n. First, we consider general formulas without specifying the number of k modes or $\Phi_{lm}$ modes (59). Then, we explain why a restriction to a finite number of modes (or an ultraviolet cutoff) is necessary in the case of the pseudoRiemannian metric.

The solution of the Schrödinger equation with the potential (172) (positive time, a bounded region *B*) reads

(175) $$\begin{array}{c} \psi_{t}\left(\sqrt{i}\Phi \right)=\psi_{t}^{g}\left(\sqrt{i}\Phi \right)E[\exp(-\lambda \frac{i}{\hbar }\int_{B}dx\int_{0}^{t}dsa^{3}\exp\left(iN\frac{\pi }{4}\right)\\ :{\left(u_{t-s}u_{t}^{-1}\Phi +\sqrt{\hbar }u_{t-s}\int_{0}^{s}{\left(a^{\frac{3}{2}}u\right)}^{-1}\left(t-\tau \right)dW_{\tau }\right)}^{N}:)\chi \left(\sqrt{i}\Phi_{t}\left(\Phi \right)\right)] \end{array}$$

In Equation (175), a necessary and sufficient condition for the stochastic integral (82) to be well-defined is that the integral (112) for $G_{t}$ exists (this is equivalent to $G_{ss^{\prime }}$ being finite). With the processes of Section 10, this requirement can be achieved (because of the caustic poles [77]) only for a small time if we have a finite number of modes or an ultraviolet cutoff κ (k<κ), so that tκ is sufficiently small. For a free field, the ultraviolet cutoff can be introduced by a restriction of states χ in Equation (108) as Fourier transforms to Λ, which have their support on |k|<κ. Another way is to introduce in the stochastic Equation (80), and in Equation (175), the ultraviolet regularized Brownian motion $W_{s}^{\kappa }\to W_{s}$ with κ→∞. The regularized Brownian motion is defined by the covariance

(176) $$E\left[W_{s}^{\kappa }\left(k\right)W_{t}^{\kappa }\left(k^{\prime }\right)\right]=min\left(s,t\right)\delta \left(k+k^{\prime }\right)\rho_{\kappa }\left(k\right),$$

where ρ is an ultraviolet cutoff restricting *k* to k≤κ ($\rho_{\kappa }\left(k\right)\to 1$ when κ→∞). If N=4n, then the exponential in Equation (175) is bounded by 1. In such a case,

(177) $$\begin{array}{c} |\psi_{t}^{\kappa }\left(\sqrt{i}\Phi \right)|\leq E\left[|\psi (\sqrt{i}\Phi_{t}\left(\Phi \right))|\right] \end{array}$$

The rhs of Equation (177) is finite for a large class of functions (e.g., the ones of Equation (173)). A renormalization of the polynomial interaction (175) is required if the perturbation expansion in the coupling constant λ is to be finite.

For the trigonometric interaction (172) an expansion of the exponential in Equation (46) (inside E[..]) leads to integrals $d\mu \left(\alpha_{j}\right)d\nu \left(\alpha_{0}\right)$ of functions of the form

(178) $$\begin{array}{c} \frac{\lambda^{n}}{n!}\int ds_{1}\ldots ds_{n}dx_{1}\ldots dx_{n}E[:\exp\left(i\alpha_{1}\Phi_{s_{1}}\left(x_{1}\right)\right):\ldots .:\exp\left(i\alpha_{n}\Phi_{s_{n}}\left(x_{n}\right)\right):\\ \times \exp(i\alpha_{0}\int dxf\left(x\right)\Phi_{t}\left(x\right))]. \end{array}$$

The expectation value (178) is (j≠r because of the normal ordering)

(179) $$\begin{array}{c} \exp\left(-\frac{1}{2}\sum_{j\neq r}\alpha_{j}\alpha_{r}\int dk\exp\left(ik\left(x_{j}-x_{r}\right)\right)G_{s_{j}s_{r}}\left(k\right)\right) \end{array}$$

times functions depending on the initial value Φ

(180) $$\exp\left(i\sum_{j}\alpha_{j}\left(u_{t-s_{j}}u_{t}^{-1}\Phi \right)\left(x_{j}\right)+i\alpha_{0}\int dx\left(u_{0}u_{t}^{-1}\Phi \right)\left(x\right)f\left(x\right)\right)$$

Most explicitly, the problem with the interaction in the Feynman–Kac Formula (46) appears in the model of Section 10, where $G_{ss^{\prime }}$ has been calculated exactly. For a finite number of modes and small $s_{j}<t$, the covariance $G_{s_{j},s_{r}}\left(k\right)$ is well-defined (as seen in Equations (157) and (180)) if the modes satisfy $2k\sqrt{c_{0}}\sqrt{t+\gamma }<\frac{\pi }{2}$. The Formulas (175) and (178) do not extend to arbitrary time and arbitrarily large *k*, because the Formula (157) gives an infinite $G_{ss^{\prime }}\left(k\right)$ if $2k\sqrt{c_{0}}\sqrt{t+\gamma }=\left(n+\frac{1}{2}\right)\pi$ (a pole at this value of *k*). In Equation (159), we obtain a similar pole at $2\sqrt{c_{0}}\sqrt{t+\gamma }k=n\pi$, where *n* is a natural number. However, the Lebesgue integral over $s_{j}$ and $\alpha_{j}$ in Equation (178) exists for a finite number of modes if $G_{t}$ is purely imaginary, even if *k* is large so that the trigonometric functions have poles. This is a consequence of the Lebesgue theorem saying that the Lebesgue integral of a bounded function with singularities on a set of Lebesgue measure zero exists. We may hope that, after an integration over $s_{j}$ and $\alpha_{j}$, we can go to the limit of an infinite number of modes.

In de Sitter space in the massless case (M=0), we can obtain real solutions $J_{\nu }$ and $Y_{\nu }$ of the wave equation. With a finite number of modes for a small time, we can define the interaction (172) in the Feynman–Kac formula in de Sitter space-time. The expression (179) will again be a pure phase factor (bounded by 1). We are unable to calculate $G_{ss^{\prime }}$ explicitly in this case. However, we expect that an integral of oscillating functions defining $G_{ss^{\prime }}$ will again show caustic singularities. Such caustic poles appear already in the evolution kernel (the Mehler formula for an oscillator) of free field theory. For a free field, even though the Mehler kernel (see Appendix A) has the caustic singularities at $t=\frac{\pi }{\omega }\left(n+\frac{1}{2}\right)$, the calculation of expectation values in the ground state gives correlation functions, which have an extension to an arbitrary time (this may be a consequence of the fact that the ground state is time-independent). We do not know whether such an extension of correlation functions is possible in the models of Section 8, Section 9 and Section 10. In Section 6 and Section 8, we have discussed complex *u*, leading to an integrable $|\psi_{t}{|}^{2}$. Such a wave function *u* defines the free quantum field in de Sitter space-time and in the universe expanding as ${\left|t\right|}^{\alpha }$. The correlation functions in such states show no caustic poles. In particular, we can construct de Sitter invariant correlation functions taking the Hankel function $H_{\nu }$ as the solution of the wave Equation (see Equation (90)).

In the next section, we show that the inversion of the signature which removes the caustics allows to define the Feynman integral without the ultraviolet cutoff (or the restriction on the number of modes).

## 12. Inverted Metric without an Ultraviolet Cutoff

In [36], we have discussed the models (172)–(174) (see Appendix C and Appendix E in this paper) in the Minkowski space-time. We have obtained a convergent perturbation series by an inversion of the signature of ${\left(\nabla \Phi \right)}^{2}$ in the Lagrangian (3) (but in contradistinction to Euclidean quantum field theory the time remains real). In the models of Section 8, Section 9 and Section 10, the inversion of the signature means $k^{2}\to -k^{2}$ in Equation (68) or k→ik.

In Section 11, we have shown that if Γ is real, then we have a well-defined Feynman formula for a finite number of modes (or an ultraviolet cutoff) and a small time *t*. In this section, we discuss the Feynman integral for fields on a manifold with an inverted (Euclidean) metric in the interpretation (27). Then, we have the Schrödinger equation for t>0, as well as t<0 and $G_{ss^{\prime }}$ which is purely imaginary and without the caustic poles, hence the model similar to that discussed in [36]. We obtain such a Feynman integral if, in the integral over metrics (with the interpretation of $\sqrt{-g}$ as $\sqrt{\left|g\right|}$ for an inverted metric), there appear stationary points (as solutions of Einstein equations) with an inverted signature. The stationary point may appear as the four-sphere (in addition to the de Sitter space of Section 6), as the metric $a^{2}≃-t^{2}$ in the coasting cosmology of Section 9, and in the background $a^{2}≃t$ of Section 10, if the Feynman integral is considered for negative time. With the inverted signature, there are no caustics in $G_{ss^{\prime }}$. If the term $\sqrt{-g}$ in the Feynman integral (or in the Hamiltonian (24)) is replaced by $\sqrt{\left|g\right|}$ (or if the inversion of signature is considered as a technical tool on an intermediate stage of the construction of quantum fields), then $G_{ss^{\prime }}$ will be purely imaginary. In such a case, we can apply the Feynman formula of Section 11 to trigonometric and $\Phi^{4n}$ interactions with an arbitrarily large *t* and without an ultraviolet cutoff (owing to the bound (177)). The ultraviolet limit exists, although the terms in the perturbation expansion (in λ in Equation (175) and in α in Equation (172)) are divergent. Note that the Formula (175) is well-defined for an arbitrarily large time with an ultraviolet cutoff, ensuring that the functions in the exponential are bounded. Because of the bound (177), we may apply the Lebesgue dominated convergence theorem to claim that the limit κ→∞ with $\rho_{\kappa }\left(k\right)\to 1$ exists. The interaction $\Phi^{4}$ deserves a detailed studies because of its role in the standard model. A renormalization of the interaction is necessary if the perturbation series in λ is to be finite. The counterterms for $\Phi^{4}$ are the same as in the conventional perturbation expansion because the ultraviolet singularity of the stochastic field is the same as the one of the quantum field.

We discuss the removal of regularization in more detail in the case of the trigonometric interactions (172). Using the representation (172) and (173), we calculate the expectation value over the Brownian motion $W_{s}^{\kappa }$ of the n-th order term in the perturbation expansion. We obtain a perturbation series of the form

(181) $$\begin{array}{c} \lambda^{n}\frac{1}{n!}\int \prod_{r}ds_{r}dx_{r}A\left(\Phi \right)\exp\left(-\sum_{j\neq r}\frac{1}{2}\alpha_{j}\alpha_{r}G_{s_{j},s_{r}}^{\kappa }\left(x_{j},x_{r}\right)\right), \end{array}$$

where A(Φ) is a bounded function depending on the initial value Φ of the field $\Phi_{s}$. The exponential factor in Equation (181) is a pure phase as the covariance $G_{s,s^{\prime }}^{\kappa }$ is purely imaginary. As a consequence, the perturbation series is absolutely convergent if ∫d|μ|<∞. In the model of Section 10 with the interpretation (27) of the Hamiltonian for the negative time (when $a^{2}<0$), there will be no caustics poles so that $G_{s_{j},s_{r}}\left(x_{j},x_{r}\right)$ (after the removal of the ultraviolet cutoff) is well-defined outside the coinciding points, which have the zero Lebesgue measure $ds_{1}dx_{1}\ldots \ldots ds_{n}dx_{n}$. As a consequence of the Lebesgue theorem, the integral (181) exists also after the removal of the ultraviolet cutoff. In the model of Section 9 with ϵ=−1, the function $G_{ss^{\prime }}\left(k\right)$ is non-singular as a function of k. The Fourier transform of $G_{ss^{\prime }}\left(k\right)$ in Equation (179) is singular when $\left(s_{j},x_{j}\right)\to \left(s_{r},x_{r}\right)$, because $G_{ss^{\prime }}\left(k\right)$ does not fall fast enough for large *k*. So, $G_{ss^{\prime }}\left(x_{j}-x_{r}\right)$ is divergent at the coinciding points $\left(s,x_{j}\right)\to \left(s^{\prime },x_{r}\right)$. This is the reason (as discussed at the end of Section 2) that, in the Euclidean space, the non-polynomial interactions cannot be defined. In the Minkowski space-time, the two-point function is a complex distribution singular on the light-cone, so that the Formula (181) would be untractable [71]. However, with the real time and the inverted metric, the exponential in Equation (181) is a pure phase bounded by 1. The integral over the singular points can be treated by means of the Lebesgue lemma. According to the Lebesgue lemma, an integral of a bounded function with singularities on a set of measure zero exists. When we consider an ultraviolet regularized model (as in Section 11) then, applying the Lebesgue dominated convergence theorem, we can conclude that, in the limit κ→∞, the expression (178) is integrable and the integrals are bounded by ${\left|\lambda \right|}^{n}{\left|B\right|}^{n}t^{n}\frac{1}{n!}$, because the exponential (181) is a pure phase.

An analogous formula can be derived in the (Euclidean) de Sitter space in the metric (56) and the interpretation (27) of the Hamiltonian. In such a case, the process is generated by the Hamiltonian (61). Then, instead of the Formula (181), we obtain (where $\omega_{j}$ are coordinates on $s^{3}$)

(182) $$\begin{array}{c} \int \prod_{j}ds_{j}d\omega_{j}\exp\left(-\frac{1}{2}\sum_{j\neq r}\alpha_{j}\alpha_{r}\int \sum_{lm}Y_{lm}\left(\omega_{r}\right)Y_{lm}{\left(\omega_{j}\right)}^{*}G_{s_{j}s_{r}}^{lm}\right) \end{array}$$

$G_{s_{j}s_{r}}^{lm}$ is purely imaginary and without caustic singularities. The infinite sum in the exponential (182) is convergent to a purely imaginary function singular at the coinciding points $\left(s_{j},\omega_{j}\right)$ and $\left(s_{r},\omega_{r}\right)$. It has the same singularity as the Green function on $S^{4}$. Hence, similarly as in Equation (181), we can prove the existence of the integrals and their non-triviality using Lebesgue lemma on dominated convergence.

In the metric (57), the analytic continuation of the metric to the Euclidean (58) leads to a field theory on AdS. As discussed at the end of Section 5 by means of the analytic continuation k→ik, we obtain a real solution u(k) of the “wave equation” on the Euclidean anti-de Sitter space. With the real *u*, the corresponding stochastic field has the $G_{ss^{\prime }}$ correlation function which is purely imaginary. In such a case, the expression (179) is integrable, defining the Euclidean quantum field theory on AdS.

## 13. Summary

We have developed a functional Schrödinger description of the time evolution of the scalar field in an external metric, which is a solution of Einstein equations. We do not restrict ourselves to the solutions with the Lorentzian signature, but also discuss solutions of Einstein equations with an Euclidean signature. Such a metric can be relevant when averaging the scalar field theory over all metrics in quantum gravity. In such a case, all saddle points contributing to the path integral over the scalar field should be taken into account. We work in the functional formulation of quantum field theory. Solutions of the Schrödinger equation define a random field whose correlation functions determine correlation functions of the quantum field. We consider Gaussian solutions of the Schrödinger equation for free field theory. The interaction is introduced by the Feynman–Kac formula. A proper choice of the Gaussian solution facilitates a definition of an integrable Feynman–Kac factor. We have shown that Gaussian solutions of the Schrödinger equation for a free field theory are determined by the classical solutions of the wave equation in an external metric. We studied in detail fields on de Sitter space-time and on the flat expanding space-time. The stochastic field correlation functions in a flat expanding space-time can be expressed by well-known cylinder functions, whereas the ones in de Sitter space require infinite series of Legendre functions. We have found a particular solution in a radiation background, which is of the WKB form (a Gaussian phase factor) for a positive time and a Gaussian square integrable function for a negative time (one may consider it as composed from two solutions for positive and negative time). This is an analog of the Gibbons–Hartle–Hawking solution in de Sitter space-time, which is glued from a solution for a positive time and another one for an imaginary time (positive and negative signature). We construct a general solution of the Schrödinger equation as a perturbation of the Gaussian (WKB) solution. We show how to calculate correlation functions of quantum fields in terms of correlation functions of stochastic fields. Then, the Feynman–Kac formula can be applied to construct solutions of the Schrödinger equation with an interaction. We discuss polynomial and trigonometric interactions. It is shown that the WKB solutions determined by real solutions of the classical wave equation (with an inverted signature) are distinguished from the point-of-view of the Feynman–Kac formula. The WKB solutions define stochastic fields, which allow for the definition of the Feynman–Kac factor as a bounded function. We briefly discussed the $\Phi^{4}$ interaction with a positive and negative coupling, important for the standard model and models of inflation. We pointed out that, with an inverted signature, an analytic continuation and the standard renormalization, the Feynman–Kac factor can be bounded and defined with finite renormalized perturbation series. This model requires further investigation. We studied, in detail, the trigonometric interaction showing that the perturbation series of the Schrödinger wave function evolution can be expressed by pure phase factors. The phase factors are well-defined for a small time and a finite number of modes in the Lorentzian metric, and for an arbitrary time with an infinite number of modes (no ultraviolet cutoff) in the case of the inverted metric with the Hamiltonian (27). Then, a further Lebesgue integration of these phase factors, which are singular on a set of zero Lebesgue measure, gives a finite result. This is in contradistinction to the standard Minkowski QFT of the trigonometric interaction, when the vacuum correlation functions are exponentials of unbounded complex functions (or distributions) which have infinite Lebesgue integrals. In this paper, a functional Schrödinger evolution has been derived for a scalar field. We shall extend this formulation to gauge fields when random self-duality equations will play the role of stochastic equations for the scalar field.

## Data Availability

Data are contained within the article.

## Appendix A. Expansion around a Time-Dependent Solution in Euclidean and Minkowski QFT

We consider a solution of Equation (17)

(A1) $$u_{t}=\cosh\left(\nu t\right),$$

where $\nu =\sqrt{-▵+M^{2}}$.

Then, $\Gamma_{t}=\nu \tanh\left(\nu t\right)$. The stochastic Equation (9) reads

(A2) $$d\Phi_{s}=-\nu \tanh\left(\nu \left(t-s\right)\right)\Phi_{s}ds+\sqrt{\hbar }dW_{s}.$$

With $u_{t}$ of Equation (A1),the solution of Equation (A2) is

(A3) $$\begin{array}{c} \Phi_{s}=\cosh\left(\nu \left(t-s\right)\right){\left(\cosh\left(\nu t\right)\right)}^{-1}\Phi \\ +\sqrt{\hbar }\cosh\left(\nu \left(t-s\right)\right)\int_{0}^{s}{\left(\cosh(\nu (t-\tau ))\right)}^{-1}dW_{\tau }. \end{array}$$

We can express the solution of Equation (6) in the form

$$\begin{array}{c} \chi_{t}\left(\Phi \right)=E\left[\chi \left(\Phi_{t}\left(\Phi \right)\right)\right]=\int d\Phi^{\prime }K_{t}\left(\Phi ,\Phi^{\prime }\right)\chi \left(\Phi^{\prime }\right), \end{array}$$

where

(A4) $$\begin{array}{c} K_{t}\left(\Phi ,\Phi^{\prime }\right)=E\left[\delta \left(\Phi^{\prime }-\Phi_{t}\left(\Phi \right)\right)\right]=\int d\Omega E[\exp\left(i\left(\Omega ,\Phi^{\prime }-\Phi_{t}\left(\Phi \right)\right)\right]. \end{array}$$

We calculate the expectation value (A4). For this purpose, we need

$$\begin{array}{c} E[\left(\int_{0}^{t}{\left(\Omega ,{\left(\cosh(\nu (t-\tau ))\right)}^{-1}dW_{\tau }\right)}^{2}\right]\\ =\int_{0}^{t}\left(\Omega ,{\left(\cosh(\nu (t-\tau ))\right)}^{-2}\Omega \right)d\tau =\left(\Omega ,\frac{1}{\nu }\tanh\left(\nu t\right)\Omega \right) \end{array}$$

In order to calculate the evolution kernel, we need to perform the Ω integral in Equation (A4) with the result (the Mehler formula for an imaginary time)

(A5) $$\begin{array}{c} K_{t}\left(\Phi ,\Phi^{\prime }\right)=\det{\left(\nu \coth(\nu t)\right)}^{\frac{1}{2}}\exp(-\frac{1}{2\hbar }\left(\Phi^{\prime }-{\left(\cosh\left(\nu t\right)\right)}^{-1}\Phi \right)\\ \times \nu \coth\left(\nu t\right)\left(\Phi^{\prime }{-(\cosh\left(\nu t\right)}^{-1}\Phi \right)) \end{array}$$

The formula is well-defined in an infinite number of dimensions. It also follows from the Mehler formula [83].

We can perform the whole procedure in the real-time t→it. Then, the stochastic Equation (44) reads

(A6) $$d\Phi_{s}=\nu \tan\left(\nu \left(t-s\right)\right)\Phi_{s}ds+\sqrt{i\hbar }dW_{s}$$

The formula for the evolution kernel (A5) takes the form

(A7) $$\begin{array}{c} K_{t}\left(\Phi ,\Phi^{\prime }\right)=\det{\left(i\nu \cot(\nu t)\right)}^{\frac{1}{2}}\\ \exp\left(\frac{i}{2\hbar }\left(\Phi^{\prime }-{\left(\cos\left(\nu t\right)\right)}^{-1}\Phi \right)\nu \cot\left(\nu t\right)\left(\Phi^{\prime }-{\left(\cos\left(\nu t\right)\right)}^{-1}\Phi \right)\right) \end{array}$$

In Equation (A7), we encounter the difficulty that $\cot{\left(\nu t\right)}^{-1}$ is infinite when $\nu \left(k\right)t=\left(n+\frac{1}{2}\right)\pi$. Equation (A7) can make sense for a small time if we introduce an ultraviolet cutoff restricting the range of *k*. Let us note that the ultraviolet cutoff is unnecessary if we flip the signature (but the time remains real)

$$\nabla^{2}\to -\nabla^{2}$$

together with $M^{2}\to -\mu^{2}$. In such a case in Equation (A7), ν→iν, cos(ωt)→cosh(νt) and νcot(νt)→νcoth(νt), where $\nu =\sqrt{-▵+\mu^{2}}$. Now, the stochastic Equation (A6) (real time but an inverted signature) reads

(A8) $$d\Phi_{s}=\nu \tanh\left(\nu \left(t-s\right)\right)\Phi_{s}ds+\sqrt{i\hbar }dW_{s}$$

with the solution

$$\Phi_{s}=\cosh\left(\left(t-s\right)\nu \right){\left(\cosh\left(t\nu \right)\right)}^{-1}\Phi +\sqrt{i\hbar }\cosh\left(\left(t-s\right)\nu \right)\int_{0}^{s}{\left(\cosh((t-\tau )\nu )\right)}^{-1}dW_{\tau }$$

We calculate $G_{ss^{\prime }}$ with the result

$$G_{ss^{\prime }}=i\hbar \cosh\left(\left(t-s\right)\nu \right)\cosh\left(\left(t-s^{\prime }\right)\nu \right)\nu^{-1}(\tanh\left(\nu \left(t-m\left(s,s^{\prime }\right)\right)-\tanh\left(\nu t\right)\right)$$

The propagator resulting from Equation (48) (this is the propagator for an upside-down oscillator discussed in more detail in Appendix C and Appendix D) is

(A9) $$\begin{array}{c} K_{t}\left(\Phi ,\Phi^{\prime }\right)=\det{\left(i\nu \coth(\nu t)\right)}^{\frac{1}{2}}\exp(\frac{i}{2\hbar }\left(\Phi^{\prime }-{\left(\cosh\left(\nu t\right)\right)}^{-1}\Phi \right)\nu \coth\left(\nu t\right)\\ \left(\Phi^{\prime }-{\left(\cosh\left(\nu t\right)\right)}^{-1}\Phi \right)) \end{array}$$

This model could be applied for a construction of a time evolution of trigonometric and $\Phi^{4n}$ interactions, as in Section 12 and [36] (where in [36] we used $\psi^{g}=\exp\left(\frac{i}{2\hbar }\Phi \nu \Phi \right)$ instead of $\psi^{g}=\exp\left(\frac{i}{2\hbar }\Phi \nu \tanh\left(\nu t\right)\Phi \right)$ considered in this appendix).

## Appendix B. Field Correlations and the Propagator at Large Momenta

We consider the wave equation

(A10) $$\frac{d^{2}u}{dt^{2}}+3H\frac{du}{dt}+a^{-2}k^{2}u+M^{2}u=0$$

at large *k*. Let $u=a^{-\frac{3}{2}}v$, then *v* satisfies the equation

(A11) $$\frac{d^{2}v}{dt^{2}}+\frac{dS}{dt}v=0$$

where

(A12) $$S\left(s\right)=\int_{0}^{s}dt\sqrt{M^{2}+a^{-2}k^{2}-\frac{3}{4}a^{-2}{\left(\frac{da}{dt}\right)}^{2}-\frac{3}{2}a^{-1}\frac{d^{2}}{dt^{2}}a}$$

The field correlation function

$$\begin{array}{c} G_{s,s^{\prime }}\left(k,k^{\prime }\right)=E\left[\left(\Phi_{s^{\prime }}\left(k\right)-<\Phi_{s^{\prime }}>\left(k\right)\right)\left(\Phi_{s}\left(k^{\prime }\right)-<\Phi_{s}>\left(k^{\prime }\right)\right)\right]\\ \equiv G_{s,s^{\prime }}\left(k\right)\delta \left(k+k^{\prime }\right) \end{array}$$

is

(A13) $$G_{s,s^{\prime }}\left(k\right)=i\hbar u_{t-s}u_{t-s^{\prime }}\int_{0}^{m}d\tau u_{t-\tau }^{-2}a_{t-\tau }^{-3}d\tau ,$$

where $m=min(s,s^{\prime })$.

In the large *k* (the WKB method), we obtain the approximate solution of Equation (A11)

(A14) $$v\left(t\right)={\left(\frac{dS(t)}{dt}\right)}^{-\frac{1}{2}}\exp\left(\pm iS\left(t\right)\right).$$

We can form the even solution

(A15) $$v\left(t\right)={\left(\frac{dS(t)}{dt}\right)}^{-\frac{1}{2}}\cos\left(S\left(t\right)\right).$$

In the odd solution, we replace cos by sin.

With the inverted metric (and $M^{2}\to -\mu^{2}$)

(A16) $$S^{E}\left(s\right)=\int_{0}^{s}dt\sqrt{\mu^{2}+a^{-2}k^{2}+\frac{3}{4}a^{-2}{\left(\frac{da}{dt}\right)}^{2}+\frac{3}{2}a^{-1}\frac{d^{2}}{dt^{2}}a}$$

Then, the even solution is

(A17) $$v^{E}\left(t\right)={\left(\frac{dS^{E}\left(t\right)}{dt}\right)}^{-\frac{1}{2}}\cosh\left(S^{E}\left(t\right)\right)$$

and the exponentially growing solution

(A18) $$v^{E}\left(t\right)={\left(\frac{dS^{E}\left(t\right)}{dt}\right)}^{-\frac{1}{2}}\exp\left(S^{E}\left(t\right)\right)$$

With $u=a^{-\frac{3}{2}}v$, we can calculate $G_{s,s^{\prime }}\left(k\right)$ from Equation (A13) for the even solution, for the Euclidean even solution, and for the Euclidean exponentially growing solutions. The results are expressed by Equations (105)–(107), where we skip the indices lm. So for the Euclidean exponentially growing solution, we obtain ($u^{E}=a^{-\frac{3}{2}}v^{E}$)

(A19) $$\begin{array}{c} G_{s,s^{\prime }}=\hbar u^{E}\left(\tau -s\right)u^{E}\left(\tau -s^{\prime }\right)(\exp(-2S^{E}\left(\tau -m\left(s,s^{\prime }\right)\right)-\exp\left(-2S^{E}\left(\tau \right)\right) \end{array}$$

For a large *k*, the correlation $G_{s,s^{\prime }}$ behaves as $A\left(s,s^{\prime }\right)k^{-1}\exp\left(-B\left(s,s^{\prime }\right)k\right)$ with certain functions *A* and *B*. Hence, it has the same short distance behavior as the two-point function of the Euclidean free scalar field (see Equation (140)).

## Appendix C. The Oscillator with an Inverted Signature in Quantum Mechanics

For the convenience of the reader, we briefly discuss the results of [36] in this appendix and Appendix E, which constitute simplified version of the models considered in this paper. We consider the Schrödinger equation in quantum mechanics

(A20) $$i\hbar \partial_{t}\psi_{t}=\left(-\frac{\hbar^{2}}{2}\nabla_{x}^{2}-\frac{\nu^{2}x^{2}}{2}+\tilde{V}\left(x\right)\right)\psi_{t}\equiv {\hat{H}}_{0}+\tilde{V}\psi_{t}.$$

We write the solution of the Schrödinger Equation (A20) in the form

(A21) $$\psi_{t}\left(x\right)=\psi_{t}^{g}\chi =\exp\left(-\frac{\nu }{2}t\right)\exp\left(i\frac{\nu x^{2}}{2\hbar }\right)\chi_{t}\left(x\right).$$

We express the solution by the Brownian motion.

For the wave function $\psi_{t}^{g}=\exp\left(-\frac{\nu }{2}t\right)\exp\left(\frac{i\nu }{\hbar }x^{2}\right)$, the stochastic Equation (44) reads

(A22) $$dq_{s}=-\nu qds+\sqrt{i\hbar }dw_{s}.$$

We assume that the initial wave function χ and the potential $\tilde{V}$ are holomorphic functions. Then, the solution of Equation (A20) is given by the Feynman–Kac formula

(A23) $$\chi_{t}\left(x\right)=E\left[\exp\left(-\frac{i}{\hbar }\int_{t_{0}}^{t}ds\tilde{V}\left(q_{s}\left(x\right)\right)\right)\chi \left(q_{t}\left(x\right)\right)\right],$$

here, $q_{s}\left(x\right)$ is the solution of the Langevin Equation (A22) with the initial condition $q_{t_{0}}\left(x\right)=x$. The solution (A23) has been discussed earlier in [17,19,20,21,34]. It is a real-time version of the Feynman–Kac formula [5,38].

We define the evolution kernel (for $\tilde{V}=0$) as

(A24) $$\begin{array}{c} \exp\left(-\frac{\nu t}{2}+\frac{i\nu x^{2}}{2}\right)E\left[\chi \left(q_{t}\left(x\right)\right)\right]=\int K\left(t;x,y\right)\exp\left(\frac{i\nu y^{2}}{2}\right)\chi \left(y\right)dy=\left(U_{t}\psi \right)\left(x\right) \end{array}$$

leading to the result (the Mehler formula for the evolution kernel of an oscillator with ω→iν) [83])

(A25) $$\begin{array}{c} K\left(t;x,y\right)=\exp\left(t{\hat{H}}_{0}\right)\left(x,y\right)={\left(2\pi \hbar i\nu^{-1}\sinh\left(\nu t\right)\right)}^{-\frac{1}{2}}\\ \exp\left(\frac{i\nu }{2\hbar \sinh(\nu t)}\left(\left(x^{2}+y^{2}\right)\cosh\left(\nu t\right)-2xy\right)\right). \end{array}$$

It is related to the kernel (A9) through a similarity transformation by means of the WKB factor $\exp(\frac{i}{2\hbar }x\nu x)$.

We consider potentials of the form of the Fourier transforms of a complex measure [18,84]

(A26) $$\tilde{V}\left(x\right)=g\int d\mu \left(a\right)\exp\left(iax\right),$$

and wave functions of the same form

(A27) $$\psi \left(x\right)=\int d\rho \left(a_{0}\right)\exp\left(ia_{0}x\right),$$

where a∈R.

We prove that the solution of Equation (A20) can be expressed as a convergent perturbation series if ∫d|μ|<∞ and ∫d|ρ|<∞

(A28) $$\begin{array}{c} \chi_{t}\left(x\right)=E\left[\sum_{n}\frac{1}{n!}{\left(-\frac{i}{\hbar }\int_{t_{0}}^{t}\tilde{V}\left(q_{s}\right)ds\right)}^{n}\chi_{0}\left(q_{t}\left(x\right)\right)\right], \end{array}$$

We show that the perturbation series (A28) in powers of $\tilde{V}$ is absolutely convergent. With the Fourier representation (A26), we can see that the *N*-th order term is of the form (a simpler version of Equation (181))

(A29) $$\begin{array}{c} \int \prod_{r}d\mu \left(a_{r}\right)\exp\left(\sum_{j,k}f\left(a_{j},a_{k}\right)\right)\\ \exp\left(-\frac{1}{2}i\hbar \sum_{j,k}a_{j}a_{k}\int_{0}^{min(s_{j},s_{k})}\exp\left(-\nu \left(s_{j}+s_{k}-2s\right)\right)ds\right), \end{array}$$

where

(A30) $$f\left(a_{j},a_{k}\right)=i\alpha a_{j}x\exp\left(-\nu s_{j}\right)+i\alpha a_{k}x\exp\left(-\nu s_{k}\right).$$

It is clear that absolute values of the terms (A29) integrated over *s* are bounded by 1, leading to a convergent perturbation expansion in which each term (A28) is bounded by $\frac{1}{n!}t^{n}C^{n}$ with a certain positive constant *C*.

## Appendix D. QFT in a Formal ℏ Expansion

We calculate the generating functional in a formal expansion in *ℏ* (up to the $O(\sqrt{\hbar })$ terms) for the Lagrangian (22) with an inverted signature of the spatial metric in the Minkowski space-time, and an inverted sign of the mass square $M^{2}\to -\mu^{2}$

(A31) $$\begin{array}{c} Z\left[J\right]=\int d\phi \exp\left(\frac{i}{\hbar }\int dx\left(L+J\phi \right)\right)=\exp\left(\frac{i}{\hbar }\int dx\left(L\left(\phi_{c}\right)+J\phi_{c}\right)\right)\\ \det{\left(i(-\partial_{t}^{2}-\nabla^{2}+\mu^{2}-{\tilde{V}}^{\prime \prime }\left(\phi_{c}\right))\right)}^{-\frac{1}{2}}, \end{array}$$

where

$$L=\frac{1}{2}\left({\left(\partial_{t}\phi \right)}^{2}+{\left(\nabla \phi \right)}^{2}+\mu^{2}\phi^{2}\right)-\tilde{V}\left(\phi \right)$$

and $\phi_{c}\left(t,x\right)\equiv \phi_{t}^{c}\left(x\right)$ is the solution of the equation

(A32) $$\left(-\partial_{t}^{2}-\nabla^{2}+\mu^{2}\right)\phi_{c}-{\tilde{V}}^{\prime }\left(\phi_{c}\right)=-J.$$

For the propagator, we have the expression

(A33) $$\begin{array}{c} K\left(t;\phi ,\phi^{\prime }\right)=\int_{\phi_{0}=\phi ,\phi_{t}=\phi^{\prime }}d\phi \exp\left(\frac{i}{\hbar }\int dxL\right)=\exp\left(\frac{i}{\hbar }\int dxL\left(\phi_{c}\right)\right)\\ \times \det{\left(i(-\partial_{t}^{2}-\nabla^{2}+\mu^{2}-{\tilde{V}}^{\prime \prime }\left(\phi_{c}\right))\right)}^{-\frac{1}{2}}, \end{array}$$

where

(A34) $$\left(-\partial_{t}^{2}-\nabla^{2}+\mu^{2}\right)\phi_{c}-{\tilde{V}}^{\prime }\left(\phi_{c}\right)=0.$$

Equation (A34) is solved with the boundary conditions $\phi_{0}^{c}=\phi ,\phi_{t}^{c}=\phi^{\prime }$. Equations (A31) and (A33) have a form similar to the ones in Euclidean field theory, but the potential enters with the opposite sign.

For $\tilde{V}=0$, we can obtain explicit formulae from Equations (A31)–(A34)

(A35) $$Z\left[J\right]=\exp\left(-\frac{1}{2\hbar }JGJ\right),$$

where

(A36) $$\begin{array}{c} G\left(t,x;t^{\prime },x^{\prime }\right)=i\left(\exp(-\nu |t-t^{\prime }\left|\right){\left(2\nu \right)}^{-1}\right)\left(x,x^{\prime }\right)\equiv iG^{E}\left(t,x;t^{\prime },x^{\prime }\right) \end{array}$$

where $G^{E}$ is the two-point function for the Euclidean free field (with $\nu =\sqrt{-▵+\mu^{2}}$).

In the expanding flat metric with no potential, the formula for the evolution kernel reads

(A37) $$\begin{array}{c} K\left(t;\phi ,\phi^{\prime }\right)=\int_{\phi_{0}=\phi ,\phi_{t}=\phi^{\prime }}d\phi \exp\left(\frac{i}{\hbar }\int dx\sqrt{-g}L\right)=\exp\left(\frac{i}{\hbar }\int dx\sqrt{-g}L\left(\phi_{c}\right)\right)\\ \times \det{\left(i(-\partial_{t}^{2}-a^{-2}\nabla^{2}-3a^{-1}\partial_{t}a\partial_{t}+\mu^{2})\right)}^{-\frac{1}{2}}, \end{array}$$

where

(A38) $$\left(-\partial_{t}^{2}-\nabla^{2}-3a^{-1}\partial_{t}a\partial_{t}+\mu^{2}\right)\phi_{c}=0$$

is solved with the boundary conditions $\phi_{0}^{c}=\phi ,\phi_{t}^{c}=\phi^{\prime }$. We would obtain the form of the kernel (111) if we could solve the boundary problem (A38) explicitly. The determinant (for a given a(t)) depends only on time. Then, $\exp\left(\frac{i}{\hbar }\int dxL\left(\phi_{c}\right)\right)$ gives the quadratic form in the exponential of Equation (111). This way of calculating the evolution kernel is discussed in [62] (without an inversion of the metric).

## Appendix E. Feynman Integral in QFT of Trigonometric Interactions

An extension of Equations (A20)–(A22) to QFT takes the form (after a subtraction of the infinite vacuum energy)

(A39) $$\psi_{t}\left(\phi \right)=\exp\left(\frac{i}{2\hbar }\phi \nu \phi \right)E\left[\chi \left(\phi_{t}\left(\phi \right)\right)\right]$$

where

(A40) $$\phi_{t}\left(t_{0},\phi \right)=\exp\left(-\nu \left(t-t_{0}\right)\right)\phi +\sqrt{i\hbar }\int_{t_{0}}^{t}\exp\left(-\nu \left(t-s\right)\right)dW_{s},$$

where $\nu =\sqrt{-▵+\mu^{2}}$.

From Equation (A40), we obtain the correlation function in field theory as

(A41) $$\begin{array}{c} E[\phi_{t}\left(\phi ,y\right)\phi_{s}\left(\phi ,x\right)]\\ =\left(\exp\left(-\left(t-t_{0}\right)\nu \right)\phi \right)\left(y\right)\left(\exp\left(-\left(s-t_{0}\right)\nu \right)\phi \right)\left(x\right)\\ +\left(\frac{1}{2\nu }\exp\left(-\nu \left(t+s-2t_{0}\right)\right)\right)\left(x,y\right)+G\left(t,y;s,x\right) \end{array}$$

with

(A42) $$\begin{array}{c} G\left(t,y;s,x\right)=i{\left(-\partial_{0}^{2}-▵+\mu^{2}\right)}^{-1}\left(t,y;s,x\right)\\ =\frac{i}{2}\left(\nu^{-1}\exp\left(-\nu |t-s|\right)\right)\left(x,y\right)=iG^{E}\left(t,y;s,x\right), \end{array}$$

where $G^{E}$ is the two-point function of Euclidean free quantum field. The Feynman–Kac formula reads

(A43) $$\begin{array}{c} \psi_{t}\left(\phi \right)=\exp\left(\frac{i}{2\hbar }\phi \nu \phi \right)\\ E\left[\exp\left(-\frac{i}{\hbar }\int_{t_{0}}^{t}:\tilde{V}\left(\phi_{s}\left(\phi ,x\right)\right):dxds\right)\chi \left(\phi_{t}\left(\phi \right)\right)\right]. \end{array}$$

For the exponential interaction (172), the *n*-th order term has the form

(A44) $$\begin{array}{c} \int_{\Omega }dx_{1}\ldots \ldots dx_{n}ds_{1}\ldots ds_{n}d\mu \left(a_{1}\right)\ldots d\mu \left(a_{n}\right)\prod_{j\neq k}\\ \exp(-\frac{1}{2}i\hbar a_{j}a_{k}\int_{t_{0}}^{\min(s_{j},s_{k})}\\ \left(\exp\left(-\left(s_{j}+s_{k}\right)\right)\nu )\exp\left(2\tau \nu \right)\right)\left(x_{j},x_{k}\right)d\tau ). \end{array}$$

The absolute value of the integrand (A44) is 1, as in quantum mechanics in Appendix C (Equation (A29)) and in the models with an inversion of the metric in Section 12 (Equation (181)). The formulas of this appendix exhibit a simpler and more explicit version of the discussion of Section 11 and Section 12.

## Appendix F. De Sitter Space in the Cosmic Time

We obtain for the metric (51) the Hamiltonian (24)

(A45) $$\begin{array}{c} H=\frac{1}{2}\sum_{lm}\left({\left(\frac{1}{H}\cosh\left(Ht\right)\right)}^{-3}\Pi_{lm}^{2}+\frac{1}{H}\cosh\left(Ht\right)l\left(l+2\right)\Phi_{lm}^{2}+M^{2}{\left(\frac{1}{H}\cosh\left(Ht\right)\right)}^{3}\Phi_{lm}^{2}\right) \end{array}$$

defining for t≥0 the Schrödinger Equation (25). The Hamiltonian for the Euclidean metric (54) in the interpretation (27) is (we change $M^{2}\to -\mu^{2}$)

(A46) $$\begin{array}{c} H=\frac{1}{2}\sum_{lm}\left({\left(\frac{1}{H}\cos\left(Ht\right)\right)}^{-3}\Pi_{lm}^{2}-\frac{1}{H}\cos\left(Ht\right)l\left(l+2\right)\Phi_{lm}^{2}-\mu^{2}{\left(\frac{1}{H}\cos\left(Ht\right)\right)}^{3}\Phi_{lm}^{2}\right) \end{array}$$

In the interpretation (26), when $\sqrt{-g}$ is imaginary, then we have the diffusion equation $\hbar \partial_{t}\psi =H_{E}\psi$ with

(A47) $$\begin{array}{c} H_{E}=\frac{1}{2}\sum_{lm}\left({\left(\frac{1}{H}\cos\left(Ht\right)\right)}^{-3}\Pi_{lm}^{2}+\frac{1}{H}\cos\left(Ht\right)l\left(l+2\right)\Phi_{lm}^{2}+\mu^{2}{\left(\frac{1}{H}\cos\left(Ht\right)\right)}^{3}\Phi_{lm}^{2}\right) \end{array}$$

The operator $H_{E}$ is positive. Hence, the diffusion equation should be considered for the negative time.

We expand the solution of the wave equation in spherical harmonics as in Section 5. Then, *u* satisfies the equation

(A48) $$\partial_{t}^{2}u_{lm}+3H\tanh\left(Ht\right)\partial_{t}u_{lm}+M^{2}u_{lm}+l\left(l+2\right){\left(\frac{1}{H}\cosh\left(Ht\right)\right)}^{-2}u_{lm}=0$$

and, in the Euclidean case (54),

(A49) $$\partial_{t}^{2}u_{lm}-3H\tan\left(Ht\right)\partial_{t}u_{lm}-\mu^{2}u_{lm}-l\left(l+2\right){\left(\frac{1}{H}\cos\left(Ht\right)\right)}^{-2}u_{lm}=0$$

The stochastic equation for the fields is

(A50) $$d\Phi_{lm}\left(s\right)=-u_{lm}^{-1}\partial_{t}u_{lm}\left(t-s\right)\Phi_{lm}\left(s\right)ds+\sqrt{i\hbar }{\left(\frac{1}{H}\cos\left(H\left(t-s\right)\right)\right)}^{-\frac{3}{2}}dw_{lm}\left(s\right)$$

and the one for the diffusion (26) is

(A51) $$d\Phi_{lm}^{E}\left(s\right)=-{\left(u_{lm}^{E}\right)}^{-1}\partial_{t}u_{lm}^{E}\left(t-s\right)\Phi_{lm}^{E}\left(s\right)ds+\sqrt{\hbar }{\left(\frac{1}{H}\cosh\left(H\left(t-s\right)\right)\right)}^{-\frac{3}{2}}dw_{lm}\left(s\right)$$

The two-dimensional de Sitter model is soluble in terms of elementary functions, by means of the methods developed in this paper (for the standard approach, see [60,61]). There are minor differences in comparison to Equations (A45)–(A47) resulting from the fact that $\sqrt{-g}={\left(\frac{1}{H}\cosh\left(Ht\right)\right)}^{3}\to \frac{1}{H}\cosh\left(Ht\right)$, because the three spatial dimensions are replaced by one dimension. So, Equations (A48) and (A49) are changed into

(A52) $$\partial_{t}^{2}u_{n}+H\tanh\left(Ht\right)\partial_{t}u_{n}+M^{2}u_{n}+n^{2}{\left(\frac{1}{H}\cosh\left(Ht\right)\right)}^{-2}u_{n}=0$$

and, in the Euclidean case,

(A53) $$\partial_{t}^{2}u_{n}-H\tan\left(Ht\right)\partial_{t}u_{n}-\mu^{2}u_{n}-n^{2}{\left(\frac{1}{H}\cos\left(Ht\right)\right)}^{-2}u_{n}=0$$

where *n* is a natural number. *n* is replacing l(l+2) as an eigenvalue of the Laplacian on $S^{1}$ (instead of the one on $S^{3}$). If we introduce the variable

y=tanh(Ht)

then Equation (A52) in the massless case reads

(A54) $$\left(y^{2}-1\right)\partial_{y}^{2}u+y\partial_{y}u-n^{2}u=0$$

For Equation (A53), we set

y=tan(Ht)

Then, Equation (A53) is

(A55) $$\left(y^{2}+1\right)\partial_{y}^{2}u+y\partial_{y}u-n^{2}u=0$$

The solution of Equation (A54) for de Sitter is the Tchebyshev polynomial $P_{n}$

(A56) $$u_{n}=z^{n}+z^{-n}=P_{n}\left(y\right)$$

where

(A57) $$z=y+i\sqrt{1-y^{2}}$$

Then, $G_{ss^{\prime }}$ can be expressed by elementary functions as

(A58) $$G_{ss^{\prime }}=i\hbar u\left(t-s\right)u\left(t-s^{\prime }\right)\int_{0}^{m(s,s^{\prime })}\frac{dy}{y}P_{n}^{-2}$$

The solution for the sphere (A55) for n=0 is lnz, where

(A59) $$z=y+\sqrt{1+y^{2}}$$

For n>0, we obtain a polynomial in *y*

(A60) $$u_{n}=z^{n}+{\left(-1\right)}^{n}z^{-n}={\tilde{P}}_{n}\left(y\right)$$

The diffusion resulting from the imaginary value of $\sqrt{-g}$ has the correlation

(A61) $$G_{ss^{\prime }}=\hbar u\left(t-s\right)u\left(t-s^{\prime }\right)\int_{0}^{m(s,s^{\prime })}\frac{dy}{y}{\tilde{P}}_{n}^{-2}$$

In the interpretation of the Hamiltonian with $\sqrt{-g}\to \sqrt{\left|g\right|}$, we would replace ℏ→iℏ.

We solve the homogenous model with a(t)=exp(Ht) of Equation (57). The wave equation reads

(A62) $$\partial_{t}^{2}u+H\partial_{t}u+k^{2}\exp\left(-2Ht\right)u=0$$

The solution, which gives a negative iΓ, is

(A63) $$u\left(t\right)=\exp(-i\frac{k}{H}\exp\left(-Ht\right))$$

Then,

(A64) $$G_{ss^{\prime }}=\frac{\hbar }{2k}u\left(t-s\right)u\left(t-s^{\prime }\right)\left(\exp\left(2i\frac{k}{H}\exp(-H\left(t-m\left(s,s^{\prime }\right)\right)\right)-\exp\left(2i\frac{k}{H}\exp\left(-Ht\right)\right)\right)$$

and

(A65) $$G_{t}=\frac{\hbar }{2k}\left(1-\cos\left(2\frac{k}{H}\left(\exp\left(-Ht\right)-1\right)\right)-i\sin\left(2\frac{k}{H}\left(\exp\left(-Ht\right)-1\right)\right)\right)$$

So that $ℜG_{t}>0$, proving that the propagator (111), defines an integrable function.

The inverted metric (58) describes the Euclidean version of the AdS field (the scalar free field on the Poincare upper half plane). The solution is

(A66) $$u^{E}=\exp\left(-\frac{k}{H}\exp\left(-Ht\right)\right)$$

Now,

(A67) $$G_{ss^{\prime }}=\frac{\hbar }{2k}u^{E}\left(t-s\right)u^{E}\left(t-s^{\prime }\right)\left(\exp\left(2\frac{k}{H}\exp(-H\left(t-m\left(s,s^{\prime }\right)\right)\right)-\exp\left(2\frac{k}{H}\exp\left(-Ht\right)\right)\right)$$

and

(A68) $$G_{t}=\frac{\hbar }{2k}\left(1-\exp\left(2\frac{k}{H}\left(\exp\left(-Ht\right)-1\right)\right)\right)>0$$

In summary, as follows from Equations (A58), (A61) and (A67), we can express the time evolution propagator of Section 7 in the two-dimensional massless de Sitter model by elementary functions. Then, with the trigonometric interaction of Section 12 (Equation (181)), we obtain a solution of the Schrödinger equation in the form of a convergent series of elementary functions.

## Appendix G. Scalar Field in *d* = 5 Dimension

We may ask the question of whether the signature inversion described in Section 10 on the background of radiation ($w=\frac{1}{3}$) in four dimensions can happen in other backgrounds. To discuss this question, we consider the Friedmann equation in *d* dimensional space-time. We assume p=wρ (where ρ is the energy density and *p* is the pressure). Then, from the energy-momentum conservation,

(A69) $$\rho =\rho_{0}a^{-(d-1)(1+w)}$$

As a consequence, the Friedmann equation in a flat expanding universe reads [85]

(A70) $${\left(\frac{da}{dt}\right)}^{2}=Ca^{-(d-1)(1+w)+2},$$

where *C* is a positive constant. If we insert $a^{2}=t$ in this equation, then we obtain the condition 4=(1+w)(d−1). Hence, for d>5, the index *w* in the equation of state must be negative. For a radiation background from the condition $T_{\mu }^{\mu }=0$, we obtain $w=\frac{1}{d-1}$. Hence, the condition $a^{2}=t$ for radiation is satisfied only in four dimensions. The signature would change when time is changing sign if $a^{2}=t^{2n+1}$, where *n* is a natural number. Then, Equation (A70) can be satisfied for a particular w>0, but in the interval −1≤w<0. There remains an interesting case of d=5 and w=0 (dust) when $a^{2}=t$. In five dimensions, *g* does not change its sign when *t* becomes negative. In such a case, we have a unitary evolution for positive as well as for a negative time with an inverted metric. The equation for χ takes the form

(A71) $$\partial_{t}\chi_{t}=\frac{1}{2}\int dx\left(i\hbar \frac{1}{2}c_{0}^{2}{\left(t+\gamma \right)}^{-2}\frac{\delta^{2}}{\delta \Phi {\left(x\right)}^{2}}-u^{-1}\partial_{t}u\Phi \left(x\right)\frac{\delta }{\delta \Phi (x)}\right)\chi_{t},$$

where *u* is the solution of the equation

(A72) $$\frac{d^{2}u}{dt^{2}}+2{\left(t+\gamma \right)}^{-1}\frac{du}{dt}+{\left(t+\gamma \right)}^{-1}c_{0}k^{2}u=0.$$

The solution can be expressed by the cylinder function [68]

(A73) $$u={\left(t+\gamma \right)}^{-\frac{1}{2}}Z_{1}\left(2\sqrt{c_{0}}k\sqrt{t+\gamma }\right)$$

We can choose the Bessel function as $Z_{1}$

$$J_{1}\left(z\right)=\frac{z}{2}\sum_{n=0}^{\infty }{\left(-1\right)}^{n}\frac{1}{n!(n+1)!}{\left(z^{2}\right)}^{n}$$

Then, *u* is defined for positive as well as for negative time. The stochastic Equation (for positive time) reads

(A74) $$d\Phi_{s}=-u^{-1}\partial_{t}u\left(t-s\right)\Phi_{s}ds+\sqrt{i\hbar }{\left(t+\gamma -s\right)}^{-1}dW_{s}$$

Then,

(A75) $$G_{ss^{\prime }}=i\hbar u\left(t-s\right)u\left(t-s^{\prime }\right)\int_{0}^{m(s,s^{\prime })}u^{-2}\left(t-\tau \right){\left(t+\gamma -\tau \right)}^{-2}d\tau$$

$G_{ss^{\prime }}$ is purely imaginary. We could construct interactions in 5 dimensions in the way we did in Section 11 and Section 12.

## Appendix H. Matter Field Schrödinger Evolution from Wheeler–DeWitt Equation

In this appendix, we would like to show how the Schrödinger equation in an external metric discussed in this paper can appear in quantum gravity as a consequence of (Wheeler–DeWitt) constraint on the canonical variables. The Wheeler–DeWitt [28,29] equation is derived as a Hamiltonian constraint resulting from the diffeomorphism invariance of the Einstein action

(A76) $$\begin{array}{c} \left(-c\hbar \nu_{p}^{-2}\int dxG_{ijkl}\frac{\delta^{2}}{\delta h_{ij}\delta h_{kl}}-c\hbar \nu_{p}^{2}\int dx\sqrt{h}\left(R-2\Lambda \right)+\int dxH\left(h,x\right)\right)\psi =0, \end{array}$$

where H(h,x) is the density of the scalar field Hamiltonian (24), Λ is the cosmological constant and

(A77) $$G_{ijkl}=\frac{1}{2}h^{-\frac{1}{2}}\left(h_{ik}h_{jl}+h_{il}h_{jk}-h_{ij}h_{kl}\right)$$

is a metric on the set of symmetric tensors. $h_{ij}$ is the metric on the spatial hyper-surface and H is the Hamiltonian (24).

$$\nu_{p}={\left(16\pi G\hbar c^{-3}\right)}^{-\frac{1}{2}}$$

is the inverse of the the Planck length (*G* is the Newton constant). The metric $G_{ijkl}$ does not have a definite sign. For a conformally flat metric $h_{ij}=a^{2}\delta_{ij}$, the metric $G_{ijkl}$ is negatively definite. In such a case, Equation (A76) becomes an equation of the hyperbolic type (a wave equation), which can be written in the form

(A78) $$\int dx\left(\frac{\delta^{2}}{\delta a(x)\delta a(x)}-\nu_{p}^{4}\frac{8}{3}a^{4}\left(R\left(a\right)-2\Lambda \right)+{\left(c\hbar \right)}^{-1}\nu_{p}^{2}\frac{8}{3}aH\left(a,x\right)\right)\psi \left(a,\phi \right)=0.$$

R(a) for a conformally flat metric depends on derivatives of *a*. We can solve Equation (A78) if we assume that R(a) can be expressed as a function of a(x) (without derivatives). As an example, this is possible if a(x) depends only on |x|. Then, we can express |x| by *a* and, subsequently, R(|x|) as R(a).

We obtain the WKB solution treating $\nu_{p}^{-2}$ as a small parameter

(A79) $$\psi_{a}=\exp\left(\pm i\nu_{p}^{2}S\left(a\right)\right)\psi_{a_{0}}.$$

with

(A80) $$\nu_{p}^{2}S\left(a\right)=\int dx\int_{a_{0}}^{a(x)}d\alpha \sqrt{-\frac{8}{3}\nu_{p}^{4}\alpha^{4}\left(R\left(\alpha \right)-2\Lambda \right)+\frac{8}{3}{\left(c\hbar \right)}^{-1}\nu_{p}^{2}\alpha H\left(\alpha ,x\right)}$$

Expanding the square root in Equation (A80) in powers of the Planck length $\nu_{p}^{-2}$, we obtain

(A81) $$\begin{array}{c} \nu_{p}^{2}S\left(a\right)=\int dx\int_{a_{0}}^{a(x)}d\alpha (\nu_{p}^{2}\sqrt{-\frac{8}{3}\alpha^{4}\left(R\left(\alpha \right)-2\Lambda \right)}\\ +\frac{4}{3}\alpha H\left(\alpha ,x\right){\left(c\hbar \right)}^{-1}{\left(-\frac{8}{3}\alpha^{4}\left(R\left(\alpha \right)-2\Lambda \right)\right)}^{-\frac{1}{2}})+O\left(\nu_{p}^{-2}\right). \end{array}$$

Let us write

(A82) $$\psi_{a}=\exp\left(\pm i\nu_{p}^{2}\int dx\int_{a_{0}}^{a(x)}d\alpha \sqrt{-\frac{8}{3}\alpha^{4}\left(R\left(\alpha \right)-2\Lambda \right)}\right)\chi_{a}\equiv \exp\left(\pm i\nu_{p}^{2}S_{cl}\right)\chi_{a},$$

where

(A83) $$\chi_{a}=\exp\left(\pm i\frac{4}{3}{\left(c\hbar \right)}^{-1}\int dx\int_{a_{0}}^{a(x)}d\alpha \alpha H\left(\alpha ,x\right){\left(-\frac{8}{3}\alpha^{4}\left(R\left(\alpha \right)-2\Lambda \right)\right)}^{-\frac{1}{2}}\right)\psi_{a_{0}}.$$

Then, $S_{cl}$ in Equation (A82) satisfies the Hamilton–Jacobi equation

$$\int dx\frac{\delta S_{cl}}{\delta a(x)}\frac{\delta S_{cl}}{\delta a(x)}+\frac{8}{3}\int dxa^{4}\left(R\left(a\right)-2\Lambda \right)=0$$

and $\chi_{a}$ is the solution of the equation

(A84) $$\mp ic\hbar \int dx\frac{3}{4a(x)}\sqrt{-\frac{8}{3}a{\left(x\right)}^{4}\left(R\left(a\left(x\right)\right)-2\Lambda \right)}\frac{\delta \chi }{\delta a(x)}=\int dxH\left(a,x\right)\chi .$$

In this Schrödinger-type equation, there is a functional derivative over *a* instead of time. However, when we insert in the solution $\chi_{a}$ of Equation (A84) the classical solution a(t,x) instead of a(x), then we have

(A85) $$\partial_{t}\chi =\int dx\frac{da(t,x)}{dt}\frac{\delta \chi }{\delta a(t,x)}$$

We can replace the functional derivative in Equation (A84) by a time derivative if the classical solution satisfies the equation

(A86) $$\frac{c}{4a(t,x)}\sqrt{-\frac{8}{3}a{\left(t,x\right)}^{4}\left(R\left(a\left(t,x\right)\right)-2\Lambda \right)}=\frac{da(t,x)}{dt}.$$

Equation (A86) can be derived from the Hamilton–Jacobi formulation of general relativity [86]. As an example, consider a solution of Einstein equations without matter for the Robertson–Walker space-time metric with negative scalar curvature

(A87) $$ds^{2}=dt^{2}-h_{ij}\left(t,x\right)dx^{i}dx^{j}\equiv dt^{2}-a{\left(t,x\right)}^{2}dx^{2},$$

where $h_{ij}\equiv \delta_{ij}a{\left(t,x\right)}^{2}=\delta_{ij}a{\left(t\right)}^{2}(1-\frac{1}{4}{\left|x\right|}^{2}{)}^{-2}$. Then, $R\left(h\right)=-6a{\left(t\right)}^{-2}$. The solution of the Friedmann equation without matter is a(t)=ct, which agrees with Equation (A86). In another example, the solution $a\left(t\right)=\exp\left(\sqrt{\frac{\Lambda }{3}}t\right)$ of the spatially flat equation (A86) (R=0) describing de Sitter space without matter also leads to the replacement of the functional derivative in Equation (A84) by the time derivative.

Let us note that if R(a)−2Λ>0, then instead of the Schrödinger equation, we obtain a diffusion equation in the WKB expansion in powers of $\nu_{p}^{-2}$.
